# Borylation directed borylation of *N*-alkyl anilines using iodine activated pyrazaboles†

**DOI:** 10.1039/d3sc04269c

**Published:** 2023-10-17

**Authors:** C. R. P. Millet, E. Noone, A. V. Schellbach, J. Pahl, J. Łosiewicz, G. S. Nichol, M. J. Ingleson

**Affiliations:** a EaStCHEM School of Chemistry, University of Edinburgh Edinburgh EH9 3FJ UK michael.ingleson@edinburgh.ac.uk

## Abstract

Doubly electrophilic pyrazabole derivatives (pyrazabole = [H_2_B(μ-C_3_N_2_H_3_)]_2_) combined with one equiv. of base effect the *ortho*-borylation of *N*-alkyl anilines. Initial studies found that the bis(trifluoromethane)sulfonimide ([NTf_2_]^−^) pyrazabole derivative, [H(NTf_2_)B(μ-C_3_N_2_H_3_)]_2_, is highly effective for *ortho*-borylation, with this process proceeding through N–H borylation and then *ortho* C–H borylation. The activation of pyrazabole by I_2_ was developed as a cheaper and simpler alternative to using HNTf_2_ as the activator. The addition of I_2_ forms mono or ditopic pyrazabole electrophiles dependent on stoichiometry. The ditopic electrophile [H(I)B(μ-C_3_N_2_H_3_)]_2_ was also effective for the *ortho*-borylation of *N*-alkyl-anilines, with the primary C–H borylation products readily transformed into pinacol boronate esters (BPin) derivatives. Comparison of borylation reactions using the di-NTf_2_-and the diiodo-pyrazabole congeners revealed that more forcing conditions are required with the latter. Furthermore, the presence of iodide leads to competitive formation of side products, including [HB(μ-C_3_N_2_H_3_)_3_BH]^+^, which are not active for C–H borylation. Using [H(I)B(μ-C_3_N_2_H_3_)]_2_ and 0.2 equiv. of [Et_3_NH][NTf_2_] combines the higher yields of the NTf_2_ system with the ease of handling and lower cost of the iodide system generating an attractive process applicable to a range of *N*-alkyl-anilines. This methodology represents a metal free and transiently directed C–H borylation approach to form *N*-alkyl-2-BPin-aniline derivatives.

## Abstract 2

Doubly electrophilic pyrazabole derivatives (pyrazabole = [H_2_B(μ-C_3_N_2_H_3_)]_2_) combined with one equiv. of base effect the *ortho*-borylation of *N*-alkyl anilines.

## Graphical abstract

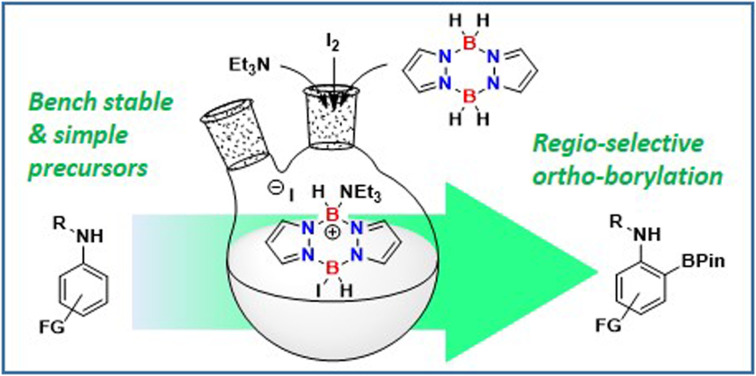

## Introduction

**Fig. 1:**
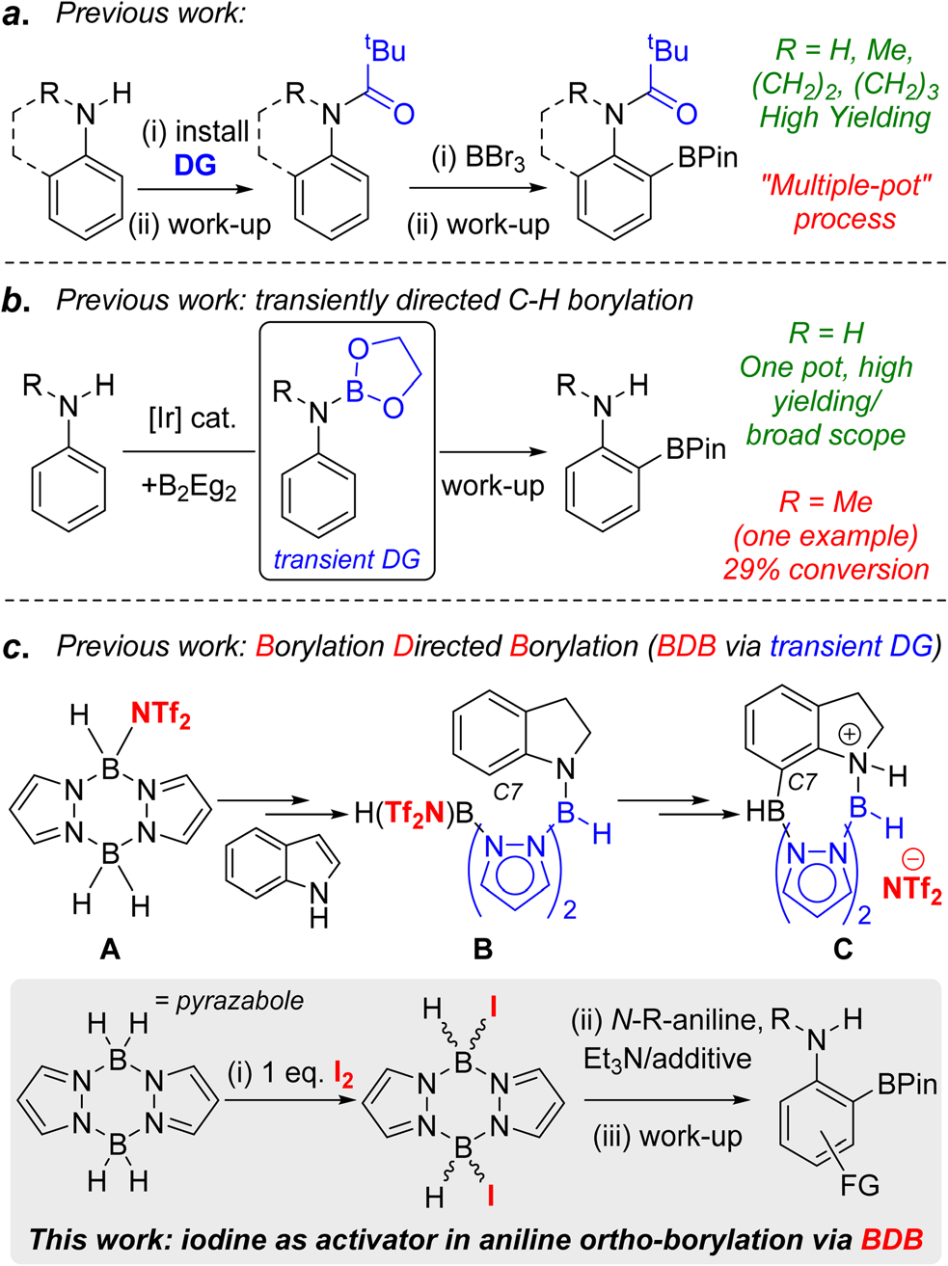
a = pivaloyl directed electrophilic borylation. b = a transient directing group in iridium catalysed *ortho*-borylation of anilines. c = previous work on indole reduction/C7 borylation *via* BDB. Inset bottom, this work.

C–H borylation is a powerful methodology for generating synthetically ubiquitous organoboranes in an efficient manner.^1^ The use of directing groups (DGs) in C–H borylation reactions enables access to organoboranes with a distinct regiochemistry to that formed from non-directed transformations.^2^ One specific example of this is in the synthesis of *ortho*-borylated anilines, which are useful for accessing *ortho* substituted anilines prevalent in pharmaceuticals, agrochemicals and organic materials.^3^ Directing groups generally are required for this *ortho* C–H borylation as in the absence of DGs the electrophilic C–H borylation of anilines leads to *para*-functionalisation,^4^ while iridium and cobalt catalysed C–H borylations generally lead to mixtures of *meta*- and *para*-borylated products.^1*b*,5^ To date, the *ortho* C–H borylation of anilines has been dominated by approaches requiring the separate installation and removal of a directing group (resulting in “multiple pot” processes).^6,7^ For example, the electrophilic *ortho* C–H borylation of aniline derivatives using *N*-pivaloyl DGs and BBr_3_ (Fig. 1a, top)^8^ requires the installation and removal of pivaloyl in separate processes, the latter under forcing conditions.^9^ The use of transient DGs is preferable as these are installed, direct the C–H borylation and then are removed all in one pot.^10^ In notable work, the *ortho*-borylation of anilines using transient DGs has been reported using iridium catalysts and B_2_Eg_2_ (Eg = ethylene glycolato).^11^ This proceeds *via in situ* formation of an ArylN(H)BEg species (Fig. 1b, inset) that then directs the *ortho* C–H borylation. The N-BEg unit is then readily cleaved during work-up. While this methodology is highly effective for ArylNH_2_ species, much lower yields (<30%) are obtained with *N*-alkyl-anilines.^11,12^ Given the prevalence of *ortho*-functionalised *N*-alkyl-anilines in pharmaceuticals (*e.g.* Flutemetamol, Entrectinib and Agratroban), the development of a higher yielding, transient DG approach for the *ortho*-borylation of *N*-alkyl-anilines is desirable, particularly if the process is precious metal-free.^13^

Recently, we reported the borylation directed borylation (BDB) of indoles using pyrazabole electrophile A (Fig. 1c) as a method to install boron units at the C7 position.^14,15^ In this process reduction of indole to indoline occurs first, with the spectroscopic data indicating that this led to an *N*-borylated indoline intermediate (*e.g.*B). The N–B bond and the pyrazabole structure in compound B positions the second boron centre appropriately to borylate the proximal sp^2^C–H leading to C, a C7 borylated indoline. Protection of the C–B unit and cleavage of the N–B bonds in C during work up formed indolines containing the useful pinacol boronate ester (BPin) group at C7. Therefore, pyrazabole is acting as a transient DG in this BDB process, with transient DGs underexplored in electrophilic C–H borylation.^2*a*,16^ Our initial BDB study utilised stoichiometric amounts of bistriflimidic acid (HNTf_2_ = HN(SO_2_CF_3_)_2_) to form the reactive electrophile A. However, HNTf_2_ is relatively expensive,^17^ and it, and NTf_2_-pyrazabole electrophiles (*e.g.*A), have to be handled within a glovebox. Therefore, extending the BDB of *N*-alkyl-aniline derivatives beyond indoline while using an inexpensive and more readily handled activator would be attractive. Herein we report our studies addressing this challenge. This led to the development of iodine as a cheap and easy to handle activator for pyrazaboles that forms ditopic electrophiles that are effective in the transient DG mediated *ortho*-borylation of *N*-alkyl-anilines.

## Results and discussion

**Scheme 1:**
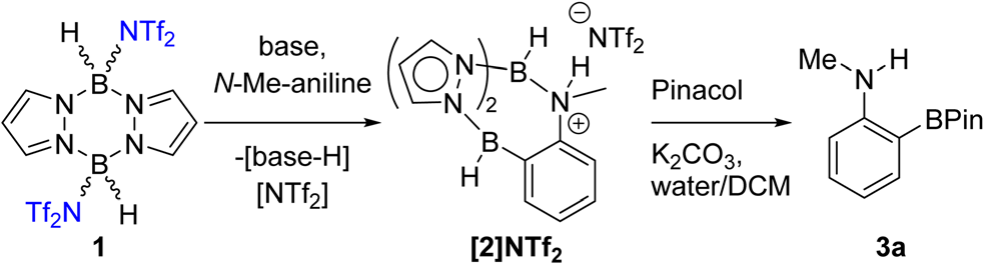
BDB of *N*-Me-aniline using 1 and an amine base.

Our first focus was identifying electrophilic pyrazabole – base combinations that achieved the *ortho*-borylation of our model substrate, *N*-Me-aniline. Initially, the previously reported 1 (Scheme 1) was added to *N*-Me-aniline in the presence of 2,6-di-*tert*-butyl-4-methylpyridine (DBP) as base. At room temperature this led to slow BDB, but on heating to ≥70 °C the BDB product [2]NTf_2_ was formed as the major product within 18 h. [2]NTf_2_ was fully characterised, which revealed protonation of the aniline nitrogen occurs during this BDB. A modified (shorter reaction time)^14^ N–B cleavage/pinacol installation process then led to formation of 3a.

DBP is an expensive Brønsted base that was used to simplify initial studies as it does not coordinate to boron electrophiles. In contrast, other Lewis bases (*e.g.* MeCN) can displace NTf_2_ anions from 1, and base coordination to boron could retard the BDB reaction.^14^ Given the aniline substrate also functions as a Brønsted base during BDB (as indicated by the formation of [2]NTf_2_) only one equivalent of exogenous base is required. Therefore, one equivalent of the inexpensive bases Et_3_N and Hünigs base were trialled in place of DBP in the BDB of *N*-Me-aniline using 1. On heating both of these reactions led to the formation of [2]NTf_2_ and [baseH][NTf_2_] as a by-product. Pinacol installation/work-up enabled 3a to be isolated in 62 and 65% yield using Et_3_N and Hünigs base, respectively. Thus cheaper (than DBP) bases can be used in the BDB of *N*-alkyl-anilines. Our attention turned next to replacing HNTf_2_ with a simpler to handle and cheaper activator.

**Scheme 2:**
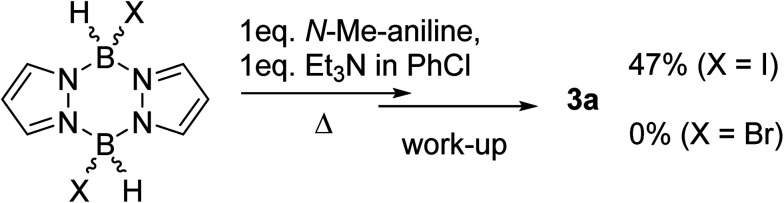
Outcomes using dibromo-*versus* diiodo-pyrazabole in the BDB of *N*-Me-aniline.

Based on the established reactivity of L→BH_3_ with iodine, which forms reactive boron electrophiles of general formula L→BH_2_I,^18^ diiodo-pyrazabole was targeted as an alternative to 1. While dibromo- and dichloro-pyrazaboles are known,^19^ to our knowledge no B–I containing pyrazaboles have been reported to date. The latter are desirable as iodine is inexpensive, easy to handle and is less coordinating to boron than the lighter halides. Furthermore, L→BH_2_I species have been demonstrated to react with π nucleophiles to form C–B bonds in a related manner to L→BH_2_(NTf_2_) species.^20^ Therefore, one equivalent of iodine, pyrazabole and Et_3_N were combined and found to be viable for the BDB of *N*-Me-aniline (Scheme 2), albeit requiring heating to 100 °C for significant BDB to occur. In contrast, attempts using dibromo-pyrazabole under identical conditions led to no BDB reaction (Scheme 2), indicating that the less coordinating nature of iodide towards boron is vital for this transformation. Despite extensive optimisation studies using iodine activated pyrazabole (see Table S2†) the isolated yield of 3a remained <50% (based on *N*-Me-aniline) – with Et_3_N providing the best outcome from the bases explored. Notable points from this optimisation study included: use of >1 equiv. of Et_3_N retarding the BDB reaction, while using two equiv. of *N*-Me-aniline and no other base gave only trace amounts of 3a. Given the lower yields of 3a using iodine activated pyrazabole relative to using 1, both systems were analyzed further to determine the origin(s) of this disparity.

### Mechanistic studies

**Scheme 3:**
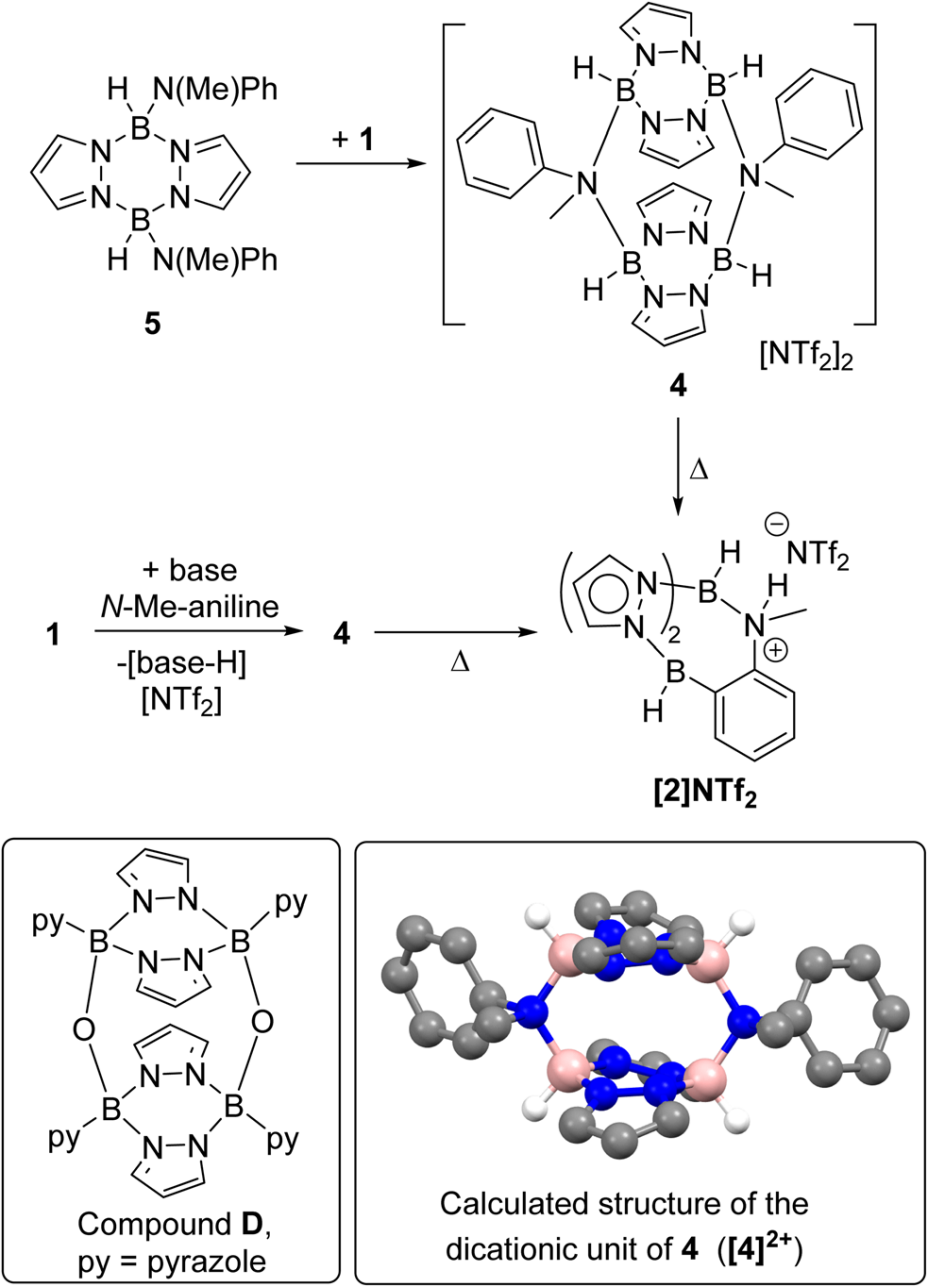
Top, the formation of 4, from combination of 1 and 5; middle, the formation of 4 during BDB and its subsequent conversion into [2]NTf_2_. Inset bottom left, the previously reported oxo bridged dimeric pyrazabole, D; inset bottom right the calculated structure of [4]^2+^ showing only the hydrogens bonded to boron for clarity, pink = boron, blue = nitrogen, grey = carbon, white = hydrogen.

On analysing the reaction of 1 and one equiv. base (base = DBP or Et_3_N) with *N*-Me-aniline by *in situ* NMR spectroscopy an intermediate was observed. This intermediate, termed 4, could be obtained cleanly by the combination of 1 and the independently synthesised di(N–Me-anilide)-pyrazabole 5 (Scheme 3, see ESI Section 2 and Fig. S14–S20†). Compound 4 displayed two pyrazabole C–H resonances in the ^1^H NMR spectrum in a 2 : 1 ratio indicating a symmetrically substituted pyrazabole. Further insight into the structure of 4 came from ^19^F NMR spectroscopy, which revealed NTf_2_ is not coordinated to boron (*δ*_19F_ = −78.7, whereas for B-NTf_2_ systems *δ*_19F_ ≈ −69),^21^ and DOSY NMR studies (see ESI, Section 5.3†) which indicated 4 is dimeric. The dimeric structure for 4 presumably is related to the previously reported oxo-bridged dimer D (Scheme 3),^22^ with an analogous structure fully consistent with the NMR data for 4 (as a single isomer with a *cis* arrangement of the aniline-*N* substituents). Compound 4 converted into the BDB product [2]NTf2 slowly at ambient temperature, but more rapidly and in high conversion on heating. While 4 could not be isolated as single crystals suitable for diffraction studies the structure of the dicationic portion of 4 (termed [4]^2+^) was calculated at the MN15/6-311G(d,p)/PCM (PhCl, PCM = polarizable continuum model) level (inset Scheme 3, note all calculations are performed at this level herein, with the LANL2DZ basis set used for iodide). While the B–N distances in the calculated structure (1.615–1.618 Å) are comparable to related borocations,^23^ there is evidence for significant distortion in [4]^2+^ due to steric interactions between the pyrazole rings and the *N*-Me and *N*-Ph substituents. For example, the _Ph_C–N–C_Me_ angle is small (102.7° in [4]^2+^) while the B_2_N_4_ core is twisted (in the B_2_N_4_ core of D the four nitrogens are co-planar, however in [4]^2+^ they deviate by upto 0.11 Å above and below the plane made by the four nitrogens). These distortions will destabilise dimeric [4]^2+^ presumably enabling dissociation into a monomeric form that is required to effect *ortho* C–H borylation.

**Scheme 4:**
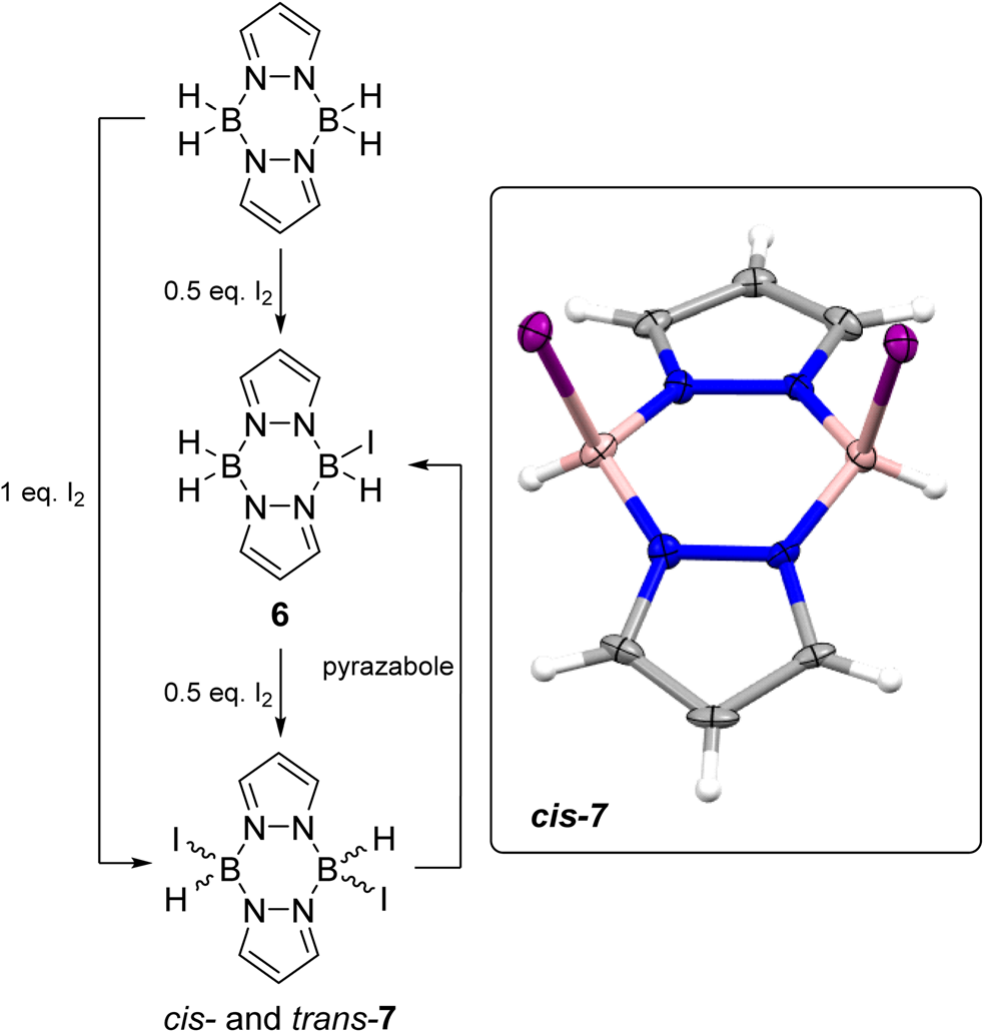
Left, formation of mono- (6) and diiodo-pyrazabole (7). Inset right, the solid state structure of *cis*-7, ellipsoids at 50% probability. Blue = nitrogen, pink = boron, purple = iodine, grey = carbon, white = hydrogen.

Moving to the iodo-pyrazaboles, the reaction of pyrazabole and iodine was investigated first as iodo-pyrazaboles have not been reported previously to our knowledge. The addition of 0.5 equiv. of I_2_ to pyrazabole led to the rapid formation of the mono-iodo pyrazabole, 6 (Scheme 4) at room temperature (by *in situ* NMR spectroscopy, Fig. S44†). Addition of a further 0.5 equiv. of iodine led to the full conversion of 6 into the diiodo pyrazabole, 7. Compound 7 is formed as a *ca.* 1 : 1 mixture of isomers as indicated by two doublets in the ^11^B NMR spectrum along with two sets of 2 : 1 relative integral pyrazole resonances in the ^1^H NMR spectrum, which is consistent with two symmetrically substituted pyrazaboles. These isomers are assigned as the *cis* and *trans* isomers of 7 based on previous reports from the groups of Trofimenko and Nöth on *cis* and *trans* isomers being formed for the lighter dihalo pyrazaboles.^24,25^ Calculations also indicated that the *cis* and *trans* isomers of 7 are close in energy (*ca.* 1 kcal mol^−1^ calculated free energy difference), consistent with the two species observed in solution being the *cis* and *trans* isomers of 7. The addition of one equiv. of I_2_ in one portion to pyrazabole also led to the formation of 7 and it was isolated in 75% yield. The *cis* isomer formed single crystals suitable for X-ray diffraction studies. The solid-state structure of the *cis* isomer of 7 has a B_2_N_4_ 6-membered core in a flattened boat conformation with the iodide substituents located in the flagpole positions. In 7 the B⋯B distance of 3.031(8) Å is in the expected region and is comparable to a related dihalogenated pyrazabole [H(Br)B(μ-C_3_N_2_H_2_Cl)]_2_ (3.05 Å).^25^ The B–I bond distances of 2.290(6) and 2.302(6) Å are at the lower end of B–I bond lengths reported for L→BH_2_I compounds (L = *N*-heterocyclic carbenes or PR_3_).^26^ Notably, combining equimolar 7 and pyrazabole in chlorobenzene led to formation of the mono-iodo pyrazabole 6 at ambient temperature (by *in situ* NMR spectroscopy - Fig. S48†), indicating that intermolecular H/I exchange occurs in iodo-pyrazaboles. Finally, it should be noted that 7 has appreciable thermal stability: heating 7 at 100 °C in PhCl for 3 days led to minimal decomposition (<5% by multinuclear NMR spectroscopy), with the only observable new ^11^B NMR resonance consistent with formation of an L-BI_3_ compound (based on the *δ*_11B_ = −34.6, see Fig. S46 and S47†).

**Scheme 5:**
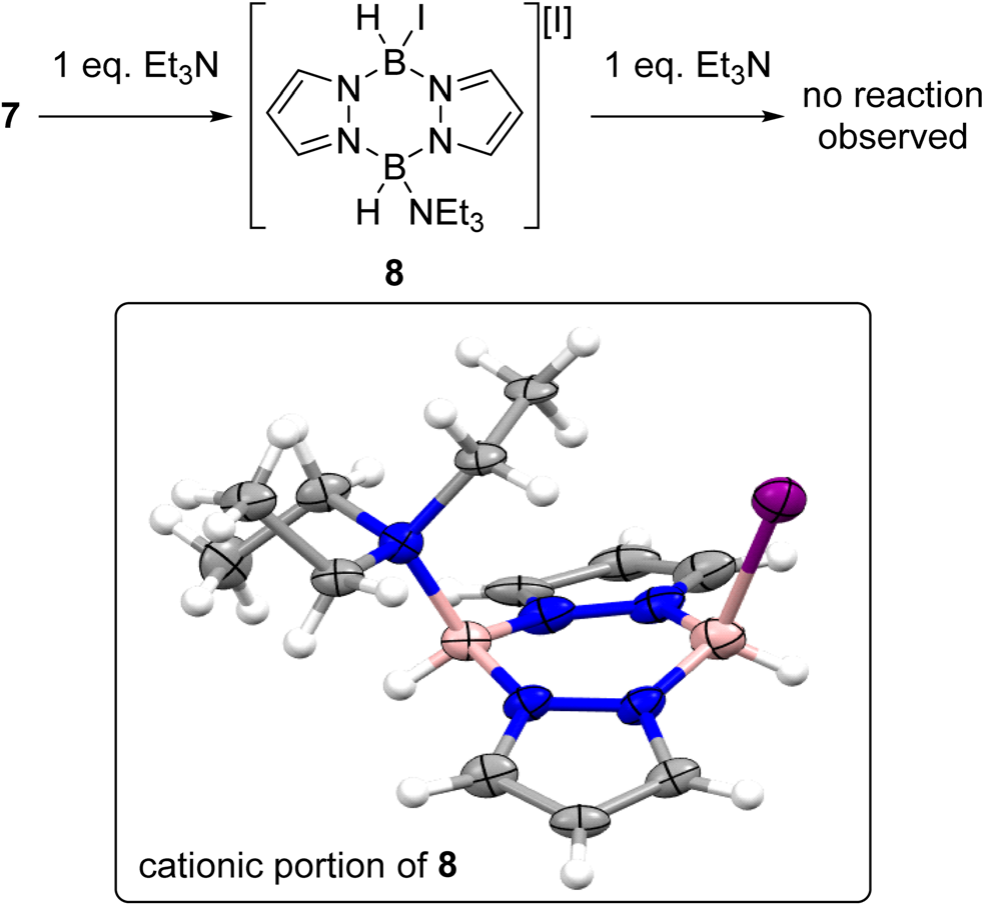
Top, the reaction of 7 towards Et_3_N. Inset bottom, the solid-state structure of the cationic portion of 8, ellipsoids at 30% probability. Blue = nitrogen, pink = boron, purple = iodine, grey = carbon, white = hydrogen.

With an understanding of the products formed from combining iodine and pyrazabole in hand the reactivity of 7 towards Et_3_N was explored, Et_3_N was selected as it gave the best outcome in our initial optimisation study (see Table S2†). The addition of one equivalent of Et_3_N to 7 led to formation of the mono-cation 8 (Scheme 5). The identity of 8 was confirmed by single crystal X-ray diffraction analysis (inset, Scheme 5). The solid-state structure of 8 also has a flattened boat conformation for the B_2_N_4_ core with the iodide and Et_3_N moieties being *cis* in the flagpole positions. The steric demand of Et_3_N in 8 causes a distortion in the geometry with an increase of the Y–B-*Centroid* angles (Y = I or N_Et3_; centroid *=* calculated centroid of the B_2_N_4_ ring) observed on comparing 7 (I–B-centroid = 113.3(3)° and 112.6(3)°) and 8 (I–B-centroid = 118.6(12)°; Et_3_N–B-centroid = 122.1(14)°). Compound 8 also has a longer B–I bond of 2.36(2) Å *vs.* the B–I bonds in 7 (2.290(6) and 2.302(6) Å), consistent with greater steric crowding in 8 relative to 7. However, the B–N_Et_3__ bond length in 8 (1.62(2) Å) is in the range of previously reported Et_3_N-BR_3_ adducts (1.60–1.69 Å).^27^

**Scheme 6:**
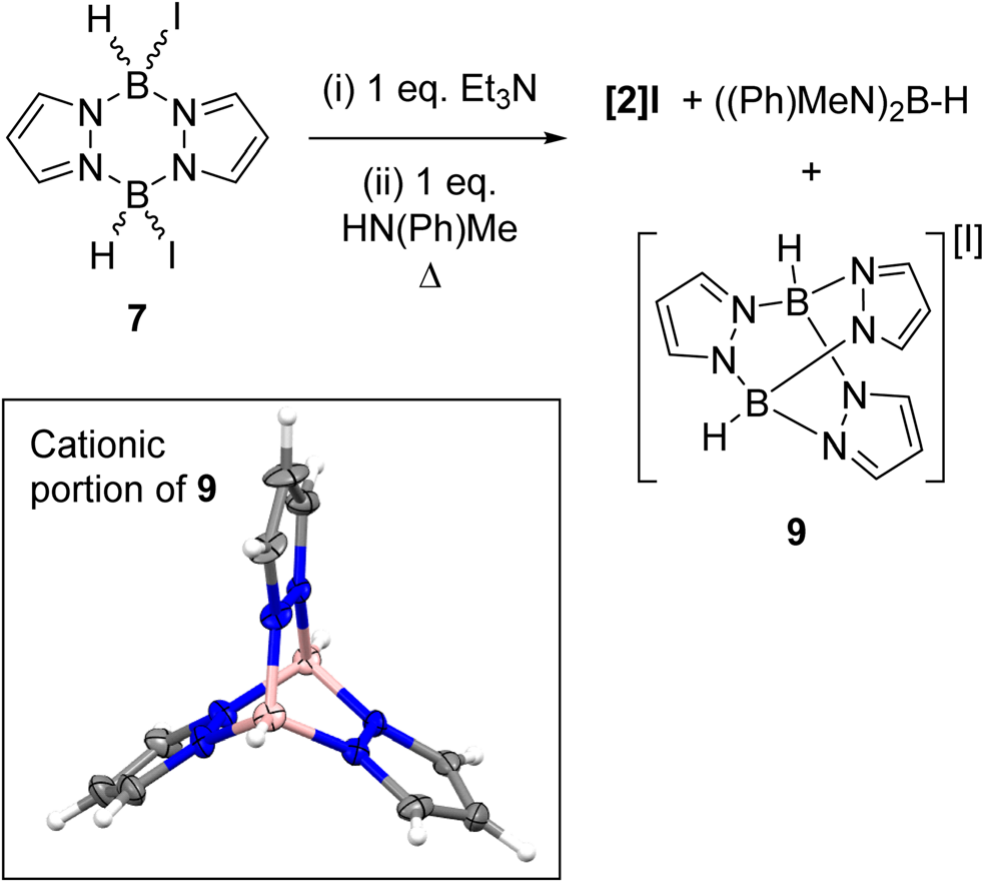
Left, the reactivity of 7 towards Et_3_N/*N*-Me-aniline at raised temperatures. Inset, the solid-state structure of compound 9, ellipsoids at 30% probability. Blue = nitrogen, pink = boron, grey = carbon, white = hydrogen.

In contrast to the di-NTf_2_ analogue 1 (where both NTf_2_ anions are displaced by Lewis bases to form dicationic products),^14^ the addition of further Et_3_N to 8 did not displace the second iodide (Fig. S49†). This is consistent with the more coordinating nature of iodide relative to [NTf_2_]^−^. However, the addition of both *N*-Me aniline and Et_3_N (in either order of addition) to 7 led to substitution of both iodides to form the di-anilide product 5 as the major boron containing species. This indicates that Et_3_N coordination to boron in 8 does not irreversibly block *N*-Me-aniline from reacting with boron. Next, diiodo-pyrazabole 7 and dianilide-pyrazabole 5 were combined to determine if the iodide analogue of the dimer 4 forms. This led to slow and complex reactivity at room temperature with no iodide analogue of 4 observed. In contrast, the di-NTf_2_ pyrazabole 1 and compound 5 are completely consumed within minutes of mixing to form 4 cleanly. In the *in situ* monitored BDB reactions using diiodo-pyrazabole 7, 5 is the only major new pyrazabole product observed, again there is no evidence for the iodide analogue of 4 (by NMR spectroscopy). From the *in situ* monitoring experiments [2]I forms as one of the major products on heating, but this occurs along with the formation of two other major products. The first of these was assigned as (Me(Ph)N)_2_BH (*δ*_11B_ = 29.0 ^1^*J*_B–H_ = 126 Hz) by comparison to the previous report.^28^ The second was identified as compound 9 (Scheme 6), which precipitated from the BDB reactions mixtures (along with some [Et_3_NH][I] precipitating). Compound 9 was independently synthesised and crystallised with X-ray diffraction studies confirming its formulation (inset Scheme 6). These results combined indicate that heating diiodo-pyrazabole 7 in the presence of Et_3_N/*N*-Me-aniline leads to competitive (to BDB) break-up of the pyrazabole core and the formation of species that are non-productive for BDB (*e.g.* compound 9). This contrasts with BDB using the NTf_2_ derivative 1 (which are much cleaner by *in situ* NMR spectroscopy with <5% formation of other pyrazole containing products by NMR spectroscopy), indicating that the more coordinating iodide anion plays a crucial role in the cleavage of the pyrazabole core under these conditions. This is presumably the origin of the lower conversions to [2]I (and thus 3a) observed using 7 compared to conversions to [2]NTf_2_ using the NTf_2_ analogue 1.

Given the lower conversion to 3a using 7 relative to that using stoichiometric 1, attempts were made to use sub-stoichiometric HNTf_2_ (or sub-stoichiometric 1) and stoichiometric pyrazabole in the BDB of *N*-Me aniline. However, these reactions all led to low yields of 3a, this is consistent with the observation that [Et_3_NH][NTf_2_] (the by-product from BDB) and pyrazabole do not react on heating to 100 °C. Therefore alternative approaches were sought to achieve a high yielding, operationally simple and cheaper BDB protocol.

### Optimisation of the BDB of *N*-alkyl-anilines using iodo-pyrazaboles

**Scheme 7:**
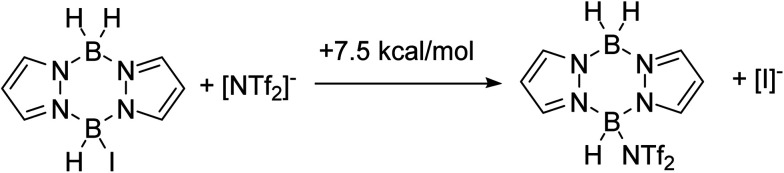
Free energy change for iodide/[NTf_2_]^−^ exchange.

To combine the best of the NTf_2_ (higher yields) and iodide (cheaper/easier to handle) systems we considered an *in situ* anion exchange process that could convert iodo-pyrazaboles into more reactive NTf_2_-pyrazaboles. The feasibility of iodide/NTf_2_ exchange initially was explored computationally which indicated that the displacement of iodide from pyrazabole by triflimide is endergonic (by +7.5 kcal mol^−1^ for the mono-pyrazabole, Scheme 7). This is consistent with the addition of 5 equiv. of [Et_3_NH][NTf_2_] to 7 resulting in no observable anion exchange (by NMR spectroscopy). Nevertheless, as the BDB process has a significantly lower overall barrier for the NTf_2_ system relative to the iodide analogue (1 performs BDB at room temperature, albeit slowly, while 7 requires heating to ≥70 °C for BDB) anion exchange may still lead to an enhanced BDB outcome. Note, a related anion exchange process facilitating an electrophilic C–H borylation with B-trypticenes has been reported recently using stoichiometric Na[B(C_6_F_5_)_4_].^29^

**Scheme 8:**
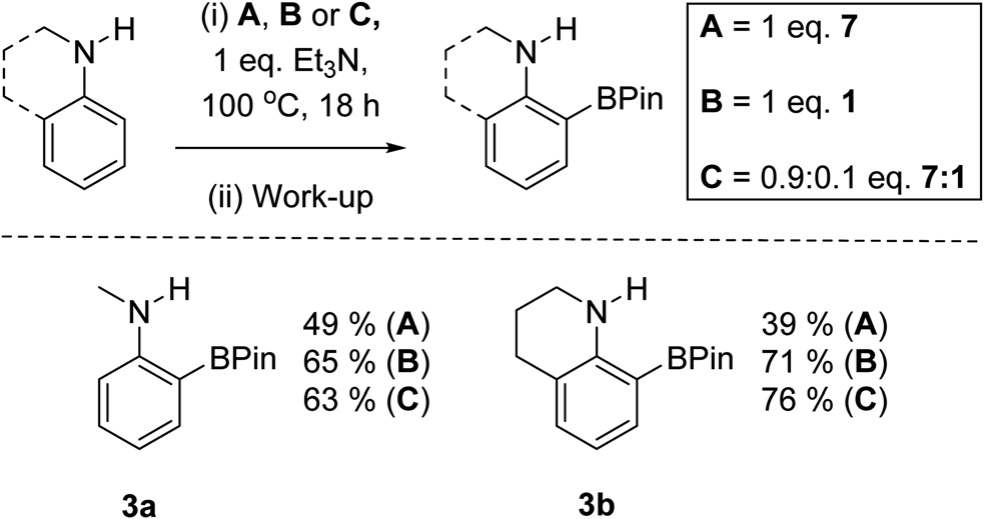
Outcomes from using 1, 7 or 1/7 in the BDB reaction.

An initial experiment to assess for any anion exchange derived enhancement in yield used a 0.9 : 0.1 mix of 7 : 1 in the BDB of *N*-Me-aniline with one equiv. of Et_3_N as base. Notably, this led to comparable yields for the formation of 3a (Scheme 8) to that using 1 equiv. of 1. A significant yield enhancement was also observed using a 0.9 : 0.1 mix of 7 and 1 in the BDB of tetrahydroquinoline to form 3b post pinacol installation/work-up (Scheme 8). The significant yield enhancement observed using 0.9 : 0.1 mixtures of 7 and 1 indicates it is not just due to compounds 7 and 1 reacting separately in the BDB process. We tentatively attribute this enhancement to a degree of metathesis of an iodo-pyrazabole with [Et_3_NH][NTf_2_] (formed during BDB) leading to a more reactive NTf_2_-pyrazabole electrophile. Note, during these reactions in chlorobenzene solid precipitates, which on analysis was found to be [Et_3_NH][I]. Thus the lower solubility of [Et_3_NH][I] relative to the NTf_2_ salt under these conditions may be assisting anion exchange. The precipitation of [Et_3_NH][I] also will reduce the iodide concentration in solution, potentially slowing the formation of decomposition species. This is consistent with the observation that compound 9 is not observed during the reactions using 0.9 : 0.1 of 7 and 1.

**Scheme 9:**
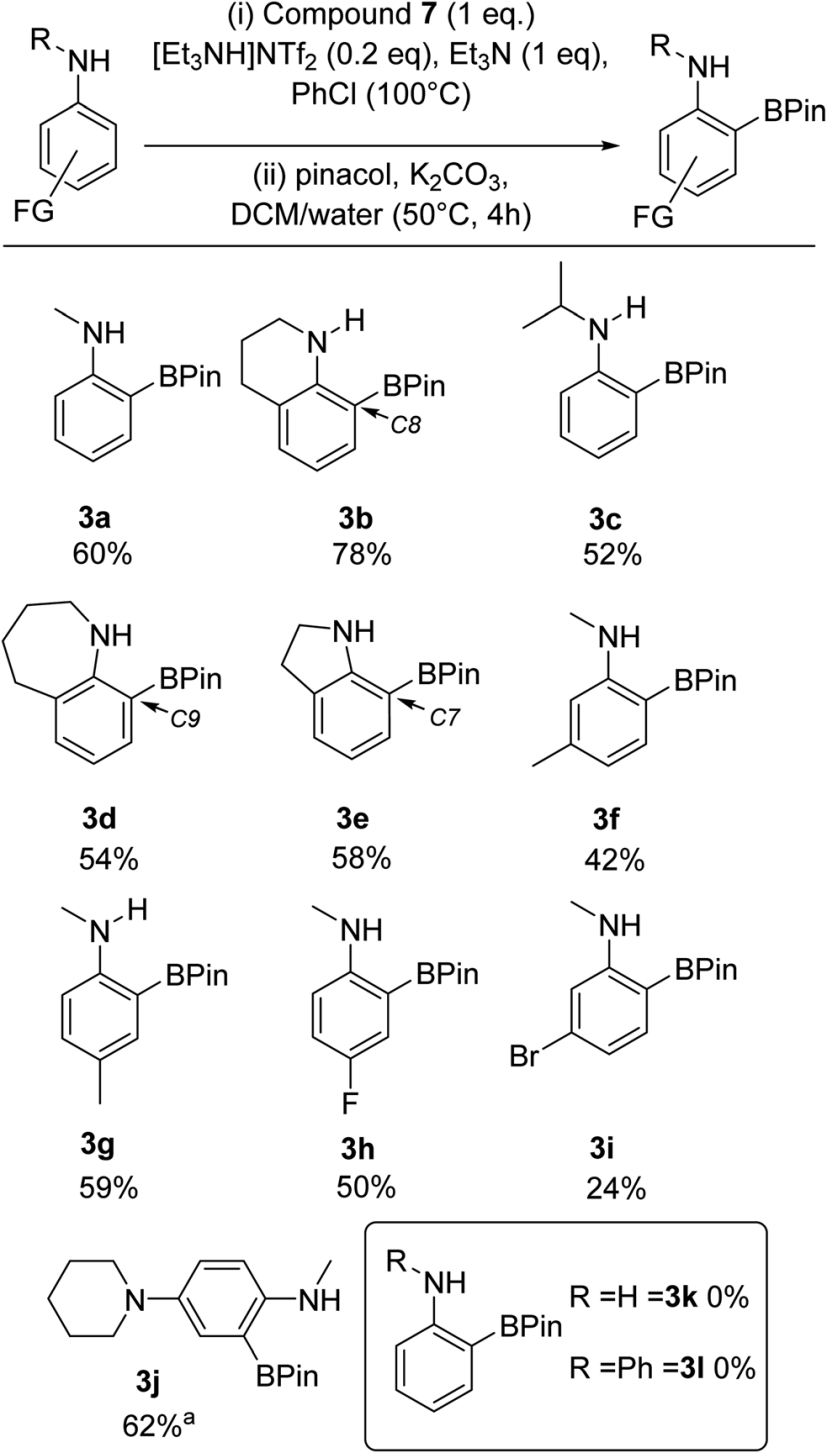
Substrate scope and isolated yields (unless otherwise stated) for the BDB of aniline derivatives using 7/Et_3_NH[NTf_2_]. a = conversion *versus* an internal standard.

Overall, these observations suggested that combining 7 with sub-stoichiometric [cation][NTf_2_] could result in a similar enhancement in yield. This hypothesis was confirmed by the use of one equiv. of 7 and 0.2 equiv. of [Et_3_NH][NTf_2_] in the BDB process leading to a 60% yield of 3a and a 78% yield of 3b (comparable to outcomes from conditions B and C in Scheme 8). This is a notable improvement over the yields reported using iridium catalysed transient DG approaches to form *ortho*-BPin-*N*-alkyl-anilines.^11,12,30^ Note, the use of 0.2 equiv. of LiNTf_2_ with 7 gave lower yields relative to using [Et_3_NH][NTf_2_] under otherwise identical conditions, therefore the latter salt is used hereon. With conditions identified that avoided expensive bases and stoichiometric amounts of anhydrous HNTf_2_ ([Et_3_NH][NTf_2_] can be stored on the bench and is readily accessible from commercial LiNTf_2_ and [Et_3_NH][Cl]) a substrate scope exploration was performed (Scheme 9). The scoping study revealed that in addition to 3a and 3b the conditions were amenable to larger alkyl substituents on nitrogen, with the N-^i^Pr derivative, 3c, isolated in 52% yield. Alongside 3b, the seven (3d) and five (3e) membered analogues were also amenable to BDB, indicating the change in positioning of the N-bound pyrazabole unit enforced by the different ring sizes does not significantly influence this BDB reaction. Notably, neither 3d nor any other C9 borylated benzo[*b*]azepines have been reported previously to our knowledge. This is despite the significant importance of substituted benzo[*b*]azepines in pharmaceuticals and agrochemicals, including C9-substitued derivatives (*e.g.* zilpaterol).^31^ In contrast, the *ortho*-methyl derivative, 2,*N*-Me_2_-aniline, was not amenable to this process. We attribute this to the *ortho* methyl forcing an orientation that disrupts conjugation between the aniline phenyl ring and the nitrogen lone pair. This was supported by calculations on analogues of 5 containing 2,*N*-Me_2_-aniline (twisted away from co-planarity by 44°) and indoline and tetrahydroquinoline (see Table S4†) – with the latter two compounds and 5 having close to co-planar N and phenyl units that maximise conjugation and thus increase the nucleophilicity of the π system (thereby favouring S_E_Ar).

Moving to other substituents, as this is an electrophilic borylation using borenium cation equivalents and forcing conditions, functional group tolerance will be limited (as indicated by the *p*-MeO derivative not being amenable to this process),^23^ but halides and NR_2_ groups are tolerated (*vide infra*). Furthermore, while the *ortho* methyl aniline derivative was not amenable substituents at the *meta* (3f and 3i) and *para* (3g and 3h) positions of *N*-Me-aniline were tolerated. This BDB process was found to be sensitive to arene electronics, with electron withdrawing groups significantly retarding BDB, requiring longer reaction times for 3h and 3i. Consistent with this observation, an *N*-Me-aniline substrate substituted with an electron donating group, specifically a *para*-piperidine unit, performed much better in this BDB process, with 3j isolated in 62% yield. *Ortho*-substituted anilines containing a *para*-piperidine unit are important as these motifs are found in approved and developmental bioactives, *e.g.* Brigatinib and ASP3026.^32^ Next, we attempted to extend this BDB process to aniline and diphenylamine. However, in both cases no *ortho* borylated products (3k and 3l) were isolated. While diphenylamine is presumably insufficiently nucleophilic for this BDB reaction (consistent with an S_E_Ar type process), the origin of the incompatibility of aniline with this BDB reaction is currently unclear. Finally, we assessed the amenability of this methodology to scaling and glovebox free conditions: compound 3a was isolated in 62% yield when the BDB process was scaled up ten-fold, while 3a was isolated in 45% yield under glovebox free conditions (making 7*in situ* from bench stable pyrazabole and iodine, note pyrazabole itself is readily accessed from pyrazole and L→BH_3_).^19^

## Conclusions

Iodine is an inexpensive activator for pyrazaboles that forms mono- and di-topic pyrazabole electrophiles, with the latter effective in the borylation directed borylation (BDB) of *N*-alkyl anilines. However, when using diiodo-pyrazabole 7 competitive formation of inactive (for BDB) species occurs that arise from break-up of the B_2_N_4_ pyrazabole core. This leads to lower BDB conversions using 7 than when using the di-NTf_2_ pyrazabole analogue 1 (which reacts with <5% of unwanted side products by NMR spectroscopy). The attractive features of both systems (iodine = cheaper and easy to handle activator, while NTf_2_-pyrazaboles = higher conversions in BDB) can be combined by using the diiodo-pyrazabole 7 in combination with 0.2. equiv. of [Et_3_NH][NTf_2_]. This BDB methodology is operationally simple (no glovebox required) and is applicable to a range of *N*-alkyl anilines. The primary BDB products can be readily transformed into synthetically ubiquitous pinacol boronates esters, thus this process represents a metal-free transient directed C–H borylation methodology to form desirable *N*-alkyl-2-BPin-anilines.

## Data availability

The data supporting this article has been uploaded as part of the ESI,† this includes NMR spectra for all new compounds, *in situ* NMR spectra for catalytic and mechanistic reactions and Cartesian coordinates for all calculated structures.

## Author contributions

MI, and CM conceived the research concept and aims and analysed all data. CM performed the majority of the synthetic work and the majority of the analytical components of this project. EN, AS, and JP also performed the synthesis and characterisation of a number of compounds reported in this manuscript. GN and JP collected and solved all the crystal structures. JL performed a number of the calculations. Combined, MI, CM and EN drafted, reviewed and edited the manuscript.

## Conflicts of interest

There are no conflicts to declare.

## Supplementary Material

SC-014-D3SC04269C-s001

SC-014-D3SC04269C-s002
