# Supplementary material for: Borylation directed borylation of N-alkyl anilines using iodine activated pyrazaboles
Source: Chem Sci. 2023 Oct 17;14(43):12041–8. doi: 10.1039/d3sc04269c (PMC10631245; doi:10.1039/d3sc04269c)
Supplement: SC-014-D3SC04269C-s001 [file SC-014-D3SC04269C-s001.pdf]

Electronic Supplementary Material (ESI) for Chemical Science.
This journal is © The Royal Society of Chemistry 2023

Borylation directed borylation of N-alkyl anilines using iodine activated pyrazaboles.

C. R. P. Millet, E. Noone, A. V. Schellbach, J. Pahl, J. Łosiewicz, G. S. Nichol, M. J. Ingleson*

School of Chemistry, University of Edinburgh, Edinburgh, EH9 3FJ.

Contents

1 General considerations 5

2 Comment on isomerism of pyrazabole derivatives 6

3 Preparation of the pyrazabole derivatives, triflimide salt and substrate precursors .8

3.1 Preparation of diiodo-pyrazabole 7 8

3.2 Preparation of dibromo-pyrazabole 9

3.3 Preparation of di-N-methylaniline-pyrazabole 5 10

3.4 Preparation of mono-cation 8 12

3.5 Preparation of $[Et_3NH]NTf_2$ 13

3.6 Preparation of 4-piperidine-N-methylaniline 14

3.7 NMR spectra of isolated and novel precursors 15

3.7.1 NMR spectra of diiodo-pyrazabole 7 15

3.7.2 NMR spectra of dibromo-pyrazabole 17

3.7.3 NMR spectra of di-N-methylaniline-pyrazabole 5 19

3.7.4 NMR spectra of mono-cation 8 22

3.7.5 NMR spectra of 4-piperidine-N-methylaniline 23

4 Preliminary studies 24

4.1 BDB with di-NTf_2-pyrazabole 1 24

4.1.1 Base scope in BDB with di-NTf_2-pyrazabole 1 24

4.1.2 Characterisation of $[2]NTf_2$ 25

4.1.3 NMR spectra of the $[2]NTf_2$ 26

4.1.4 In situ generation of $[2]NTf_2$ 29

4.2 BDB with diiodo-pyrazabole 7 31

4.2.1 General procedure 1 31

4.2.2 Results 32

4.3 BDB with dibromo-pyrazabole 33

5 Mechanistic studies 34

5.1 Characterisation of compound 4 34

5.2 NMR spectra of compound 4 35

5.3 DOSY experiments 37

5.3.1 DOSY of di-N-methylaniline-pyrazabole 5 37

5.3.2 DOSY of 4 39

5.4 In situ generation of compound 4 41

5.5 Study of the diiodo-pyrazabole 7 43

5.5.1 Conversion study and in-situ monitoring 43

5.5.2 *Stability study* ... 46
5.5.3 *Reaction between Diiodo-pyrazabole 7 and pyrazabole* ... 48
5.5.4 *Reaction between Diiodo-pyrazabole 7 and Et_3N (1 or 2 equiv.)* ... 50
5.5.5 *Order of addition study* ... 52
5.5.6 *BDB attempt at 70 °C: in situ observation of B-5* ... 55
5.5.7 *In situ observation of borylated intermediate [2]I* ... 57
5.5.8 *Generation of compound 9* ... 60
5.5.9 *In situ formation of compound 9* ... 63
5.5.10 *Reaction between diiodo-pyrazabole 7 and $[Et_3NH]NTf_2$* ... 64
5.6 Hygroscopicity check of $[Et_3NH]NTf_2$ ... 66
6 BDB comparison: 7 *vs* 1 *vs* combined 7/catalytic 1 ... 68
7 Control reactions ... 70
7.1 BDB of N-methylaniline attempt from pyrazabole with catalytic $[Et_3NH]NTf_2$ ... 70
7.2 Hydrogen formation assessment ... 70
8 BDB with alternative systems ... 72
8.1 Diiodo-pyrazabole + Cat. $LiNTf_2$ ... 72
8.2 Dibromo-pyrazabole + Cat. $[Et_3NH]NTf_2$ ... 72
9 BDB with 7/cat $[Et_3NH]NTf_2$ ... 73
9.1 General procedure 2 ... 73
9.2 Substrate scope ... 74
9.2.1 *N-methyl-2-(BPin)aniline 3a* ... 74
9.2.2 *8-(BPin)-1,2,3,4-tetrahydroquinoline 3b* ... 74
9.2.3 *N-isopropyl-2-(BPin)aniline 3c* ... 75
9.2.4 *9-(BPin)-2,3,4,5-Tetrahydro-1-benzo[b]azepine 3d* ... 76
9.2.5 *7-(BPin)indoline 3e* ... 77
9.2.6 *3-N-dimethyl-2-(BPin)aniline 3f* ... 78
9.2.7 *4-N-dimethyl-2-(BPin)aniline 3g* ... 78
9.2.8 *4-fluoro-N-methyl-2-(BPin)aniline 3h* ... 79
9.2.9 *3-bromo-N-methyl-2-(BPin)aniline 3i* ... 80
9.2.10 *4-piperidine-N-methyl-2-(BPin)aniline 3j* ... 81
9.3 Scaled-up preparation of N-methyl-2-(BPin)aniline 3a ... 82
9.4 Glovebox free preparation of N-methyl-2-(BPin)aniline 3a ... 82
9.5 NMR Spectra ... 84
9.5.1 *NMR spectra of N-isopropyl-2-(BPin)aniline 3c* ... 84
9.5.2 *NMR spectra of 9-(BPin)-2,3,4,5-Tetrahydro-1-benzo[b]azepine 3d* ... 85
9.5.3 *NMR spectra of 3-N-dimethyl-2-(BPin)aniline 3f* ... 86
9.5.4 *NMR spectra of 4-N-dimethyl-2-(BPin)aniline 3g* ... 87

9.5.5 NMR spectra of 4-fluoro-N-methyl-2-(BPin)aniline 3h *89*
9.5.6 NMR spectra of 3-bromo-N-methyl-2-(BPin)aniline 3i *90*
9.5.7 NMR spectra of 4-piperidine-N-methyl-2-(BPin)aniline 3j *91*
10 Crystallographic data 93
10.1 Crystal structure of di-N-methylaniline-pyrazabole 5 93
10.2 Crystal structure of diiodo-pyrazabole 7 94
10.3 Crystal structure of mono-cation 8 95
10.4 Crystal structure of compound 9 96
11 Computational details 98
11.1 Comparison of *cis/trans*-diiodo-pyrazabole 7 isomer 98
11.1.1 Trans-diiodo-pyrazabole isomer (trans-7) *98*
11.1.2 Cis-diiodo-pyrazabole isomer (cis-7) *99*
11.2 Calculated dicationic dimeric structure ($[4]^{2+}$) 100
11.3 Calculation of iodide/NTf_2 exchange 103
11.3.1 Anion [NTf2]$^-$ *103*
11.3.2 Anion Iodide *104*
11.3.3 Mono-NTf_2-pyrazabole *104*
11.3.4 Monoiodo-pyrazabole *106*
11.4 Calculation of the di-N-alkyl-anilines-pyrazabole 107
11.4.1 Di-N-methylaniline-pyrazabole *107*
11.4.2 Di-2,N-dimethylaniline-pyrazabole *109*
11.4.3 Di-indoline-pyrazabole *111*
11.4.4 Di-tetrahydroquinoline-pyrazabole *114*
11.4.5 Angle comparison in compound 5 and its analogues *117*
12 References 119

1 General considerations

All reactions were performed under inert conditions using standard Schlenk techniques or in an *MBraun Unilab* glovebox (<0.1 ppm H_2O / O_2).

Unless otherwise stated, solvents were degassed with nitrogen, dried over activated aluminium oxide (Solvent Purification System: Inert PureSolv MD5 SPS) and stored over 3 Å molecular sieves in ampoules equipped with J. Young's valves. Chlorobenzene and 1,2-difluorobenzene were dried over calcium hydride, distilled and stored over 3 Å molecular sieves. Deuterated solvents (CD_2Cl_2, $CDCl_3$, C_6D_6 and C_6D_5Br (99.6% D, Sigma Aldrich)) were dried and stored over 3 Å molecular sieves. All chemicals were, unless stated otherwise, purchased from commercial sources and used as received. The di-NTf_2-pyrazabole **1** was synthesised following reported literature procedure.[S1]

NMR spectra (^{1}H, $^{1}H\{^{11}B\}$, ^{11}B, $^{11}B\{^{1}H\}$, $^{13}C\{^{1}H\}$ and ^{19}F) were recorded on *Bruker Avance III 400 MHz, Bruker Avance III 500 MHz, Bruker Avance III 600 MHz* or *Bruker PRO 500 MHz* spectrometers. Chemical shifts (δ) are quoted in parts per million (ppm), coupling constants (*J*) are given in hertz (Hz) to the nearest 0.5 Hz, and as positive values regardless of their real individual signs. ^{1}H and ^{13}C shifts are referenced to the appropriate residual solvent peak while ^{11}B and ^{19}F shifts are referenced relative to external $BF_3{\cdot}Et_2O$ and C_6F_6, respectively. Abbreviations used are s (singlet), d (doublet), t (triplet), q (quartet), sep (septet), m (multiplet), br (broad). Background signals in ^{11}B NMR spectra arise to a significant degree from glass components of the probes used in our spectrometers. Unless otherwise stated, all NMR spectra were recorded at 20 °C.

Mass spectrometry was performed by the *Scottish Instrumentation and Resource Centre for Advanced Mass Spectrometry* (SIRCAMS) at the University of Edinburgh using electron impact (EI) or electrospray ionisation (ESI) techniques.

CHN Elemental Analyses were carried out by *Elemental Microanalysis Ltd*.

2 Comment on isomerism of pyrazabole derivatives

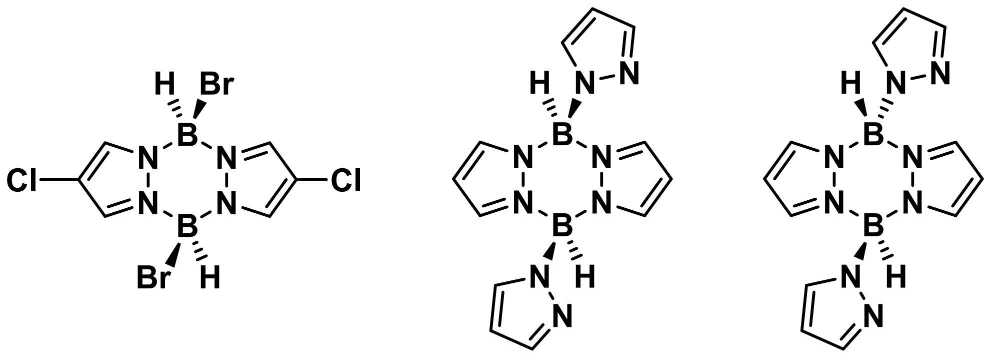

I **II** **III**

Figure S1: Pyrazabole derivatives reported by Nöth (**I**) and by Kresiński (**II** and **III**).

In 1983, Nöth and coworker published a series of pyrazabole derivatives, which included compound **I** (Figure S1, left).[S2] Compound **I** showed a single signal in the ^{11}B NMR (δ –7.4, d, $^{1}J_{BH}$ = 150 Hz), and therefore was assigned as a single isomer in solution. This work stated that only the *cis*-**I** was formed, this was based on the solid state structure obtained for **I** that only featured the *cis*-isomer. In subsequent work, *cis*/*trans* isomer mixtures for the lighter dihalo-pyrazabole congeners were observed by NMR spectroscopy from the groups of Trofimenko and Nöth.[S3, S4] However, no direct evidence for *cis*/*trans*-isomerism was observed by NMR spectroscopy in these previous series of dihalo-pyrazaboles.

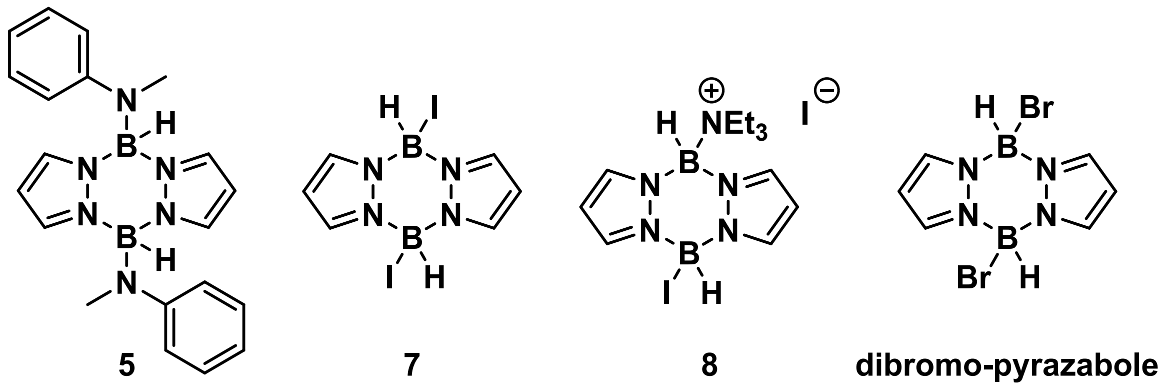

5 **7** **8** **dibromo-pyrazabole**

Figure S2: Pyrazabole derivatives isolated during our investigations.

In our case, the diiodo-pyrazabole **7**, the dibromo-pyrazabole and the mono-cation **8** all revealed two isomers *in situ* by NMR spectroscopy. Both **7** and **8** crystallized only as the *cis*-isomer, similarly to compound **I**. Based on Trofimenko and Nöth's previous work; we concluded that *cis*/*trans*-isomerization was happening in these compounds (possibly by dissociation of iodine, bromine or Et_3N). Therefore, we assign the 2 species visible *in situ*

by NMR spectroscopy as the *cis*- and *trans*-isomers. The feasibility of B–I cleavage (necessary for isomerization) was supported by **7** and pyrazabole forming the mono-iodo-pyrazabole **6**.

Notably, the di-N-methylaniline-pyrazabole **5** also showed isomerism by ^{1}H NMR spectroscopy, forming a species that slowly converted to a second species, we assign as an isomer. The latter was assigned to the solid state structure we obtained from **5** (boat conformer, *cis* configuration with both anilides on bowsprit position). *Cis*/*trans*-isomerism through dissociation of an anilide is unlikely to happen and this is further highlighted by the reported selective synthesis of **II** and **III** (Figure S1, centre and right),[S5] which are stable compounds that show no propensity to undergo *cis*/*trans*-isomerization (see References S6 and S7 for more examples of similar pyrazabole derivatives showing no isomerization).[S6, S7] This led us to tentatively conclude that the isomerism observed in **5** was due to conformational change and not *cis*/*trans*-isomerism.

3 Preparation of the pyrazabole derivatives, triflimide salt and substrate precursors

3.1 Preparation of diiodo-pyrazabole 7

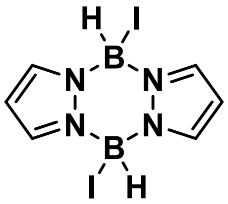

mixture of isomers A-7 and B-7

Iodine (1.59 g, 6.26 mmol, 1.00 equiv.) was dissolved in PhCl (50 mL) and added over 10 minutes to a solution of pyrazabole (1.00 g, 6.26 mmol, 1.00 equiv.) in PhCl (10 mL) at 0 °C in a J. Young's ampoule. After the addition was completed and no more gas evolution was observed, the solution was allowed to warm up to room temperature. The flask was sealed and stirred overnight, forming a white precipitate overtime. The white precipitate was isolated by filtration and washed with PhCl (3 x 5 mL). Drying the white residue *in vacuo* afforded a first crop of clean product **7** as a white solid (1.68 g). The PhCl filtered fractions were combined, concentrated *in vacuo*, and left in the freezer overnight. A pale yellow solid precipitated out from solution. Filtration and drying *in vacuo* afforded a second crop of **7** (0.27 g) as a pale yellow solid. Product **7** was obtained as a mixture of two isomers *A*-**7** and *B*-**7** (ratio 1.1:1) in an overall 75% yield (1.95 g, 4.70 mmol).

Note NMR data are for the mixture of both isomers of **7**.

^{1}H NMR (500 MHz, C_6D_5Br, 300 K) δ 7.67 (d, $^{3}J_{HH}$ = 2.5 Hz, NC*H*, 4H), 7.43 (d, $^{3}J_{HH}$ = 2.5 Hz, NC*H*, 4H), 5.99 (t, $^{3}J_{HH}$ = 2.5 Hz, CC*H*, 2H), 5.97 (t, $^{3}J_{HH}$ = 2.5 Hz, CC*H*, 2H), 6.00-4.80 (m br, BI*H*, 4H).

^{1}H {^{11}B} NMR (500 MHz, C_6D_5Br, 300 K) δ 7.67 (d, $^{3}J_{HH}$ = 2.5 Hz, NC*H*, 4H), 7.43 (d, 2.5 Hz, NC*H*, 4H), 5.99 (t, $^{3}J_{HH}$ = 2.5 Hz, CC*H*, 2H), 5.97 (t, $^{3}J_{HH}$ = 2.5 Hz, CC*H*, 2H), 5.57 (s br, BI*H*, 2H), 5.40 (s, br, BI*H*, 2H).

^{11}B NMR (160 MHz, C_6D_5Br, 300 K) δ –14.0 (d, $^{1}J_{BH}$ = 145 Hz, *B*IH), –15.7 (d, $^{1}J_{BH}$ = 151 Hz, *B*IH).

^{11}B {^{1}H} NMR (160 MHz, C_6D_5Br, 300 K) δ –14.0 (s, *B*IH), –15.7 (s, *B*IH).

^{13}C {^{1}H} NMR (126 MHz, C_6D_5Br, 300 K) δ 139.4 (s, N*C*H, 4C), 137.9 (s, N*C*H, 4C), 108.7 (s, C*C*H, 2C), 107.8 (s, C*C*H, 2C).

Mass Spectrum: HRMS (ESI+) m/z calculated for $C_6H_7N_4B_2I_2$: 410.89409; Found: 410.89235.

3.2 **Preparation of dibromo-pyrazabole**

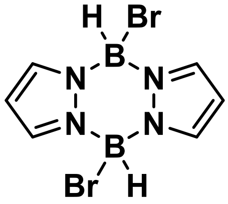

mixture of isomers
A-dibromopyrazabole
and B-dibromopyrazabole

A solution of BBr_3 (0.66 mL, 6.80 mmol, 0.68 equiv.) in DCM (17 mL) was added dropwise over the course of 15 minutes to a solution of pyrazabole (1.60 g, 10.00 mmol, 1.00 equiv.) in DCM (12 mL) at 0 °C in a J. Young's ampoule. After addition, the reaction was stirred at room temperature for 18 hours. Diborane gas generated during the reaction was quenched by bubbling the nitrogen outflow through degassed methanol. The white solid that formed overtime in the J. Young's ampoule was isolated by filtration, washed with hexane (2 x 10 mL) and dried *in vacuo* affording a mixture of isomers *A*- and *B*-dibromo-pyrazabole (ratio 1:1) as a white solid in 88% yield (2.28 g, 8.82 mmol).

Note NMR data are for the mixture of both isomers of dibromo-pyrazabole.

^{1}H NMR (500 MHz, $CDCl_3$, 300 K) δ 8.08 (d, $^{3}J_{HH}$ = 2.5 Hz, NC*H*, 4H), 7.99 (d, $^{3}J_{HH}$ = 2.5 Hz, NC*H*, 4H), 6.64 (t, $^{3}J_{HH}$ = 2.5 Hz, CC*H*, 2H), 6.59 (t, $^{3}J_{HH}$ = 2.5 Hz, CC*H*, 2H), 5.50-4.43 (m br, BBr*H*, 4H).

^{1}H {^{11}B} NMR (500 MHz, $CDCl_3$, 300 K) δ 8.08 (d, $^{3}J_{HH}$ = 2.5 Hz, NC*H*, 4H), 7.99 (d, $^{3}J_{HH}$ = 2.5 Hz, NC*H*, 4H), 6.64 (t, $^{3}J_{HH}$ = 2.5 Hz, CC*H*, 2H), 6.59 (t, $^{3}J_{HH}$ = 2.5 Hz, CC*H*, 2H), 5.05 (s br, BBr*H*, 2H), 4.90 (s br, BBr*H*, 2H).

^{11}B NMR (160 MHz, $CDCl_3$, 300 K) δ –5.7 (d, $^{1}J_{BH}$ = 143 Hz, *B*BrH), –6.7 (d, $^{1}J_{BH}$ = 121 Hz, *B*BrH).

^{11}B {^{1}H} NMR (160 MHz, $CDCl_3$, 300 K) δ –5.7 (s, *B*BrH), –6.7 (s, *B*BrH).

^{13}C {^{1}H} NMR (126 MHz, $CDCl_3$, 300 K) δ 138.7 (s, N*C*H, 4C), 138.6 (s, N*C*H, 4C), 108.8 (s, C*C*H, 2C), 108.0 (s, C*C*H, 2C).

Mass Spectrum: dibromo-pyrazabole was not observed by mass spectrometry analysis in our hands (ESI and EI).

Elemental Analysis: Calculated for $C_6H_8B_2Br_2N_4$: C 22.69%, H 2.54%, N 17.64%; Observed: C 22.50%, H 2.40%, N 16.71%.

3.3 **Preparation of di-N-methylaniline-pyrazabole 5**

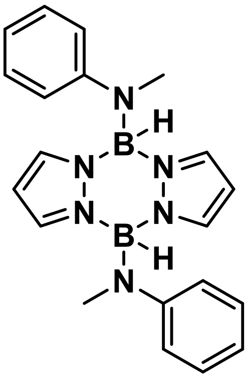

mixture of isomers A-5 and B-5

Di-NTf_2-pyrazabole **1** (0.40 g, 0.56 mmol, 1.00 equiv.) was dissolved in DCM (5 mL) in a J. Young's ampoule. N-methylaniline (0.12 mL, 1.11 mmol, 2.00 equiv.) and Et_3N (0.16 mL, 1.11 mmol, 2.00 equiv.) were successively added at room temperature and the resulting solution was stirred for 10 minutes. Volatiles were removed *in vacuo*. The residue was extracted with pentane (3 x 5 mL). Concentrating the pentane solution *in vacuo* to a third of its initial volume (approx. 5 mL) resulted in a white solid precipitating. The flask was stored in a fridge for 48 hours, during which more solid precipitated out of solution. The precipitate was isolated by filtration and dried under vacuum, affording a mixture of isomers *A*- and *B*-**5** as a white solid in 52% yield (0.11 g, 0.29 mmol).

*Almost complete isomerisation of A-**5** to B-**5** is observed overtime in solution. B-**5** was assigned from the solid state structure obtained from **5** as the boat conformer, cis configuration with both anilides on bowsprit position.*

A-**5**:

^{1}H NMR (500 MHz, CD_2Cl_2, 300 K) δ 7.72 (d, $^3J_{HH}$ = 2.4 Hz, NC*H*, 4H), 7.19-7.12 (m, ^{meta}C*H*, 4H), 6.97-6.91 (m, ^{ortho}C*H*, 4H), 6.67-6.62 (m, ^{para}C*H*, 2H), 6.48 (t, $^3J_{HH}$ = 2.4 Hz, CC*H*, 2H), 5.20-4.14 (m br, B*H*, 2H), 2.69 (s, NC*H*$_3$, 6H).

^{1}H {^{11}B} NMR (500 MHz, CD_2Cl_2, 300 K) δ 7.72 (d, $^3J_{HH}$ = 2.4 Hz, NC*H*, 4H), 7.19-7.12 (m, ^{meta}C*H*, 4H), 6.97-6.91 (m, ^{ortho}C*H*, 4H), 6.67-6.62 (m, ^{para}C*H*, 2H), 6.48 (t, $^3J_{HH}$ = 2.4 Hz, CC*H*, 2H), 4.67 (s br, B*H*, 2H), 2.69 (s, NC*H*$_3$, 6H).

^{11}B NMR (160 MHz, CD_2Cl_2, 300 K) δ –1.7 (d, $^1J_{BH}$ = 120 Hz, *B*HN, 2B).

^{11}B {^{1}H} NMR (160 MHz, CD_2Cl_2, 300 K) δ –1.7 (s, *B*HN, 2B).

^{13}C {^{1}H} NMR (126 MHz, CD_2Cl_2, 300 K) δ 152.4 (s, ^{ipso}CN, 2C), 136.3 (s, N*C*H, 4C), 129.1 (s, ^{meta}*C*H, 4C), 116.5 (s, ^{para}*C*H, 2C), 115.1 (s, ^{ortho}*C*H, 4C), 107.5 (s, C*C*H, 2C), 35.0 (s, N*C*H_3, 2C).

B-**5**:

^{1}H NMR (600 MHz, CD_2Cl_2, 300 K) δ 7.60 (s br, NC*H*, 4H), 7.19-7.07 (m br, ^{meta}C*H*, 4H), 7.01-6.70 (m br, ^{ortho}C*H*, 4H), 6.66 (t, br, $^{3}J_{HH}$ = 7.1 Hz, ^{para}C*H*, 2H), 6.40 (t br, CC*H*, 2H), 4.90-3.84 (m br, B*H*, 2H), 2.96 (s br, NCH_3, 6H).

^{1}H {^{11}B} NMR (500 MHz, CD_2Cl_2, 300 K) δ 7.60 (s br, NC*H*, 4H), 7.19-7.07 (m br, ^{meta}C*H*, 4H), 7.01-6.70 (m br, ^{ortho}C*H*, 4H), 6.66 (t, br, $^{3}J_{HH}$ = 7.1 Hz, ^{para}C*H*, 2H), 6.40 (t br, CC*H*, 2H), 4.41 (s br, B*H*, 2H), 2.96 (s br, NCH_3, 6H).

^{11}B NMR (160 MHz, CD_2Cl_2, 300 K) δ –1.7 (d, $^{1}J_{BH}$ = 120 Hz, *B*HN, 2B).

^{11}B {^{1}H} NMR (160 MHz, CD_2Cl_2, 300 K) δ –1.7 (s, *B*HN, 2B).

^{13}C {^{1}H} NMR* (126 MHz, **C_6D_6, 343 K**) δ 151.8 (s br, ^{ipso}CN, 2C), 135.0 (s, N*C*H, 4C), 129.2 (s, ^{meta}*C*H, 4C), 117.3 (s, ^{para}*C*H, 2C), 115.6 (s, ^{ortho}*C*H, 4C), 106.5 (s, C*C*H, 2C), 36.7 (s br, N*C*H_3, 2C).

*A VT NMR experiment in C_6D_6 (343 K) was necessary to allow observation of all the ^{13}C NMR signals of the *B*-**5** isomer as very broad resonances were observed at room temperature due to a fluxional process.

Mass Spectrum: HRMS (ESI+) m/z calculated for $C_{20}H_{24}B_2N_6$: 370.22431; Found: 370.22274.

3.4 Preparation of mono-cation 8

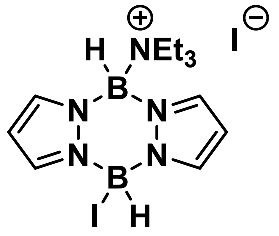

mixture of isomers A-8 and B-8

A solution of diiodo-pyrazabole **7** (50 mg, 0.12 mmol, 1.00 equiv.) in 1,2-difluorobenzene (12.5 mL) in a J. Young's ampoule was heated to 45 °C until all the solid was dissolved. The solution was cooled down to room temperature and Et_3N (17 µL, 0.12 mmol, 1.00 equiv.) was added dropwise to the solution. After stirring for 5 minutes, volatiles were removed *in vacuo*. The pale yellow residue was washed with 1,2-difluorobenzene (2 x 2 mL) affording a mixture of *A*- and *B*-**8** isomers (evidenced by the presence of multiple signals in the ^{11}B NMR) as a white solid in 39% yield (24 mg, 0.05 mmol).

Crystals of **8** suitable for X-Ray diffraction analysis were obtained from a different reaction described below. In a J. Young's ampoule, a solution of diiodo-pyrazabole **7** (0.10 g, 0.24 mmol, 1.00 equiv.) in 1,2-difluorobenzene (25 mL) was heated to 45 °C until all the solid was dissolved. The solution was cooled down to room temperature and Et_3N (34 µL, 0.24 mmol, 1.00 equiv.) was added dropwise to the solution. After stirring for 5 minutes, the solution was concentrated until solid started to precipitate. The solution was isolated by filtration. Successive fractional crystallizations from the filtrate at low temperature (–20 °C) over a week afforded colourless plate-shaped crystals of **8**.

The extremely poor solubility of **8** in polar, weakly-coordinating solvents resulted in poor NMR data. Compound **8** was unstable in THF-d8, CD_2Cl_2 or MeCN-d3. The extremely poor solubility of **8** in C_6D_5Br or $CDCl_3$ resulted in very weak and broad ^{1}H NMR signals, not suitable for characterisation. 1,2-difluorobenzene allowed us to record ^{11}B NMR data of **8**, no ^{1}H NMR assignment could be done due to the non-deuterated aromatic solvent peaks covering signals from **8**.

^{11}B NMR (160 MHz, $C_6H_4F_2$, 300 K) δ –2.7 (d, $^1J_{BH}$ = 104 Hz, *B*HNEt$_3$, 2B), –13.9 (d, $^1J_{BH}$ = 139 Hz, *B*IH, 1B), –15.4 (d, $^1J_{BH}$ = 153 Hz, *B*IH, 1B).

^{11}B {^{1}H} NMR (160 MHz, $C_6H_4F_2$, 300 K) δ –2.7 (br s, *B*HNEt$_3$, 2B), –13.9 (s, *B*IH, 1B), –15.5 (s, *B*IH, 1B).

Mass Spectrum: Compound **8** was unstable to mass spectrum analysis and the only the cationic fragment without Et_3N was observed.

Elemental Analysis: Calculated for $C_{12}H_{23}B_2I_2N_5$: C 28.11%, H 4.52%, N 13.66%; Observed: C 29.17%, H 4.45%, N 12.89%. Note this is the closest CHN data we could obtain, therefore it is possible that 8 contains minor impurities that are not visible in the ^{11}B NMR spectrum.

3.5 Preparation of $[Et_3NH]NTf_2$

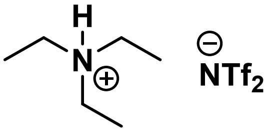

Preparation 1:

A solution of $HNTf_2$ (0.28 g, 1.00 mmol, 1.00 equiv.) in DCM (3 mL) was added to a solution of Et_3N (0.10 g, 1.00 mmol, 1.00 equiv.) in DCM (5 mL) at room temperature in a J. Young's ampoule. The solution was stirred for 0.5 hour. Removal of the volatiles *in vacuo* afforded the product $[Et_3NH]NTf_2$ as a colourless oil in quantitative yield (0.38 g, 1.00 mmol). Analytical data were in accordance with literature values.[S8]

Preparation 2:

$[Et_3NH]Cl$ (0.48 g, 3.48 mmol, 1.00 equiv.) and $LiNTf_2$ (1.00 g, 3.48 mmol, 1.00 equiv.) were dissolved in DCM (10 mL) at room temperature in a J. Young's ampoule. The solution was stirred for 0.5 hour and filtered to remove the formed LiCl. Volatiles were removed *in vacuo*, affording the product $[Et_3NH]NTf_2$ as a colourless oil in 86% yield (1.15 g, 3.01 mmol). Analytical data were in accordance with literature values.[S8]

^{1}H NMR (500 MHz, $CDCl_3$, 300 K) δ 7.22 (s br, N*H*, 1H), 3.21 (q, $^{3}J_{HH}$ = 7.3 Hz, CH_2, 6H), 1.36 (t, $^{3}J_{HH}$ = 7.3 Hz, CH_3, 9H).

^{13}C {^{1}H} NMR (126 MHz, $CDCl_3$, 300 K) δ 119.8 (q, $^{1}J_{CF}$ = 321.2 Hz, *C*F_3, 2C), 47.3 (s, *C*H_2, 3C), 8.7 (s, *C*H_3, 3C).

^{19}F NMR (471 MHz, $CDCl_3$, 300K) δ –78.8 (s, CF_3, 6F).

3.6 **Preparation of 4-piperidine-N-methylaniline**

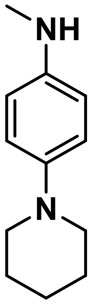

Modified procedure from literature.[S9]

In a J. Young's ampoule, a solution of 4-piperidine-aniline (1.50 g, 8.51 mmol, 1.00 equiv.) in DMF (25 mL) was added slowly at 0 °C to K_2CO_3 (1.53 g, 11.06 mmol, 1.30 equiv.) and stirred at this temperature for 15 min. CH_3I (0.64 mL, 10.21 mmol, 1.20 equiv.) was added dropwise at 0 °C. The ampoule was sealed and heated at 55 °C for 24 hours. The solution was cooled down to room temperature and a NaOH (1M) water solution (50 mL) was added. The product was extracted with DCM (3 x 15 mL). The combined organic layers were washed with brine (2 x 20 mL), dried with Na_2SO_4, filtered and volatiles were removed *in vacuo*. Purification by chromatographic column (gradient: 0-30% ethyl acetate in petroleum ether) afforded the product 4-piperidine-N-methylaniline as a red oil in 16% yield (0.26 g, 1.37 mmol).

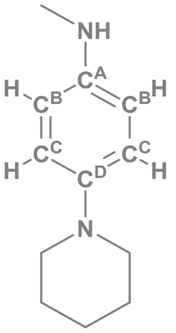

Figure S3: Scheme of 4-piperidine-N-methylaniline.

^{1}H NMR (500 MHz, $CDCl_3$, 300 K) δ 6.88 (d, $^3J_{HH}$ = 8.3 Hz, C^CH, 2H), 6.59 (d, $^3J_{HH}$ = 8.3 Hz, C^BH, 2H), 3.43 (s br, N*H*, 1H), 3.03-2.92 (m, NCH_2, 4H), 2.81 (s, NCH_3, 3H), 1.76-1.68 (m, NCH_2CH_2, 4H), 1.57-1.49 (m, $NCH_2CH_2CH_2$, 2H).

^{13}C {^{1}H} NMR (126 MHz, $CDCl_3$, 300 K) δ 145.1 (s, C^DN, 1C), 143.8 (s, C^A, 1C), 119.5 (s, C^CH, 2C), 113.5 (s, C^BH, 2C), 53.0 (s, N*C*H_2, 2C), 31.6 (s, N*C*H_3, 1C), 26.4 (s, NCH_2*C*H_2, 2C), 24.4 (s, NCH_2CH_2*C*H_2, 1C).

Mass Spectrum: HRMS (ESI+) m/z calculated for $C_{12}H_{19}N_2$: 191.1543; Found: 191.1549.

3.7 NMR spectra of isolated and novel precursors

3.7.1 NMR spectra of diiodo-pyrazabole 7

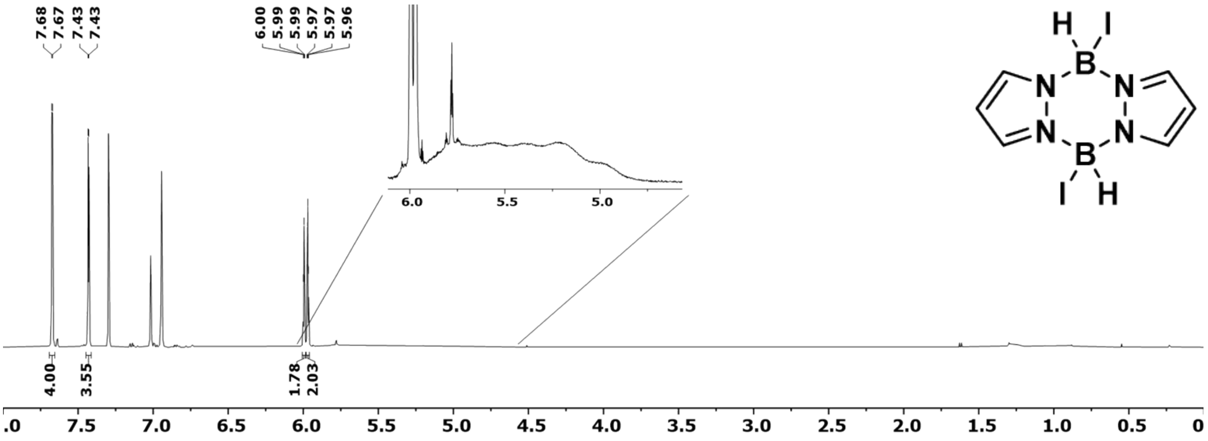

Figure S4: ^{1}H NMR spectrum (500 MHz, C_6D_5Br, 300 K) of diiodo-pyrazabole **7**.

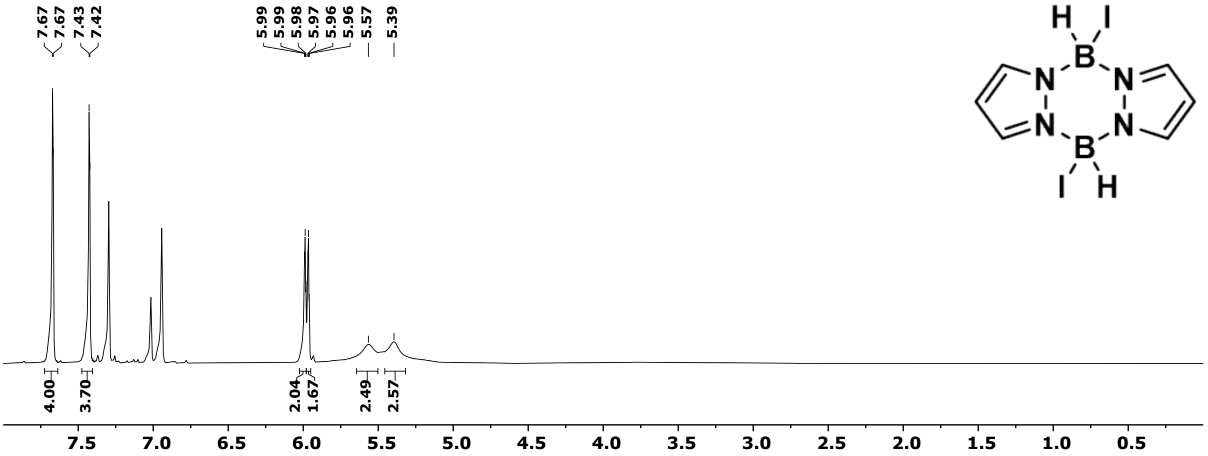

Figure S5: $^{1}H\ \{^{11}B\}$ NMR spectrum (500 MHz, C_6D_5Br, 300 K) of diiodo-pyrazabole **7**.

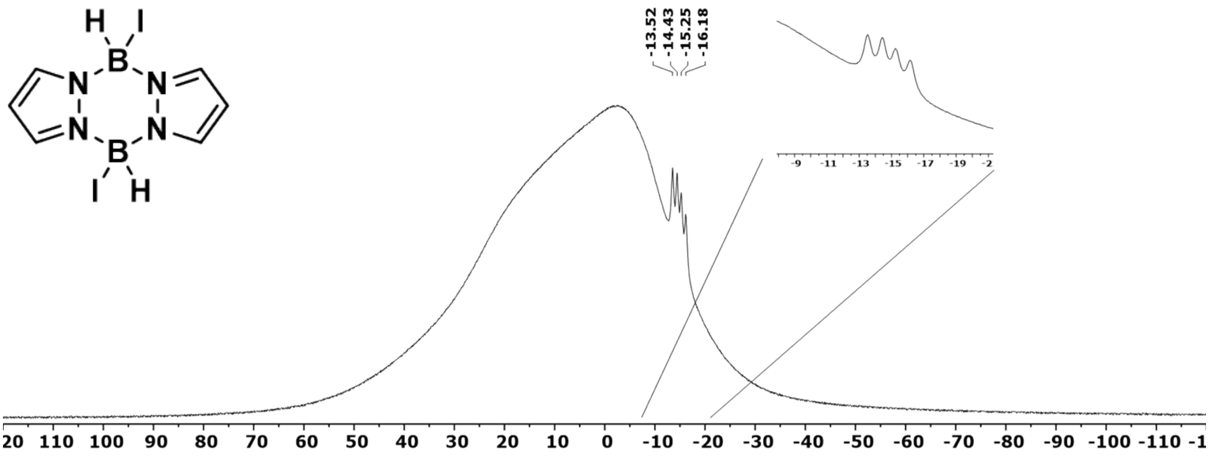

Figure S6: ^{11}B NMR spectrum (160 MHz, C_6D_5Br, 300 K) of diiodo-pyrazabole **7**.

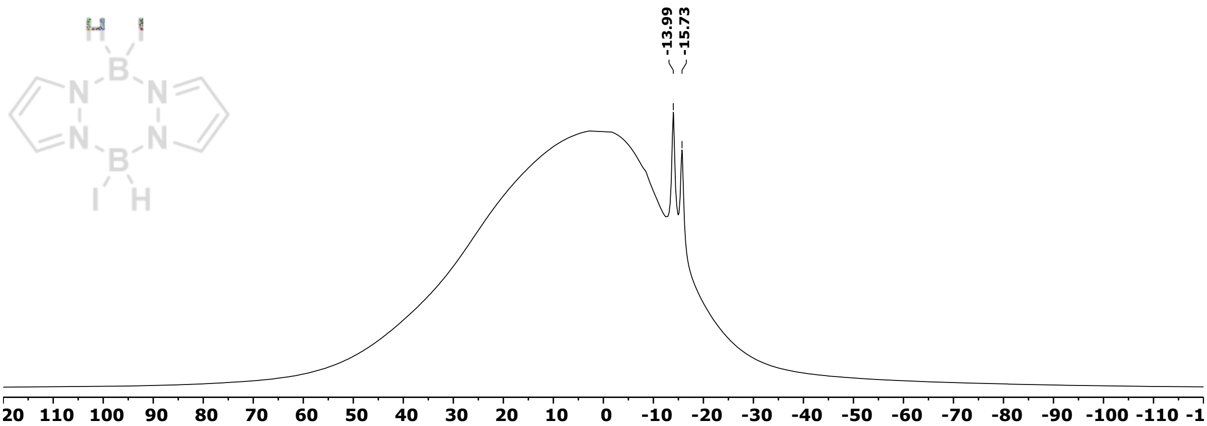

Figure S7: ^{11}B {^{1}H} NMR spectrum (160 MHz, C_6D_5Br, 300 K) of diiodo-pyrazabole **7**.

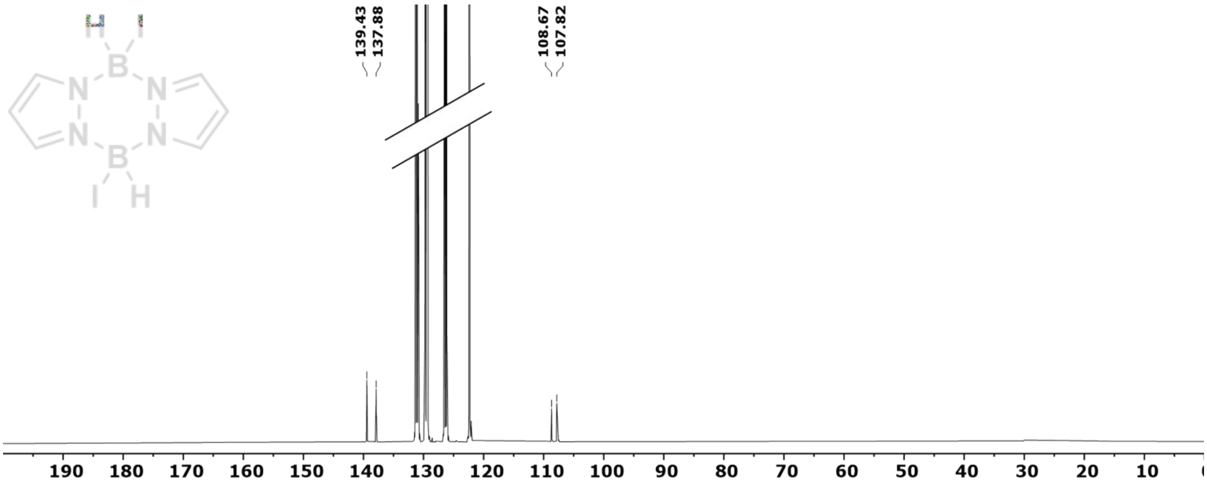

Figure S8: ^{13}C {^{1}H} NMR spectrum (126 MHz, C_6D_5Br, 300 K) of diiodo-pyrazabole **7**.

3.7.2 NMR spectra of dibromo-pyrazabole

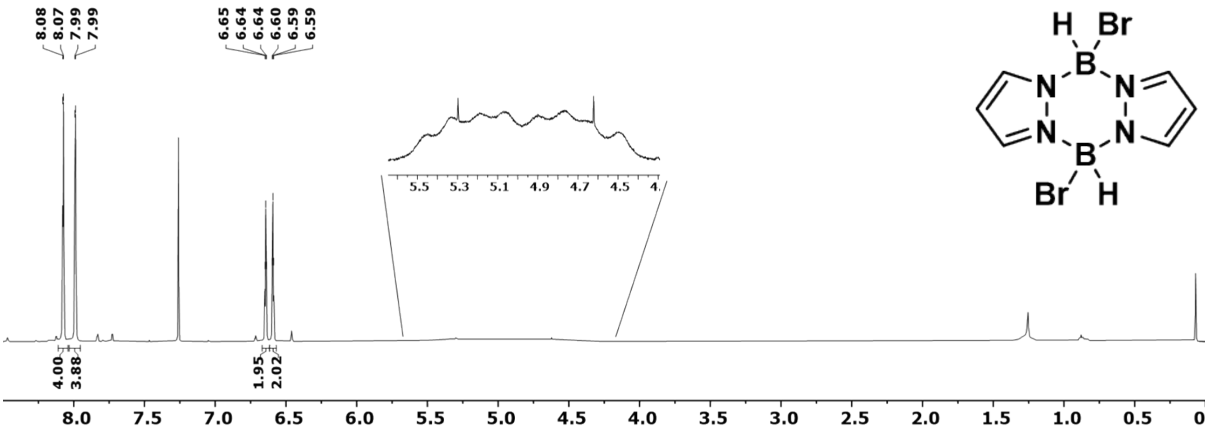

Figure S9: ^{1}H NMR spectrum (500 MHz, $CDCl_3$, 300 K) of dibromo-pyrazabole.

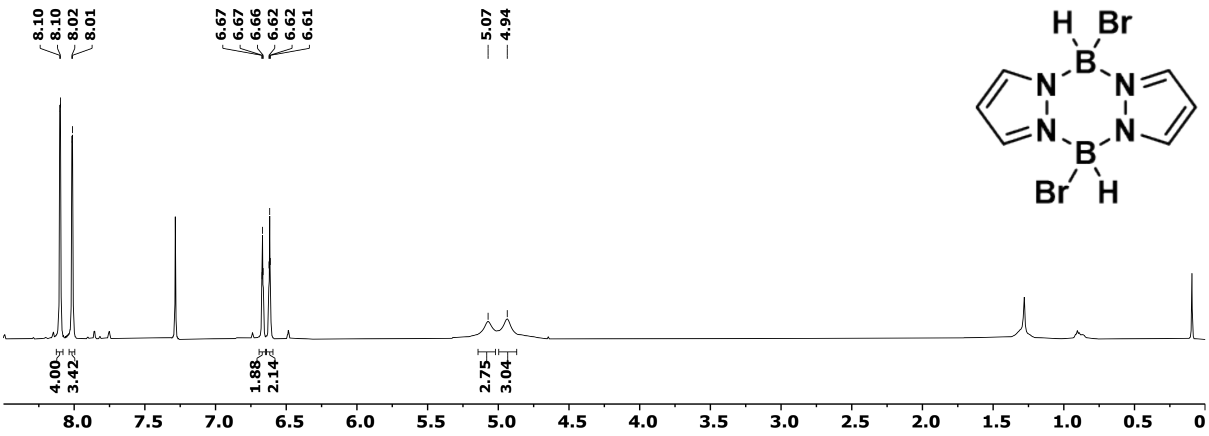

Figure S10: ^{1}H $\{^{11}B\}$ NMR spectrum (500 MHz, $CDCl_3$, 300 K) of dibromo-pyrazabole.

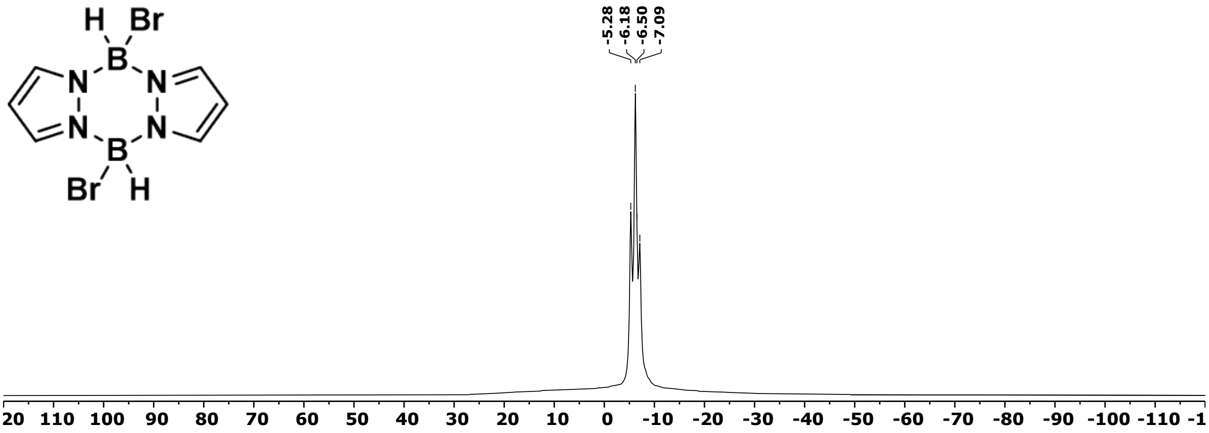

Figure S11: ^{11}B NMR spectrum (160 MHz, $CDCl_3$, 300 K) of dibromo-pyrazabole.

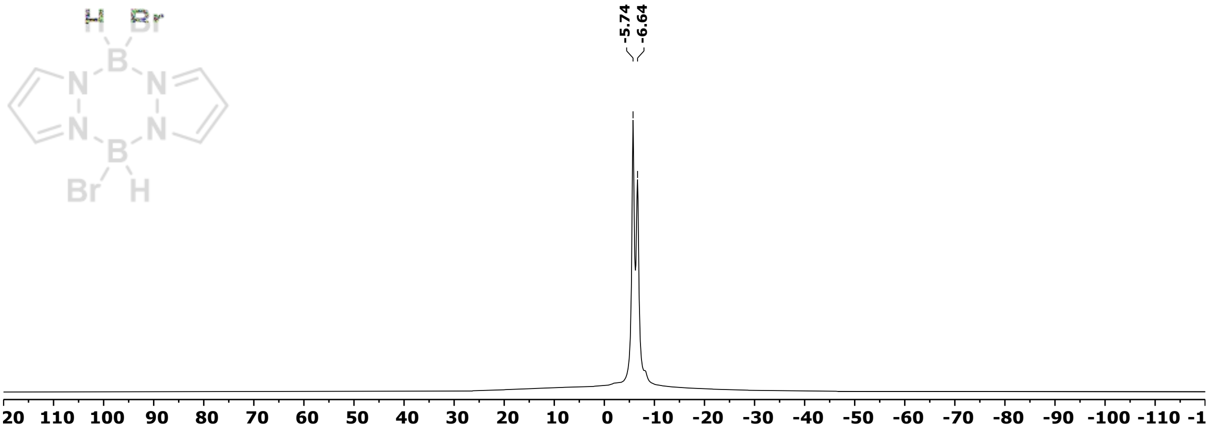

Figure S12: $^{11}B\{^1H\}$ NMR spectrum (160 MHz, $CDCl_3$, 300 K) of dibromo-pyrazabole.

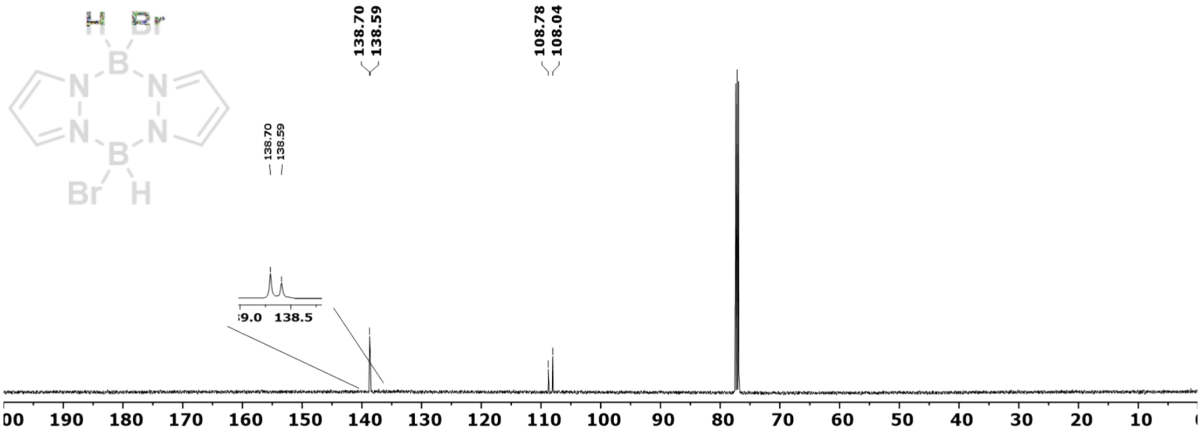

Figure S13: $^{13}C\{^1H\}$ NMR spectrum (126 MHz, $CDCl_3$, 300 K) of dibromo-pyrazabole.

3.7.3 NMR spectra of di-N-methylaniline-pyrazabole 5

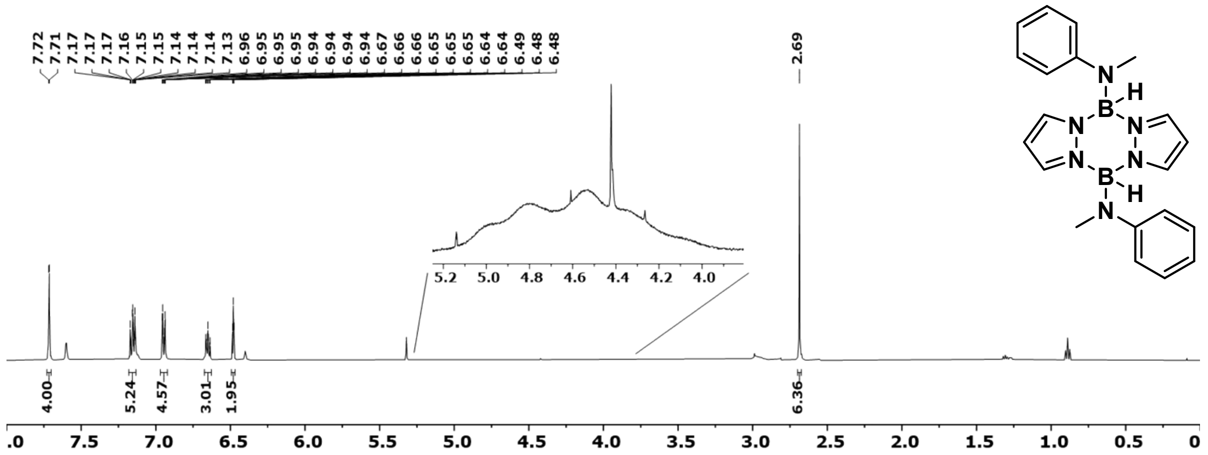

Figure S14: ^{1}H NMR spectrum (500 MHz, CD_2Cl_2, 300K) of the di-N-methylaniline-pyrazabole *A*- and *B*-**5**. This spectrum was recorded just after the synthesis and isolation of **5** with the *A*-isomer being the major species. Only the signals from *A*-**5** are labelled on this spectrum (the signal from *B*-**5** can be found on Figure S15).

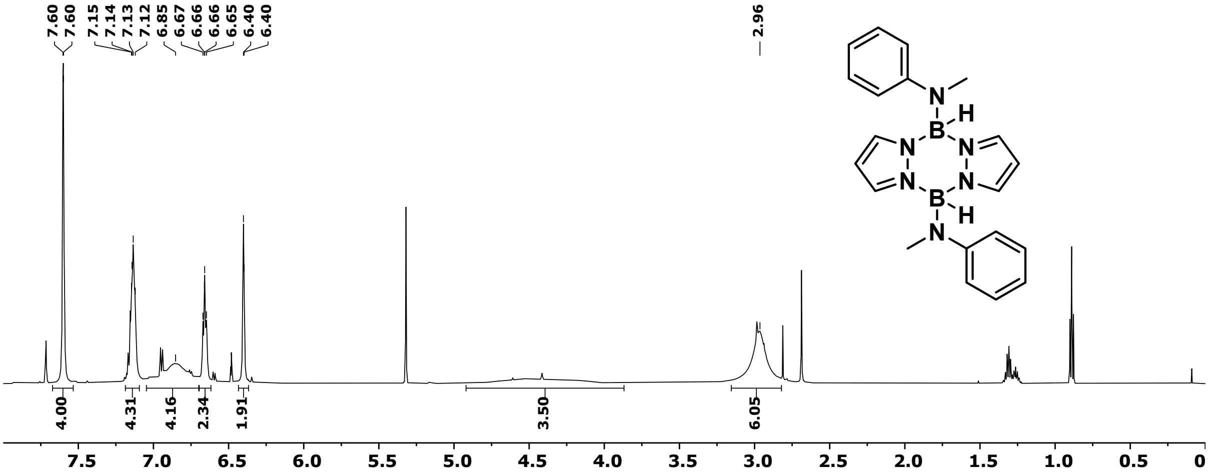

Figure S15: ^{1}H NMR spectrum (600 MHz, CD_2Cl_2, 300K) of the di-N-methylaniline-pyrazabole *A*- and *B*-**5**. This spectrum was recorded after leaving **5** in solution overnight. Almost complete isomerisation to *B*-**5** is observed overtime in solution. Only the signals from *B*-**5** are labelled on this spectrum (the signal from *A*-**5** can be found on Figure S14).

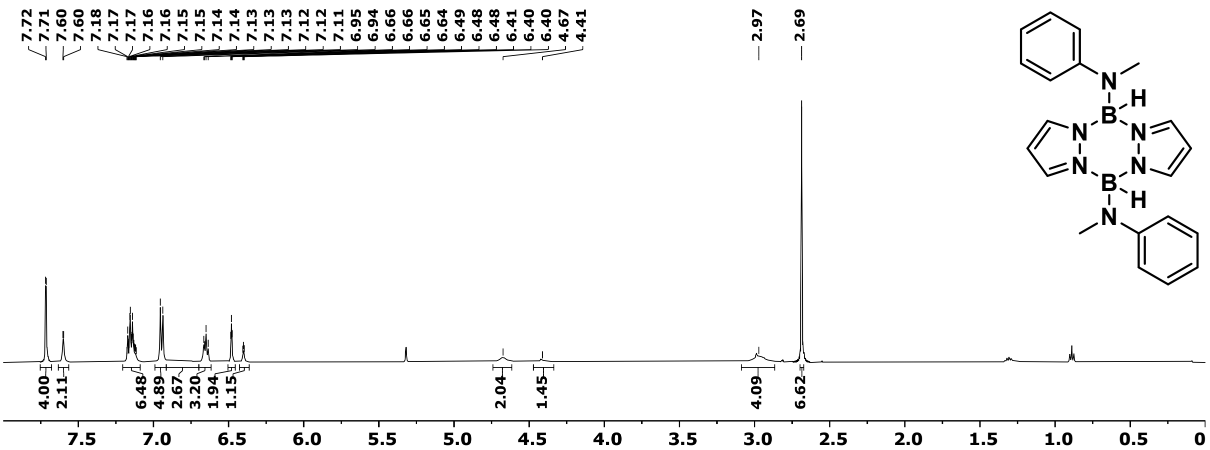

Figure S16: $^{1}H\{^{11}B\}$ NMR spectrum (500 MHz, CD_2Cl_2, 300K) of the di-N-methylaniline-pyrazabole *A*- and *B*-**5** isomers.

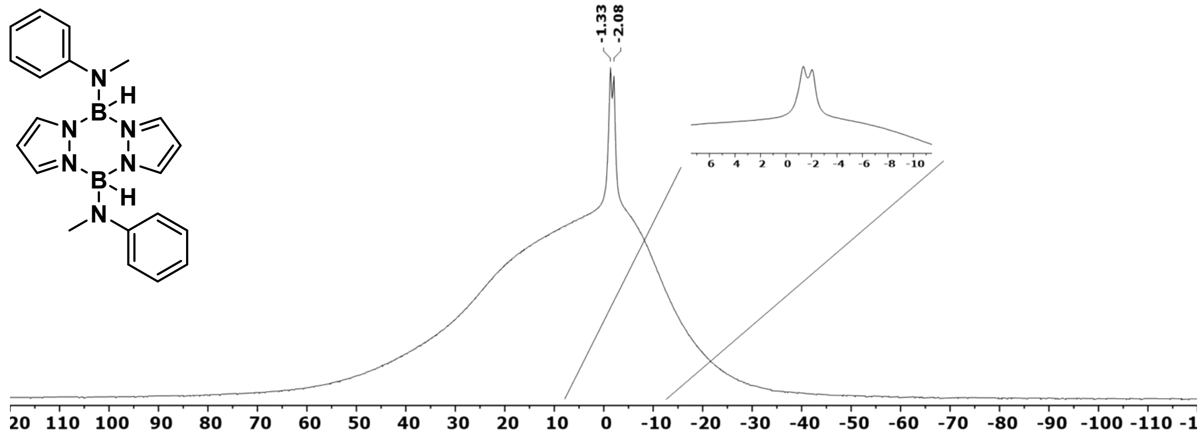

Figure S17: ^{11}B NMR spectrum (160 MHz, CD_2Cl_2, 300 K) of the di-N-methylaniline-pyrazabole *A*- and *B*-**5** isomers.

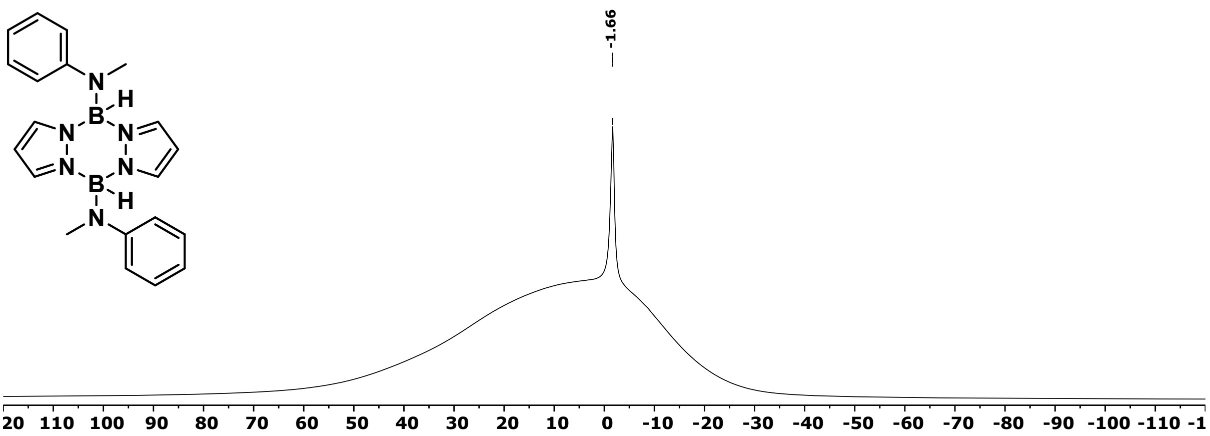

Figure S18: $^{11}B\{^{1}H\}$ NMR spectrum (160 MHz, CD_2Cl_2, 300 K) of the di-N-methylaniline-pyrazabole *A*- and *B*-**5** isomers.

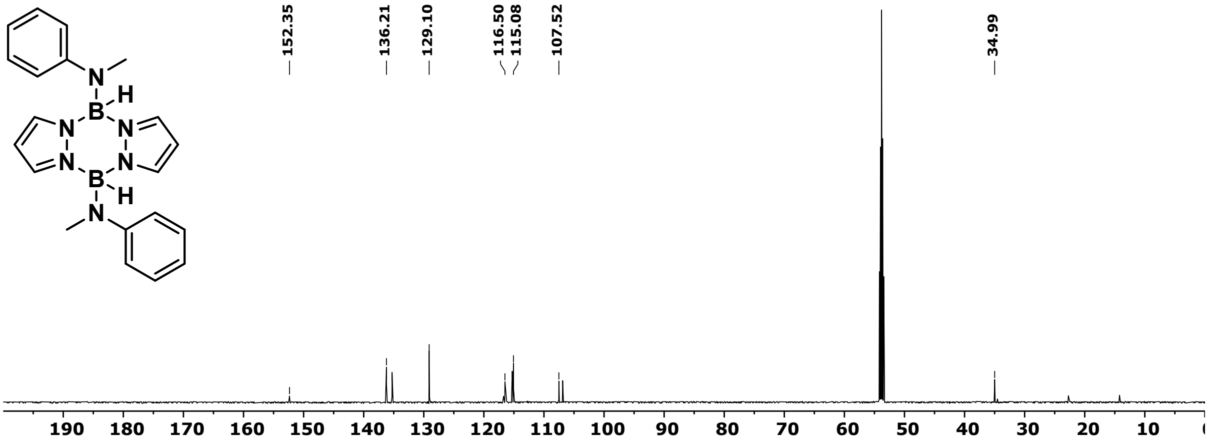

Figure S19: $^{13}C\{^{1}H\}$ NMR spectrum (126 MHz, CD_2Cl_2, 300 K) of the di-N-methylaniline-pyrazabole *A*- and *B*-**5**. This spectrum was recorded just after the synthesis and isolation of **5** with the *A*-isomer being formed as the major species. Only the signals from *A*-**5** are labelled on this spectrum (the signal from *B*-**5** in C_6D_6 at 343 K can be found on Figure S20).

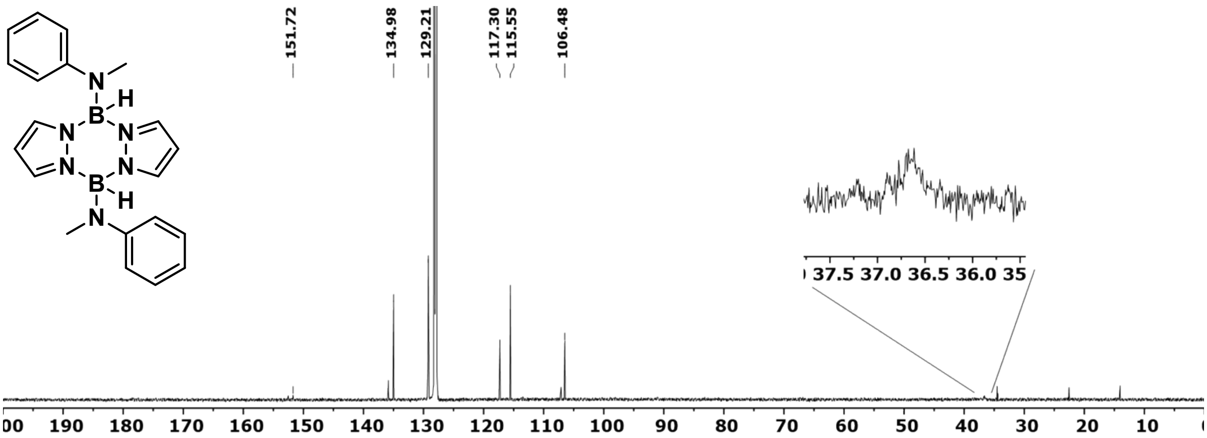

Figure S20: $^{13}C\{^{1}H\}$ NMR spectrum (126 MHz, C_6D_6, 343 K) of the di-N-methylaniline-pyrazabole *A*- and *B*-**5**. VT NMR experiment at 343 K was necessary to allow observation of all the ^{13}C NMR signals of the *B*-**5**. Only the signals from *B*-**5** are labelled on this spectrum (the signal from *A*-**5** in CD_2Cl_2 at 300 K can be found on Figure S19).

3.7.4 NMR spectra of mono-cation 8

The extremely poor solubility of **8** in polar, weakly-coordinating solvents resulted in poor NMR data. Compound **8** was unstable in THF-d8, CD_2Cl_2 or MeCN-d3. The extremely poor solubility of **8** in C_6D_5Br or $CDCl_3$ resulted to very weak and broad ^1H NMR signals, not suitable for characterisation. 1,2-difluorobenzene allowed us to record ^{11}B NMR data of **8**, no ^1H NMR assignment could be done due to the non-deuterated aromatic solvent peaks covering signals from **8**.

Note the two doublets at ca. –15 ppm do not correspond to the diiodo-pyrazabole **7** precursor as these resonate at δ –13.8 (d), –15.4 (d) ppm in this solvent and have a larger coupling constant than that observed in 8.

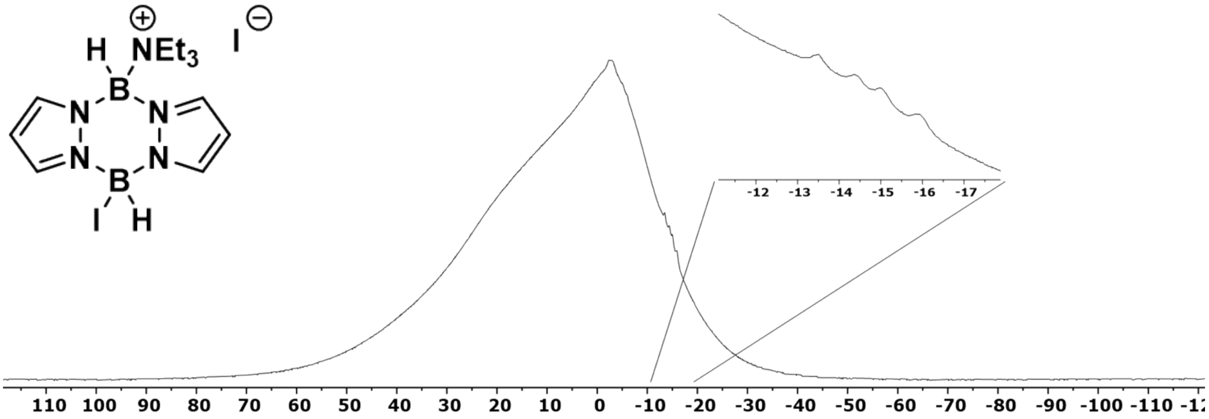

Figure S21: ^{11}B NMR spectrum (160 MHz, $C_6H_4F_2$, 300 K) of the mono-cation *A*- and *B*-**8** isomers.

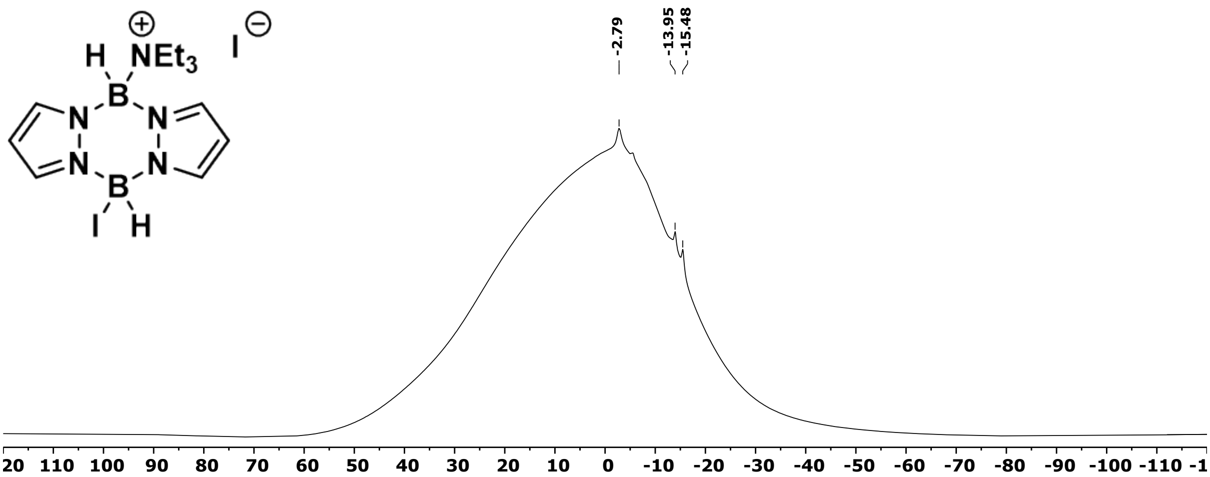

Figure S22: ^{11}B {^1H} NMR spectrum (160 MHz, $C_6H_4F_2$, 300 K) of the mono-cation *A*- and *B*-**8** isomers.

3.7.5 NMR spectra of 4-piperidine-N-methylaniline

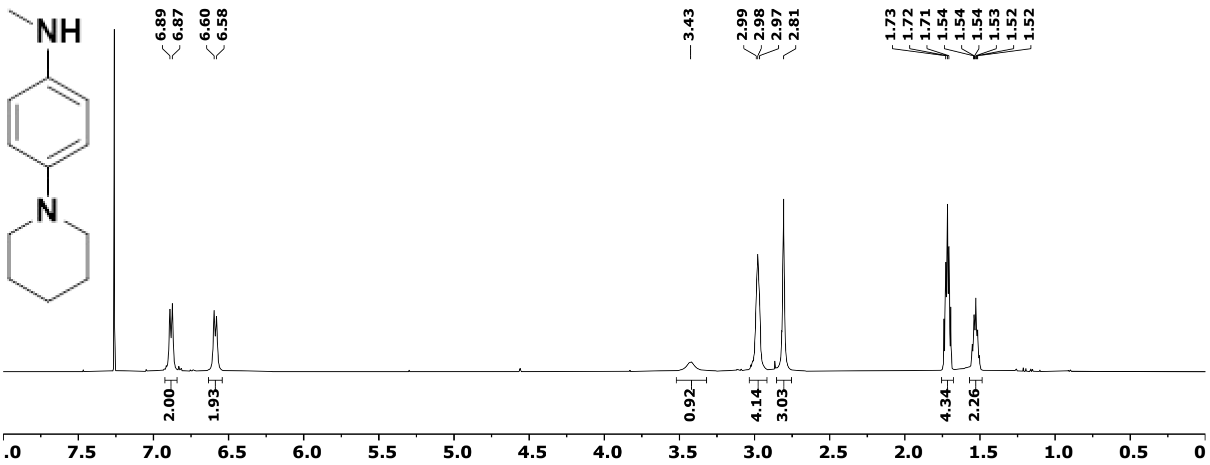

Figure S23: ^{1}H NMR spectrum (500 MHz, $CDCl_3$, 300 K) of 4-piperidine-N-methylaniline.

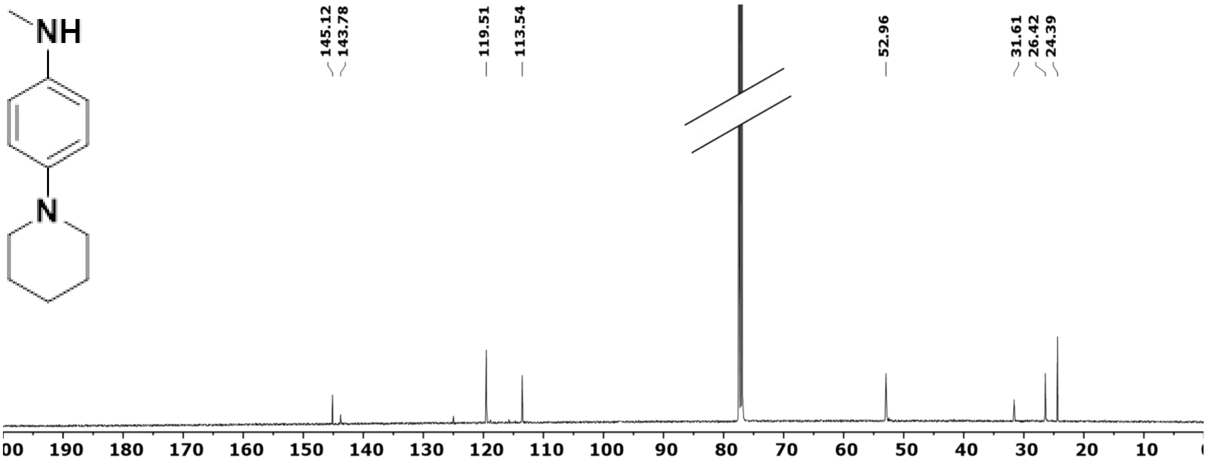

Figure S24: ^{13}C {^{1}H} NMR spectrum (126 MHz, $CDCl_3$, 300 K) of 4-piperidine-N-methylaniline.

4 Preliminary studies

4.1 BDB with di-NTf_2-pyrazabole 1

4.1.1 Base scope in BDB with di-NTf_2-pyrazabole 1

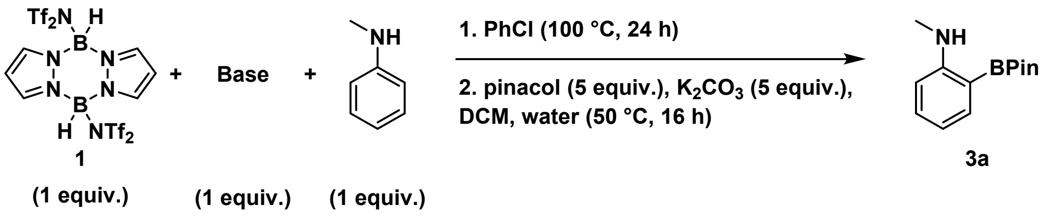

Di-NTf_2-pyrazabole **1** (0.18 g, 0.25 mmol, 1.00 equiv.), base (0.25 mmol, 1.00 equiv.) and N-methylaniline (27 µL, 0.25 mmol, 1.00 equiv.) were combined in PhCl (1.5-2 mL) in a J. Young's ampoule and heated at 100 °C for 24 hours. The solution was cooled down to room temperature. Pinacol (0.15 g, 1.25 mmol, 5.00 equiv.), K_2CO_3 (0.17 g, 1.25 mmol, 5.00 equiv.), DCM (2.3 mL) and water (2.3 mL) were sequentially added. The resulting biphasic mixture was heated at 50 °C for 16 hours with vigorous stirring. The organic phase was extracted with pentane (4 x 10 mL). The combined organic phases were washed with water (4 x 10 mL), dried over $MgSO_4$, and filtered. Concentration *in vacuo* afforded the desired product **3a**.

Entry	Solvent	Temperature	Base	Yield
1	PhCl	100 °C	2,6-di-*tert*-butyl-4-methylpyridine	81%[a]
2	PhCl	100 °C	Et_3N	65%
3	PhCl	100 °C	DIPEA	62%

Table S1: Base screening for BDB reaction with di-NTf_2-pyrazabole **1**.

[a]Product was obtained with remaining 2,6-di-*tert*-butyl-4-methylpyridine, whereas with the other two bases **3a** was afforded clean after this work-up.

4.1.2 Characterisation of [2]NTf₂

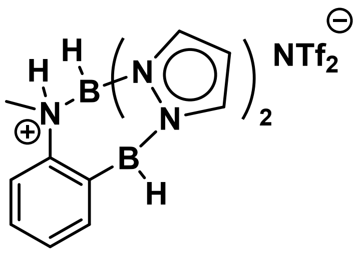

Di-NTf_2-pyrazabole **1** (8 mg, 0.01 mmol, 1.00 equiv.) and di-N-methylaniline-pyrazabole **5** (4 mg, 0.01 mmol, 1.00 equiv.) were dissolved in $CDCl_3$ (0.4 mL) and heated at 60 °C for 2.5 days in a J. Young's NMR tube. NMR analysis showed conversion to a single major new product which was identified as **[2]NTf$_2$** (yield was not recorded).

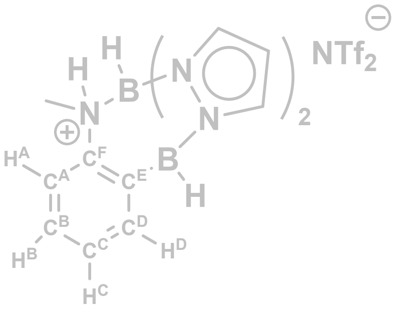

Figure S25: Scheme of **[2]NTf$_2$**.

^{1}H NMR (500 MHz, $CDCl_3$, 300 K) δ 8.10 (d, $^3J_{HH}$ = 2.6 Hz, NC*H*, 1H), 8.05 (s br, N*H*, 1H), 8.01 (d, $^3J_{HH}$ = 2.5 Hz, NC*H*, 1H), 7.99 (d, $^3J_{HH}$ = 2.4 Hz, NC*H*, 1H), 7.75 (d, $^3J_{HH}$ = 2.3 Hz, NC*H*, 1H), 7.61 (dd, $^3J_{HH}$ = 7.3 Hz, $^4J_{HH}$ = 1.8 Hz, H^D, 1H), 7.40 (dd, $^3J_{HH}$ = 8.1 Hz, $^4J_{HH}$ = 1.3 Hz, H^A, 1H), 7.34 (td, $^3J_{HH}$ = 7.7 Hz, $^4J_{HH}$ = 1.8 Hz, H^B, 1H), 7.28 (td, $^3J_{HH}$ = 7.3 Hz, $^4J_{HH}$ = 1.2 Hz, H^C, 1H), 6.60 (t, $^3J_{HH}$ = 2.4 Hz, CC*H*, 1H), 6.40 (t, $^3J_{HH}$ = 2.5 Hz, CC*H*, 1H), 4.90-3.59 (m br, B*H*, 2H), 2.84 (d, $^3J_{HH}$ = 5.5 Hz, NCH_3, 3H).*

^{1}H {^{11}B} NMR (500 MHz, $CDCl_3$, 300 K) δ 8.10 (d, $^3J_{HH}$ = 2.6 Hz, NC*H*, 1H), 8.05 (s br, N*H*, 1H), 8.01 (d, $^3J_{HH}$ = 2.5 Hz, NC*H*, 1H), 7.99 (d, $^3J_{HH}$ = 2.4 Hz, NC*H*, 1H), 7.75 (d, $^3J_{HH}$ = 2.4 Hz, NC*H*, 1H), 7.61 (dd, $^3J_{HH}$ = 7.3 Hz, $^4J_{HH}$ = 1.9 Hz, H^D, 1H), 7.40 (d, $^3J_{HH}$ = 8.0 Hz, H^A, 1H), 7.34 (td, $^3J_{HH}$ = 7.7 Hz, $^4J_{HH}$ = 1.9 Hz, H^B, 1H), 7.28 (t, $^3J_{HH}$ = 7.2 Hz, H^C, 1H), 6.60 (t, $^3J_{HH}$ = 2.5 Hz, CC*H*, 1H), 6.40 (t, $^3J_{HH}$ = 2.5 Hz, CC*H*, 1H), 4.28 (s br, B*H*, 2H), 2.84 (d, $^3J_{HH}$ = 5.5 Hz, NCH_3, 3H).*

^{11}B NMR (160 MHz, $CDCl_3$, 300 K) δ –3.0 (s br, *B*H, 2B).

^{11}B {^{1}H} NMR (160 MHz, $CDCl_3$, 300 K) δ –3.0 (s, *B*H, 2B).

^{13}C {^{1}H} NMR** (126 MHz, $CDCl_3$, 300 K) δ 143.1 (s, C^F, 1C), 140.6 (s, N*C*H, 1C), 139.4 (s, N*C*H, 1C), 138.7 (s, N*C*H, 1C), 137.7 (s, N*C*H, 1C), 137.0 (s, C^D, 1C), 130.3 (s, C^B, 1C), 129.0 (s, C^C, 1C), 125.4 (s, C^A, 1C), 119.9 (q, $^{1}J_{CF}$ = 321 Hz, CF_3, 2C), 108.3 (s, C*C*H, 1C), 107.92 (s, C*C*H, 1C), 44.9 (s, NCH_3, 1C).

^{19}F NMR (471 MHz, $CDCl_3$, 300K) δ –78.6 (s, CF_3, 6F).

*Coupling between the CH_3 (δ 2.84) and N*H* (δ 8.05) was observed by ^{1}H NMR experiment and confirmed by a COSY NMR experiment.

**C^E was not observed by ^{13}C NMR due to broadening due to the quadrupolar B.

Mass Spectrum: HRMS (EI+) m/z calculated for $C_{13}H_{15}B_2N_5^+$: 263.15081; Found: 263.15122.

4.1.3 NMR spectra of the [2]NTf$_2$

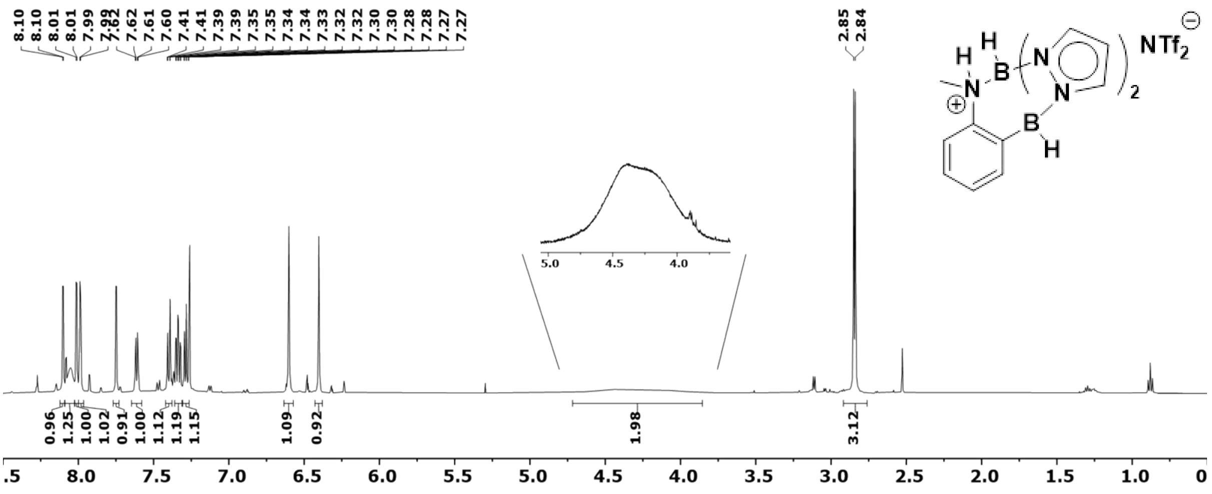

Figure S26: ^{1}H NMR spectrum (500 MHz, $CDCl_3$, 300 K) of the **[2]NTf$_2$**.

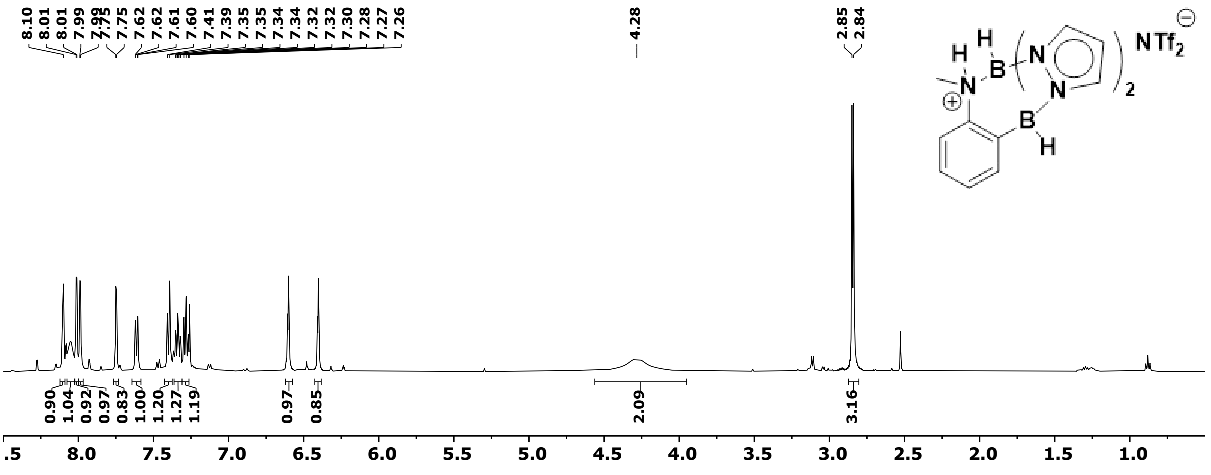

Figure S27: ^{1}H {^{11}B} NMR spectrum (500 MHz, $CDCl_3$, 300 K) of the **[2]NTf$_2$**.

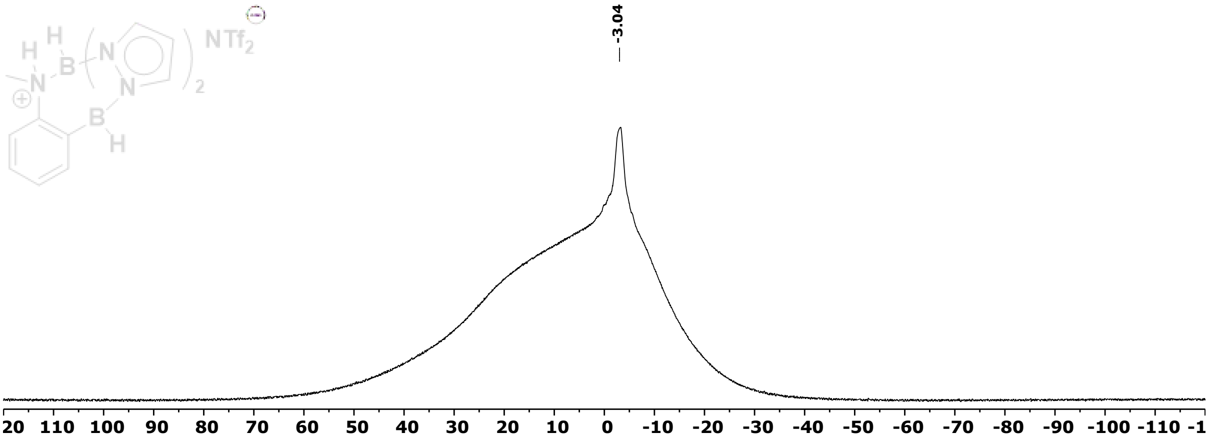

Figure S28: ^{11}B NMR spectrum (160 MHz, $CDCl_3$, 300 K) of the **[2]NTf$_2$**.

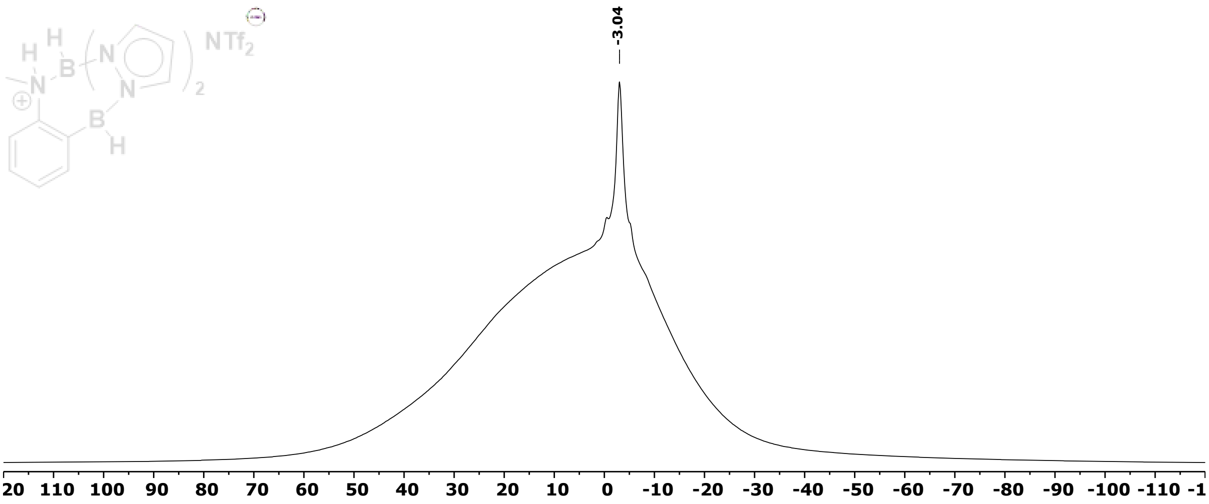

Figure S29: ^{11}B {^{1}H} NMR spectrum (160 MHz, $CDCl_3$, 300 K) of the **[2]NTf$_2$**.

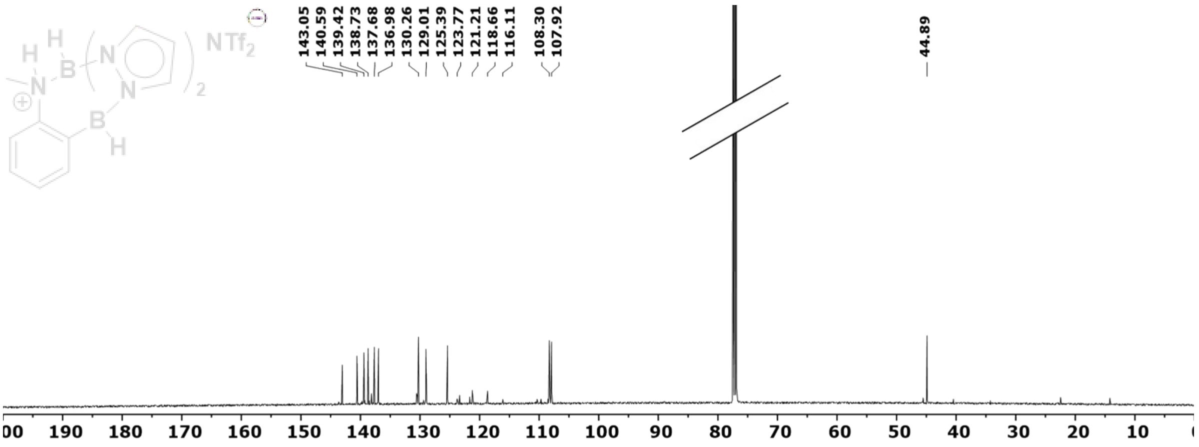

Figure S30: ^{13}C {^{1}H} NMR spectrum (126 MHz, $CDCl_3$, 300 K) of the **[2]NTf$_2$**.

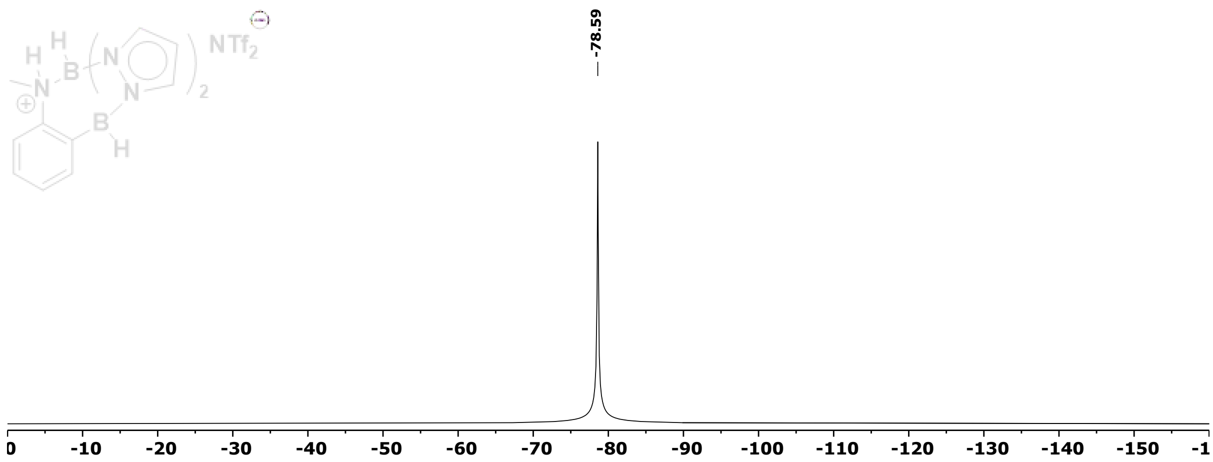

Figure S31: ^{19}F NMR spectrum (471 MHz, $CDCl_3$, 300 K) of the **[2]NTf₂**.

4.1.4 In situ generation of [2]NTf$_2$

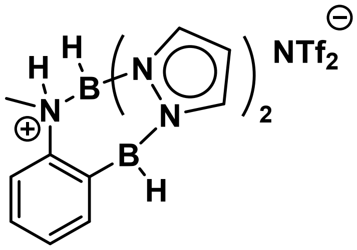

In a J. Young's NMR tube di-NTf$_2$-pyrazabole **1** (72 mg, 0.10 mmol, 1.00 equiv.) was dissolved in $CDCl_3$ (1.5 mL). Et_3N (11 µL, 0.10 mmol, 1.00 equiv.) and N-methylaniline (14 µL, 0.10 mmol, 1.00 equiv.) were successively added. The reaction was heated at 80 °C for 16 hours.

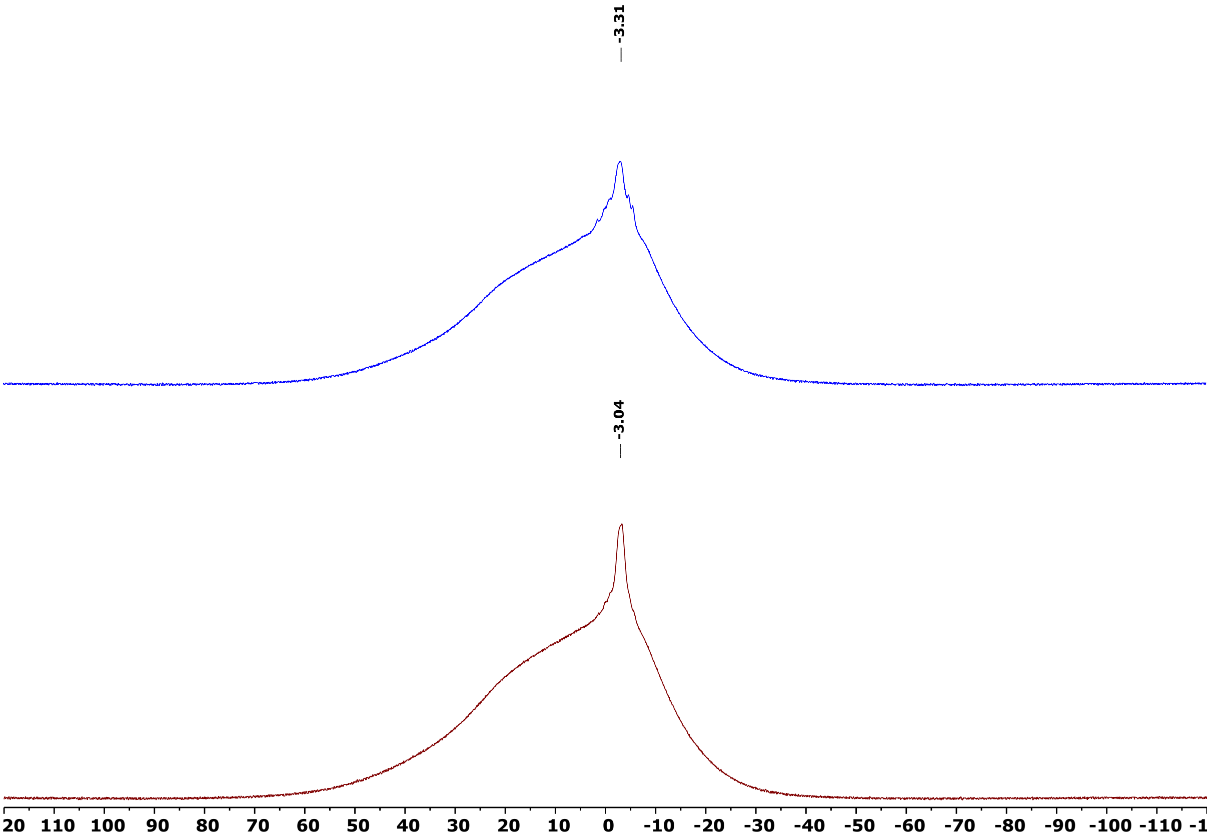

Figure S32: ^{11}B NMR spectrum (160 MHz, $CDCl_3$, 300 K) of the BDB reaction of N-methylaniline using **1** and Et_3N in $CDCl_3$ after heating at 80 °C for 16 hours (top blue). ^{11}B NMR spectrum (160 MHz, $CDCl_3$, 300 K) of **[2]NTf$_2$** generated from **1** and **5** (bottom red).

The main boron signals at δ_{11B} −3.3 (s br) is found almost identical to the one from **[2]NTf$_2$** (δ_{11B} −3.0) generated from **1** and **5** (See SI, Part 4.1.2).

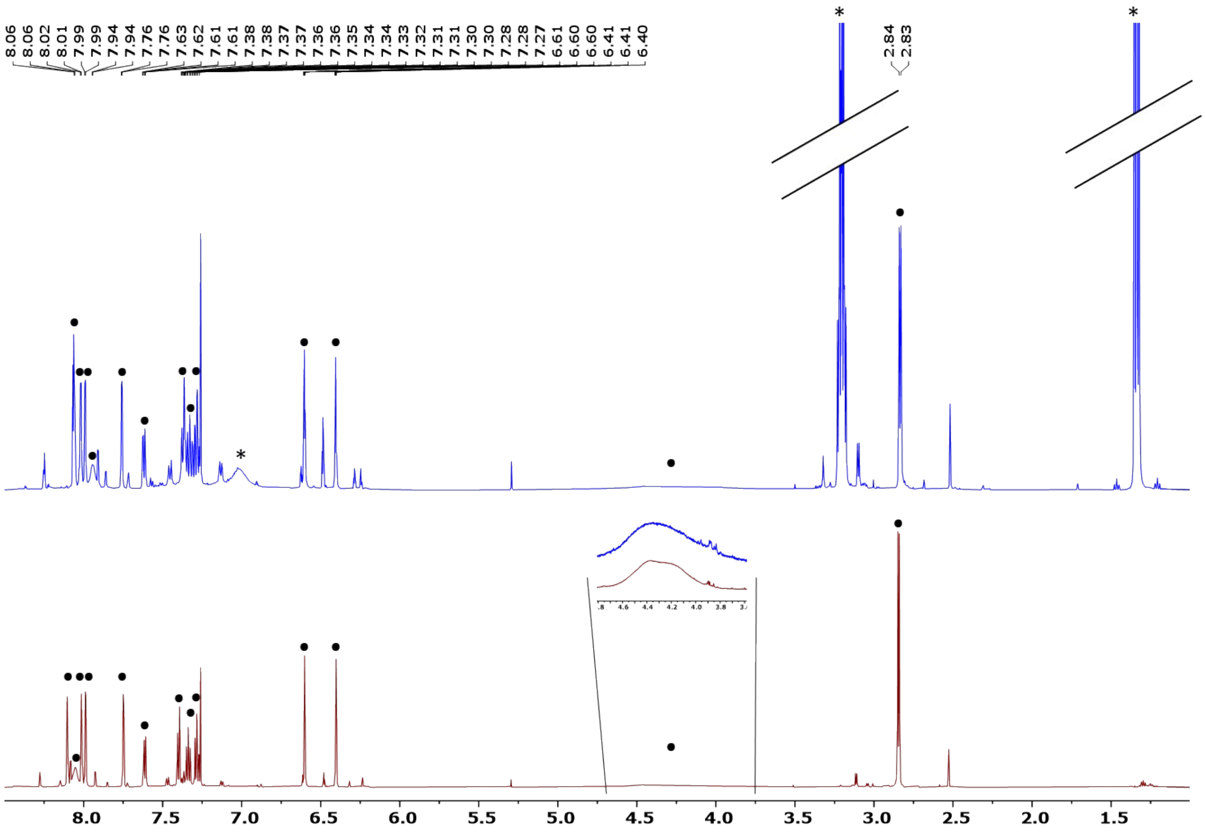

Figure S33: ^{1}H NMR spectrum (500 MHz, $CDCl_3$, 300 K) of the BDB reaction of N-methylaniline using **1** and Et_3N in $CDCl_3$ after heating at 80 °C for 16 hours (top blue). ^{1}H NMR spectrum (500 MHz, $CDCl_3$, 300 K) of **[2]NTf$_2$** generated from **1** and **5** (bottom red).

^{1}H NMR confirmed the nature of the intermediate borylated product generated *in situ*, showing identical signals to the characterised **[2]NTf$_2$** generated from the reaction between **1** and **5** (labelled • on Figure S33). Only the N*H* proton was found slightly shifted between the two experiments (Δδ ≈ 0.1 ppm), presumably due to different H-bonding in the solutions due to by-products present in the *in situ* generation experiment. In the case of the *in situ* generation of **[2]NTf$_2$**, the ^{1}H NMR spectrum also showed signals from protonated base Et_3HNTf_2 (labelled * on Figure S33) and remaining traces of unreacted starting material.

4.2 **BDB with diiodo-pyrazabole 7**

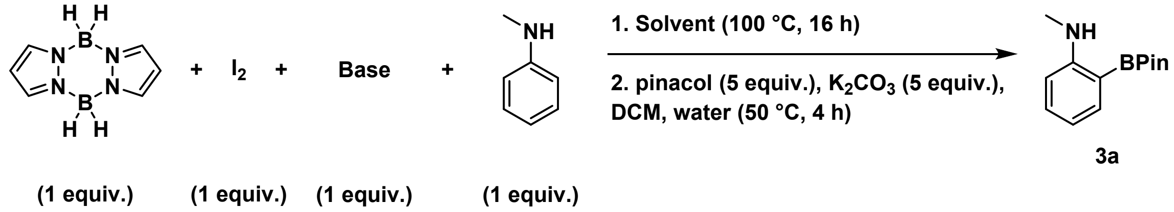

4.2.1 General procedure 1

Borylation:

Pyrazabole (47 mg, 0.30 mmol, 1.00 equiv.) was dissolved in solvent (1.5-2 mL) in a J. Young's ampoule. Iodine (76 mg, 0.30 mmol, 1.00 equiv.) was added at room temperature and the solution was stirred for 2 hours. Base (0.30 mmol, 1.00 equiv.) and N-methylaniline (33 µL, 0.30 mmol, 1.00 equiv.) were successively added to the solution. The reaction mixture was heated at 100 °C for 16 hours.

Pinacol protection:

The solution was cooled down to room temperature. Pinacol (177 mg, 1.50 mmol, 5.00 equiv.), K_2CO_3 (207 mg, 1.50 mmol, 5.00 equiv.), DCM (2.8 mL) and water (2.8 mL) were sequentially added. The resulting biphasic mixture was heated at 50 °C for 4 hours while stirring vigorously. The organic phase was extracted with pentane (4 x 10 mL). The combined organic phases were washed with water (4 x 10 mL), dried over $MgSO_4$, and filtered. Volatiles were removed *in vacuo*.

4.2.2 Results

Screening experiments were carried out using the diiodo-pyrazabole **7** system for *ortho*-borylation of N-methylaniline. These experiments have been done using **General procedure 1** (See SI, Part 4.2.1). Variations from **General procedure 1** and outcomes of each experiment are listed in the table below.

Entry	Solvent	Base	Other variation(s)	Yield	Comments
**1**	**PhCl**	**Et_3N**	**-**	**47%**	**Pure product isolated**
**2**	**PhCl**	**Et_3N**	**Isolated diiodo-pyrazabole (1 equiv.) instead of *in situ* activation with Iodine**	**51%**	**Pure product isolated**
3	PhCl	Et_3N	Borylation: 100 °C, 24 h<br>Pinacol protection: 50 °C, 16 h	51% (30%)	Isolated yield with 95% purity (100% purity, after celite filtration)
4	PhCl	Et_3N	Borylation: 100 °C, 72 h	50%	Pure product isolated
5	PhCl	Et_3N	Pyrazabole (1.2 equiv.) Iodine (1.2 equiv.)<br>Borylation: 100 °C, 24 h<br>Pinacol protection: 50 °C, 16 h	49%	Pure product isolated
6	PhCl	Et_3N	Pyrazabole (2 equiv.) Iodine (2 equiv.)	48%	Pure product isolated (< 5% N-methylaniline by NMR)
7	PhCl	Et_3N	Et_3N (2 equiv.)	< 30%	Contaminated with side products
8	PhCl	Et_3N	Et_3N (3 equiv.)	< 30%	Contaminated with side products
9	PhCl	Et_3N	N-methylaniline (2 equiv.)	< 10%	Trace of product observed
10	PhCl	Et_3N	N-methylaniline added 25 min before Et_3N addition	41%	Isolated yield with 95% purity
11	PhF_2	Et_3N	-	54%	Pure product isolated
12	Toluene	Et_3N	-	< 10%	Trace of product observed
13	PhCl	2,6-lutidine	-	< 30%	Contaminated with side products
14	PhCl	DBU	-	< 10%	Trace of product observed
15	PhCl	DABCO	-	< 1%	No borylation observed
16	PhCl	DIPEA	-	< 30%	Contaminated with side products
17	PhCl	2,6-di-*tert*-butyl-4-methylpyridine	-	< 30%	Contaminated with side products
18	PhCl	None	N-methylaniline (2 equiv.)	< 10%	Trace of product observed

Table S2: List of the conditions and results of the screening reactions from the optimisation attempts of BDB with diiodo-pyrazabole **7**.

4.3 BDB with dibromo-pyrazabole

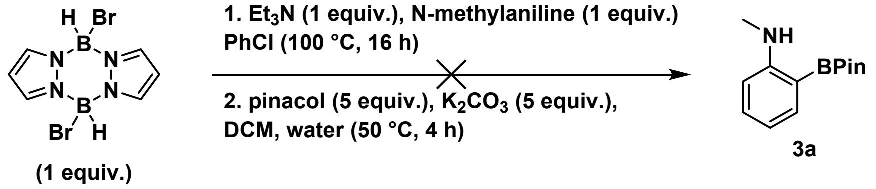

Dibromo-pyrazabole (95 mg, 0.30 mmol, 1.00 equiv.) was dissolved in PhCl (2 mL) at room temperature in a J. Young's ampoule. Et_3N (42 µL, 0.30 mmol, 1.00 equiv.) and N-methylaniline (33 µL, 0.30 mmol, 1.00 equiv.) were successively added to the solution. The reaction mixture was heated at 100 °C for 16 hours. The solution was cooled down to room temperature. Pinacol (177 mg, 1.50 mmol, 5.00 equiv.), K_2CO_3 (207 mg, 1.50 mmol, 5.00 equiv.), DCM (2.8 mL) and water (2.8 mL) were sequentially added. The resulting biphasic mixture was heated at 50 °C for 4 hours. The product was extracted with pentane (4 x 10 mL). The combined organic phases were washed with water (4 x 10 mL), dried over $MgSO_4$, and filtered. Volatiles were removed *in vacuo*. NMR analysis only revealed starting material and the di-N-methylaniline-pyrazabole **5**, without any borylated product **3a**.

5 Mechanistic studies

5.1 Characterisation of compound 4

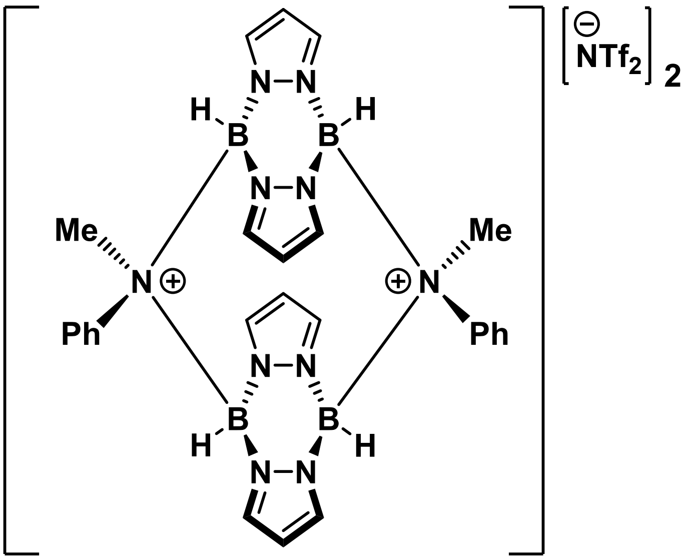

Di-NTf$_2$-pyrazabole **1** (20 mg, 0.03 mmol, 1.00 equiv.) and di-N-methylaniline-pyrazabole **5** (10 mg, 0.03 mmol, 1.00 equiv.) were dissolved in $CDCl_3$ and left at room temperature for 1 hour, in a J. Young's NMR tube. Almost quantitative conversion to a new product was observed, which was assigned to the dimer **4**.

^{1}H NMR (500 MHz, $CDCl_3$, 300 K) δ 8.28 (d, $^{3}J_{HH}$ = 2.4 Hz, NC*H*, 4H), 7.93 (d, $^{3}J_{HH}$ = 2.5 Hz, NC*H*, 4H), 7.49-7.43 (m, ^{ortho}C*H*, 4H), 7.39-7.32 (m, ^{meta}C*H*, 4H), 7.28-7.23 (m, ^{para}C*H*, 2H), 6.61 (t, $^{3}J_{HH}$ = 2.4 Hz, CC*H*, 2H), 6.23 (t, $^{3}J_{HH}$ = 2.4 Hz, CC*H*, 2H), 5.26-3.95 (m br, B*H*, 4H), 2.52 (s, CH_3, 6H).

^{1}H {^{11}B} NMR (500 MHz, $CDCl_3$, 300 K) δ 8.28 (d, $^{3}J_{HH}$ = 3.2 Hz, NC*H*, 4H), 7.93 (d, $^{3}J_{HH}$ = 3.1 Hz, NC*H*, 4H), 7.53-7.42 (m, ^{ortho}C*H*, 4H), 7.41-7.30 (m, ^{meta}C*H*, 4H), 7.29-7.20 (m, ^{para}C*H*, 2H), 6.61 (t, $^{3}J_{HH}$ = 2.7 Hz, CC*H*, 2H), 6.23 (t, $^{3}J_{HH}$ = 2.8 Hz, CC*H*, 2H), 4.65 (s br, B*H*, 4H), 2.52 (s, CH_3, 6H).

^{11}B NMR (160 MHz, $CDCl_3$, 300 K) δ –0.4 (d, $^{1}J_{BH}$ = 150 Hz, *B*H).

^{11}B {^{1}H} NMR (160 MHz, $CDCl_3$, 300 K) δ –0.4 (s, *B*H).

^{13}C {^{1}H} NMR (126 MHz, $CDCl_3$, 300 K) δ 143.7 (s, ^{ipso}C, 2C), 138.1 (s, N*C*H, 4C), 137.0 (s, N*C*H, 4C), 130.6 (s, ^{meta}*C*H, 4C), 128.9 (s, ^{para}*C*H, 2C), 123.4 (s, ^{ortho}*C*H, 4C), 120.06 (q, $^{1}J_{CF}$ = 322 Hz, CF_3, 4C), 110.4 (s, C*C*H, 2C), 109.7 (s, C*C*H, 2C), 45.6 (s, NCH_3, 2C).

^{19}F NMR (471 MHz, $CDCl_3$, 300K) δ –78.7 (s, CF_3, 12F).

Mass Spectrum: Compound **4** was unstable to mass spectrometry analysis, only the monomeric cation was observed.

5.2 **NMR spectra of compound 4**

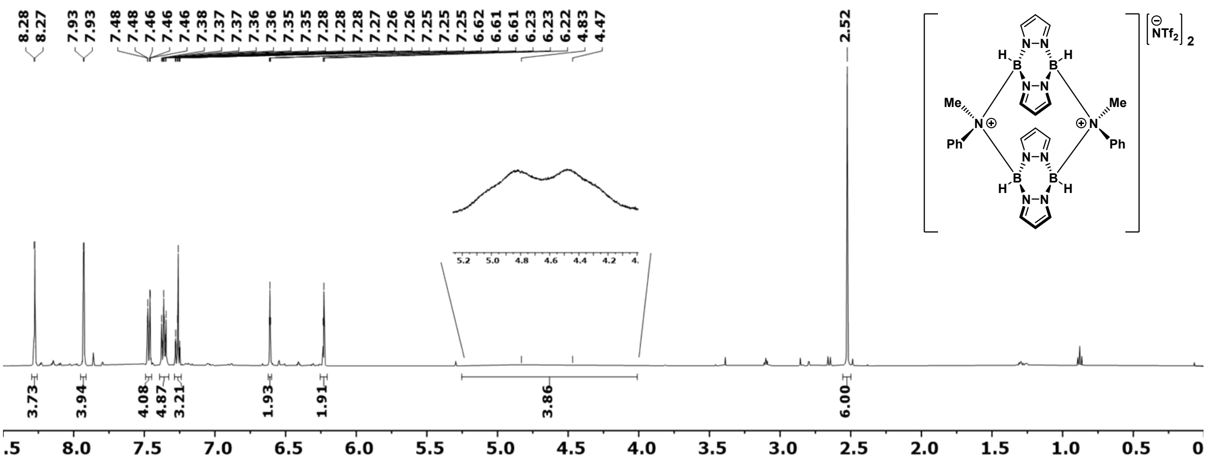

Figure S34: ^{1}H NMR spectrum (500 MHz, $CDCl_3$, 300 K) of **4**.

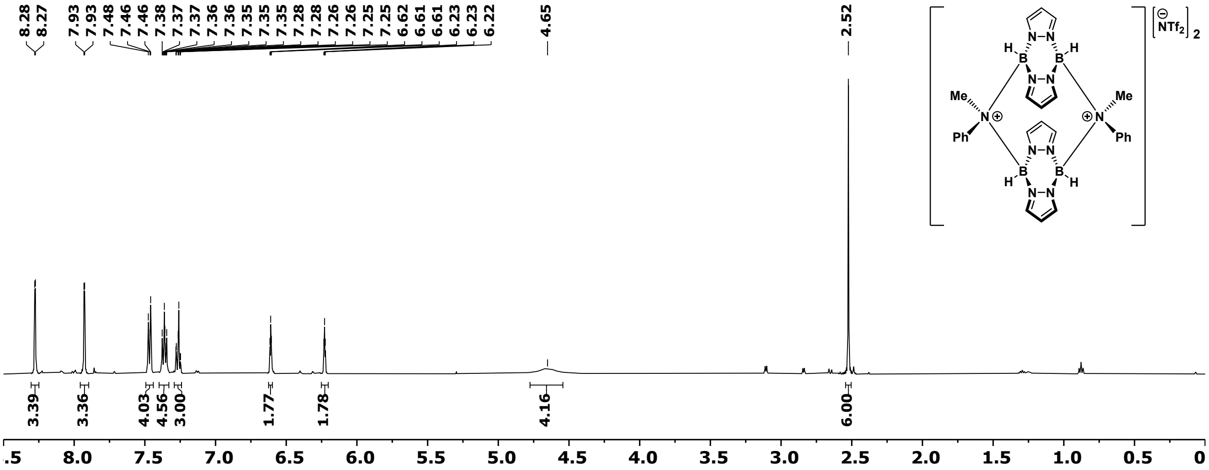

Figure S35: ^{1}H {^{11}B} NMR spectrum (500 MHz, $CDCl_3$, 300 K) of **4**.

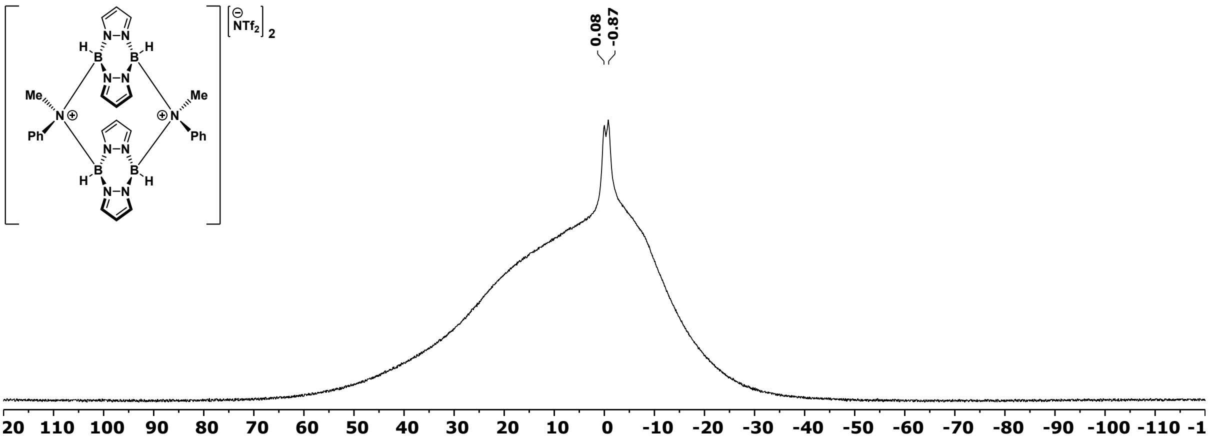

Figure S36: ^{11}B NMR spectrum (160 MHz, $CDCl_3$, 300 K) of **4**.

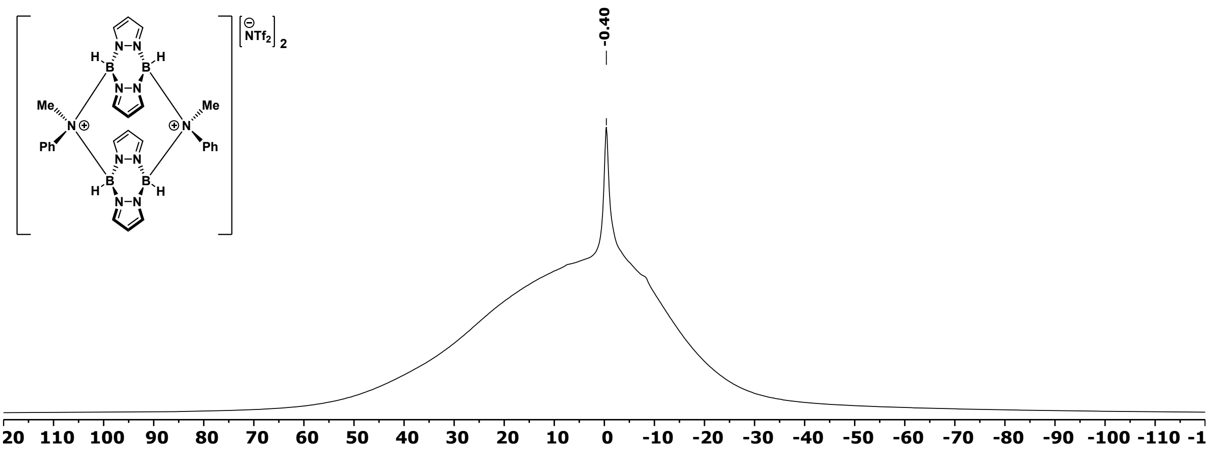

Figure S37: ^{11}B {^{1}H} NMR spectrum (160 MHz, $CDCl_3$, 300 K) of **4**.

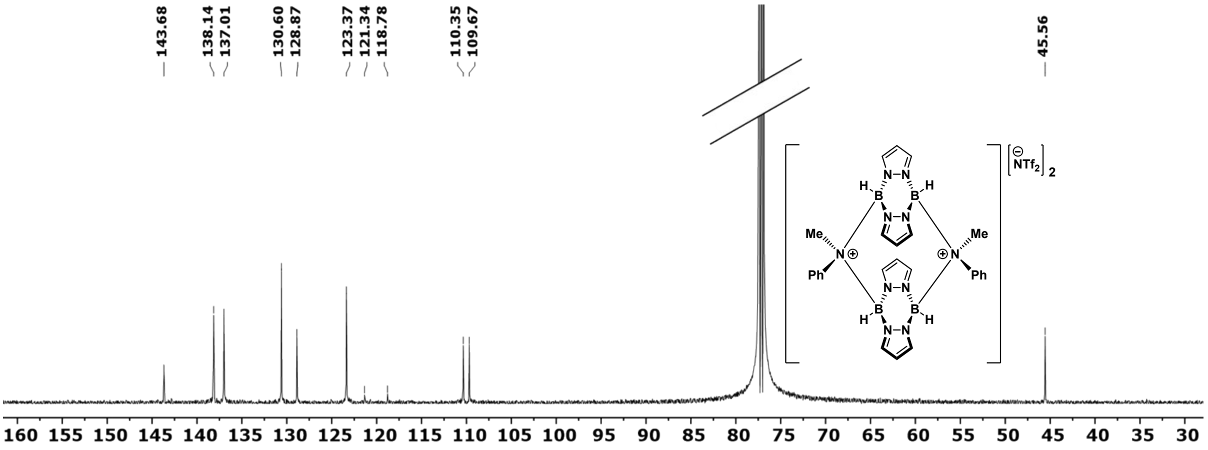

Figure S38: ^{13}C {^{1}H} NMR spectrum (126 MHz, $CDCl_3$, 300 K) of **4**.

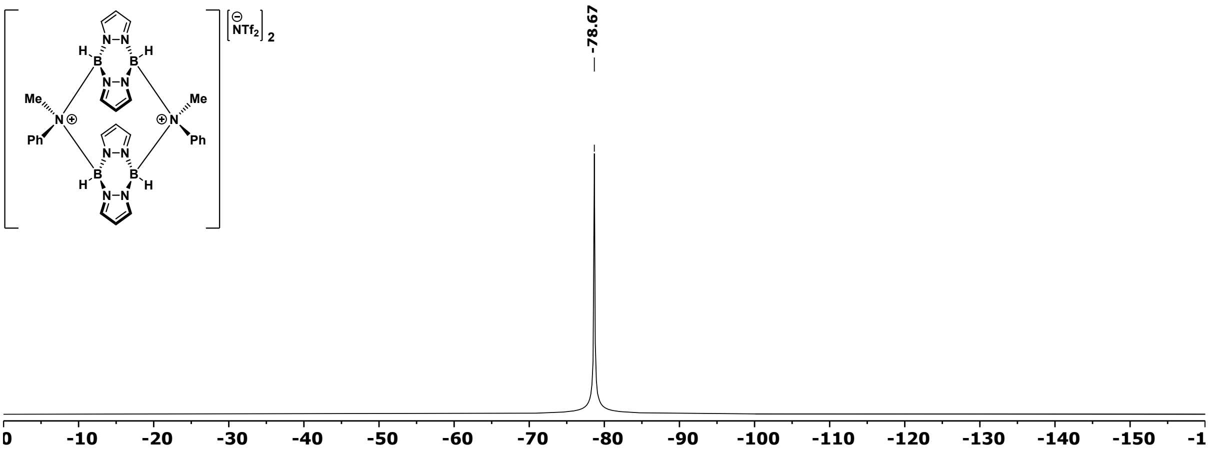

Figure S39: ^{19}F NMR spectrum (471 MHz, $CDCl_3$, 300K) of **4**.

5.3 **DOSY experiments**

The normalization of the diffusion coefficients was carried out following the method published by Stalke *et al.*[S10]

Adamantane was used as internal standard.

5.3.1 DOSY of di-N-methylaniline-pyrazabole 5

Di-N-methyl-aniline-pyrazabole **5** was considered to diffuse like dissipated spheres and ellipsoids (DSE).

A solution of $CDCl_3$ (0.4 mL) with di-N-methylaniline-pyrazabole **5** (6 mg, 40 mM) and adamantane (2 mg, 40 mM) was prepared in a J. Young's NMR tube. The chosen concentrations were within the range of the valid concentrations (< 120 mM).[S10] The normalization of the diffusion coefficient was calculated using the following equation.

$$\log D_{substrate,norm} = \log D_{ref,fix} - log\ D_{ref} + log\ D_{substrate}$$

The value *log* $D_{\mathrm{ref,fix}}$ = –8.8155 was found in the literature from Stalke *et al.*[S11]

The values of *log* D_{ref} = –8.192 and *log* $D_{\mathrm{substrate}}$ = –9.0707 were measured experimentally by DOSY NMR experiment.

$$\log D_{substrate,norm} = -8.8155 + 8.8192 - 9.0707$$

$$\log D_{substrate,norm} = -9.0670$$

$$D_{substrate,norm} = 8.5707 \times 10^{-10}$$

The molecular weight was calculated using the power law developed by Williard *et al.*[S12]

$$D = K \times MW^{\alpha}$$

As considered diffusing like DSE, the value of *log K* = –7.59 and α = –0.572 were found from the literature.[S11]

$$D_{substrate,norm} = 8.5707 \times 10^{-10} = 2.5704 \times 10^{-8} \times MW^{-0.572}$$

$$MW = 381.99$$

The experimental molecular weight determination of the di-N-methylaniline-pyrazabole **5**, *MW* = 381.99 $g.mol^{-1}$ was found within the range of the theoretical value (370.07 $g.mol^{-1}$) with 3% error.

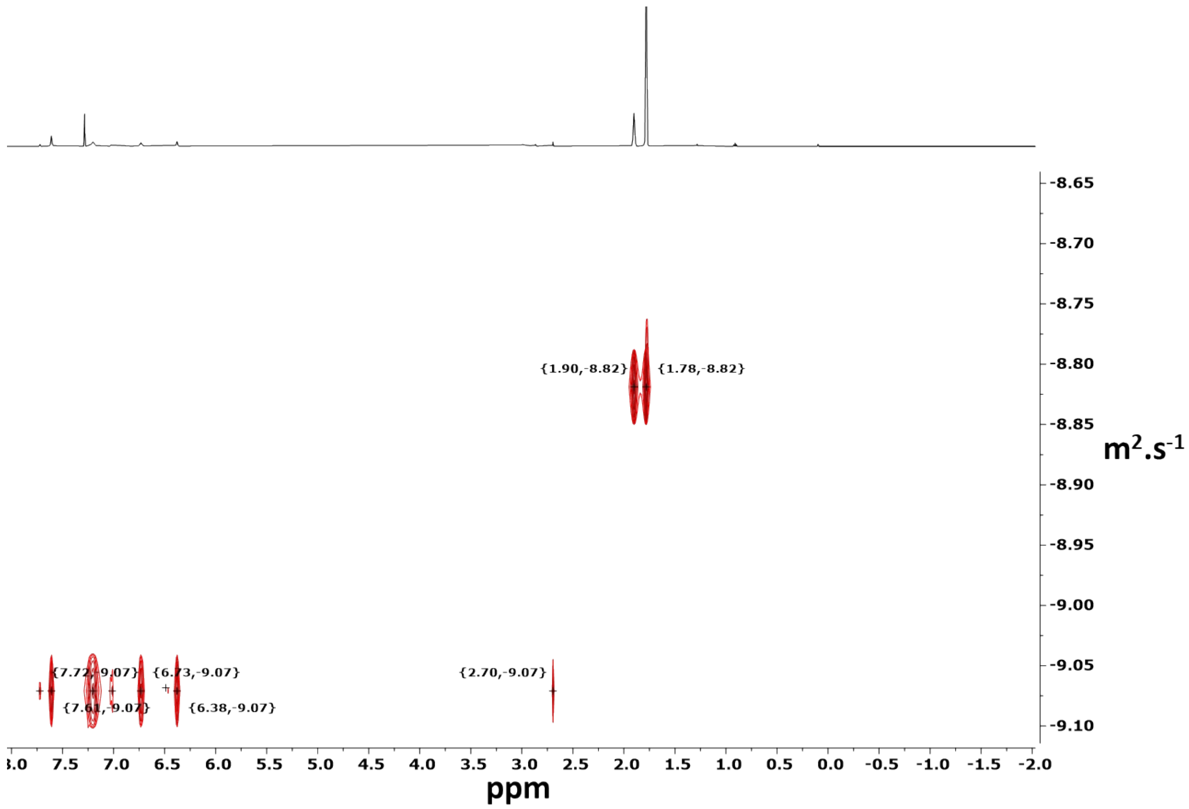

Figure S40: DOSY spectrum of the di-N-methylaniline-pyrazabole **5** with internal standard adamantane.

5.3.2 DOSY of 4

Compound **4** was considered to diffuse like dissipated spheres and ellipsoids (DSE).

A solution of **4** (10 mM) in $CDCl_3$ was prepared by adding di-NTf_2-pyrazabole **1** (20 mg, 0.03 mmol, 1.00 equiv.) and di-N-methylaniline-pyrazabole **5** (10 mg, 0.03 mmol, 1.00 equiv.) in $CDCl_3$ (1.5 mL) in a J. Young's NMR tube. After leaving the solution overnight, the internal standard, adamantane (4 mg, 20 mM) was added. The chosen concentrations were within the range of the valid concentrations (< 120 mM).[S10] The normalization of the diffusion coefficient was calculated using the following equation.

$$\log D_{substrate,norm} = \log D_{ref,fix} - log\ D_{ref} + log\ D_{substrate}$$

The value $log\ D_{\mathrm{ref,fix}}$ = –8.8155 was found in the literature from Stalke *et al.*[S11]

The values of $log\ D_{\mathrm{ref}}$ = –8.8189 and $log\ D_{\mathrm{substrate}}$ = –9.2282 were measured experimentally by DOSY NMR experiment.

$$\log D_{substrate,norm} = -8.8155 + 8.8189 - 9.2282$$

$$\log D_{substrate,norm} = -9.2248$$

$$D_{substrate,norm} = 5.9593 \times 10^{-10}$$

The molecular weight was calculated using the power law developed by Williard *et al.*[S12]

$$D = K \times MW^{\alpha}$$

As considered diffusing like DSE, the value of $log\ K$ = –7.59 and α = –0.572 were found from the literature.[S11]

$$D_{substrate,norm} = 5.9593 \times 10^{-10} = 2.5704 \times 10^{-8} \times MW^{-0.572}$$

$$MW = 723.46$$

Theoretical masses for various possible species present:

$MW_{[\text{dicationic fragment of } \mathbf{4}]2+}$ = 527.85 g.mol^{-1}

$MW_{[\text{dicationic fragment of } \mathbf{4}]2+[\text{NTf2}]-}$ = 807.99 g.mol^{-1}

$MW_{\mathbf{4}}$ = 1088.12 g.mol^{-1}

$MW_{[\text{cationic monomer fragment of } \mathbf{4}]+[\text{NTf2}]-}$ = 544.06 g.mol^{-1}

The experimental molecular weight determination of **4**, MW = 723.46 g.mol^{-1} was found different from the expected possible values. However it was found within the range of

heavier and likely dimeric structure. The difference in mass found can be explained by the ionic nature of **4**, which is in an equilibrium between the dication and one anion diffusing together and the dication and anions diffusing separately.

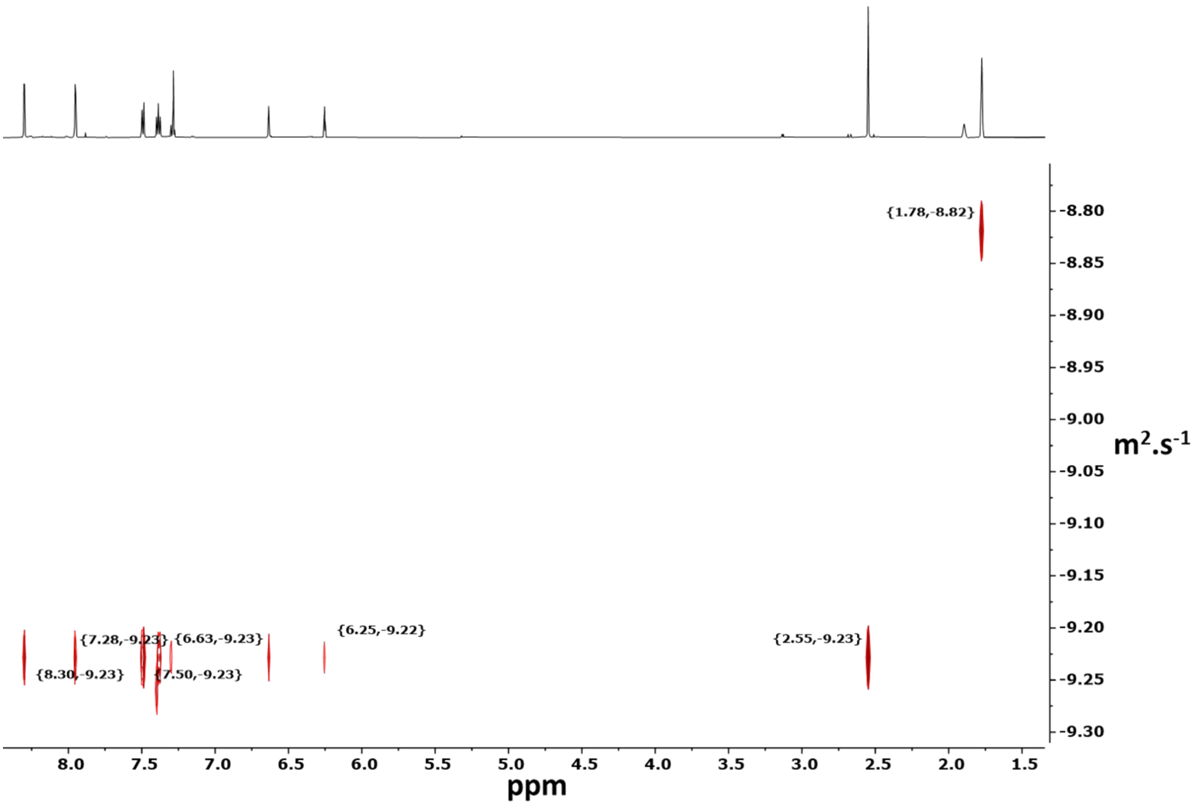

Figure S41: DOSY spectrum of **4** with its internal standard adamantane.

5.4 In situ generation of compound 4

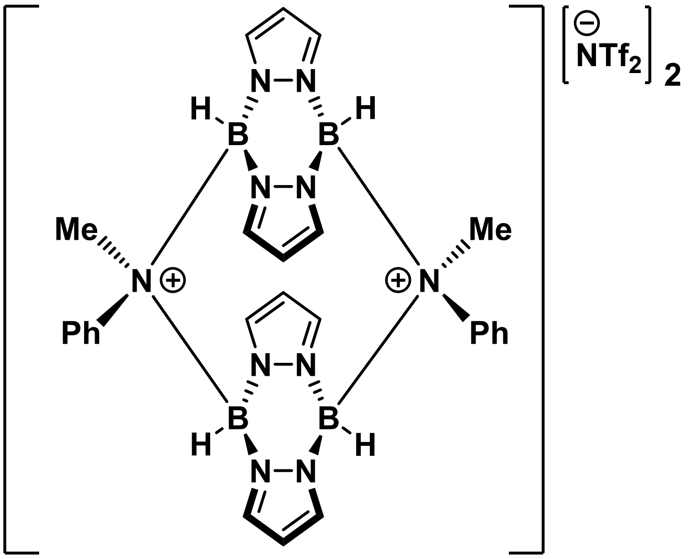

In a J. Young's NMR tube di-NTf_2-pyrazabole **1** (72 mg, 0.10 mmol, 1.00 equiv.) was dissolved in $CDCl_3$ (1.5 mL). Et_3N (11 µL, 0.10 mmol, 1.00 equiv.) and N-methylaniline (14 µL, 0.10 mmol, 1.00 equiv.) were successively added. The reaction mixture was left for 15 min before ^1H and ^{11}B NMR analysis.

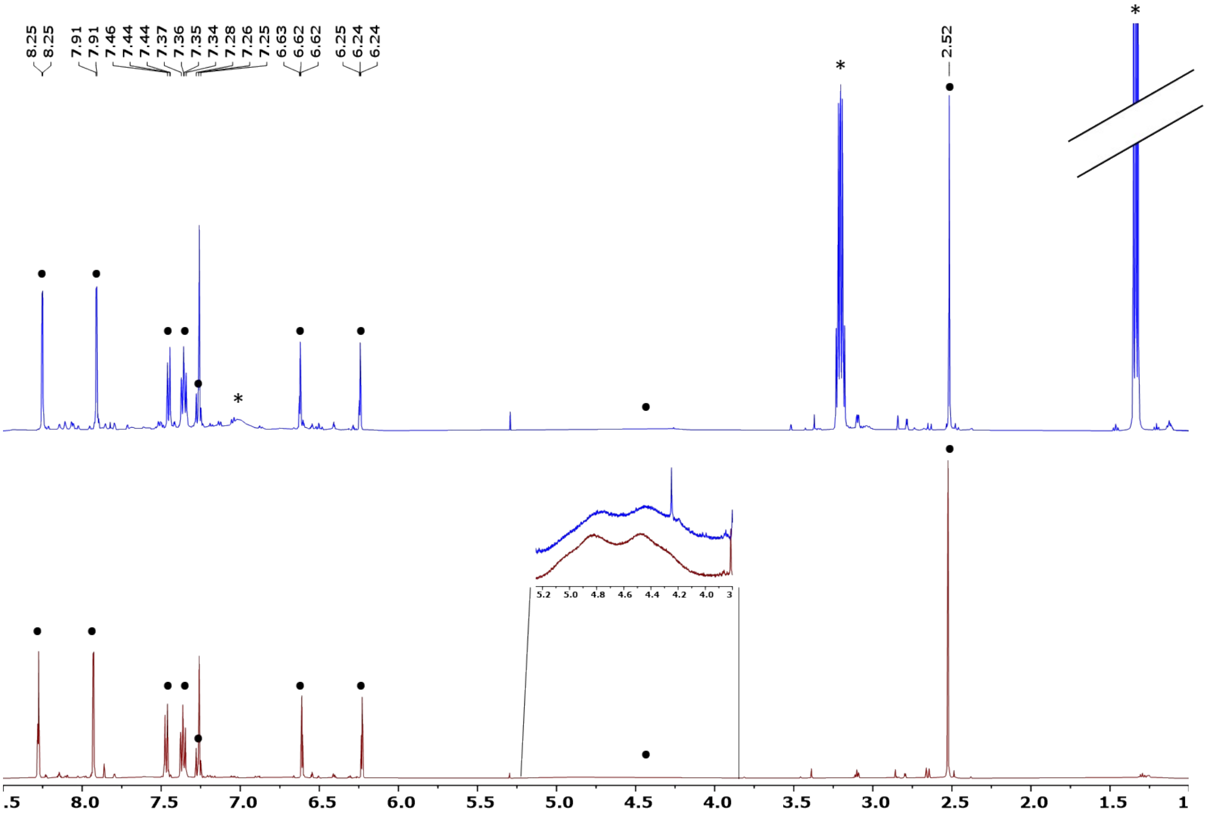

Figure S42: ^1H NMR spectrum (500 MHz, $CDCl_3$, 300 K) of the BDB reaction of N-methylaniline using **1** and Et_3N in $CDCl_3$ after 15 minutes at room temperature (top blue). ^1H NMR spectrum (500 MHz, $CDCl_3$, 300 K) of **4** generated from **1** and **5** (bottom red).

^1H NMR confirmed the nature of the intermediate product generated *in situ*, showing identical signals to the characterised **4** generated from the reaction between **1** and **5** (labelled • on Figure S42). In the case of the *in situ* generation of **4**, the ^1H NMR spectrum also showed signals from protonated base Et_3HNTf_2 (labelled * on Figure S42) and remaining traces of unreacted starting material.

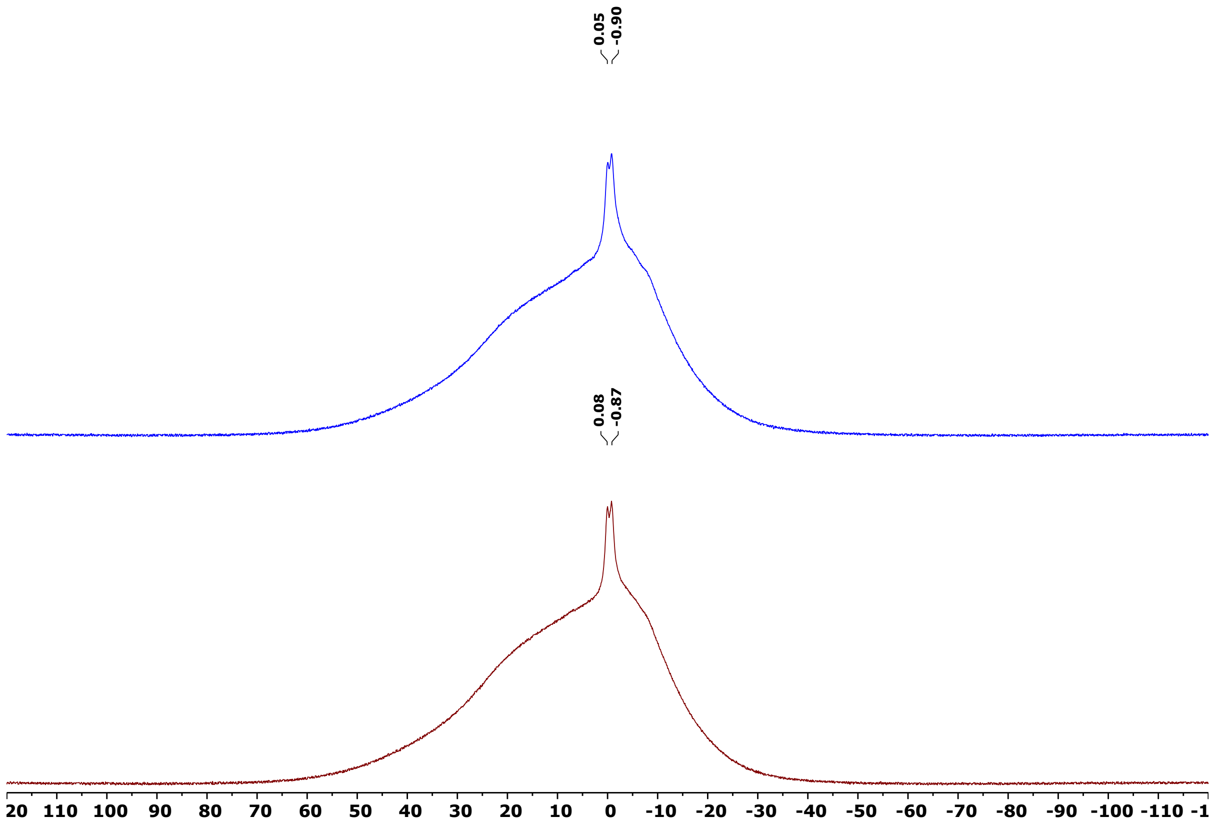

Figure S43: ^{11}B NMR spectrum (160 MHz, $CDCl_3$, 300 K) of the borylation reaction of N-methylaniline using **1** and Et_3N in $CDCl_3$ after 15 minutes at room temperature (top blue). ^{11}B NMR spectrum (160 MHz, $CDCl_3$, 300 K) of **4** generated from **1** and **5** (bottom red).

Comparison of the ^{11}B NMR from the formed *in situ* intermediate and **4** generated from **1** and **5** revealed identical boron chemical shift (δ_{11B} −0.4).

5.5 **Study of the diiodo-pyrazabole 7**

5.5.1 ***Conversion study and in-situ monitoring***

5.5.1.1 Sub-stoichiometric iodine addition (0.5 equiv.)

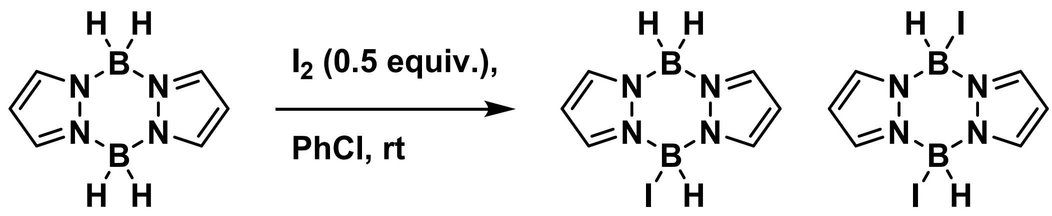

Pyrazabole (10 mg, 0.06 mmol, 1.00 equiv.) was dissolved in PhCl (0.8 mL) in a J. Young's NMR tube. Iodine (8 mg, 0.03 mmol, 0.50 equiv.) was added. The solution was left to react at room temperature and the conversion was monitored by ^{11}B NMR spectroscopy.

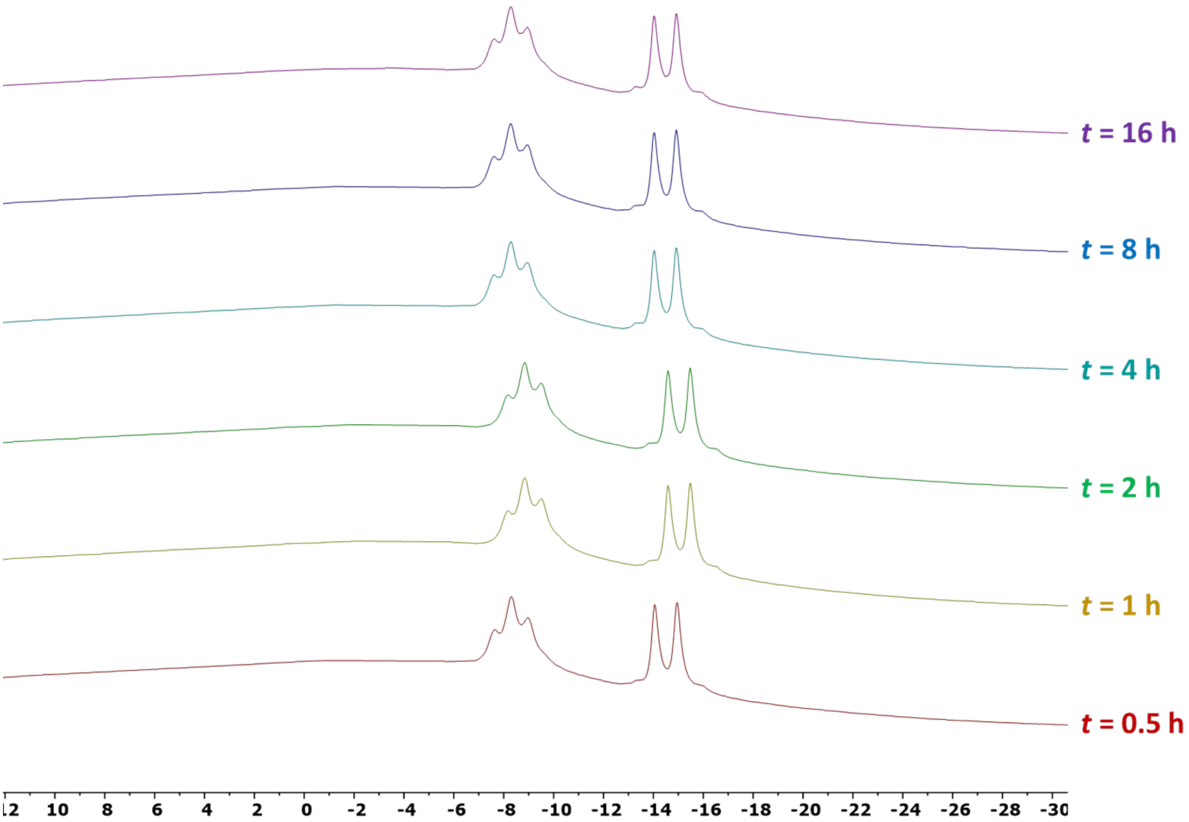

Figure S44: ^{11}B NMR spectra (160 MHz, PhCl, 300 K) of the conversion monitoring from the reaction between pyrazabole (1.00 equiv.) and iodine (0.50 equiv.).

^{11}B NMR monitoring revealed a rapid reaction with the mono-iodo-pyrazabole **6** as the main product (δ –8.3, t, $^{1}J_{BH}$ = 107 Hz; –14.5, d, $^{1}J_{BH}$ = 145 Hz) along with a small amount

of the diiodo-pyrazabole **7** (δ –14.0, d, $^{1}J_{BH}$ = 145 Hz; –15.7, d, $^{1}J_{BH}$ = 151 Hz) and remaining unreacted pyrazabole (δ –8.9, t, $^{1}J_{BH}$ = 101 Hz).

The overreaction (leading to the diiodo-pyrazabole **7**) happens rapidly even under sub-stoichiometric iodine addition. This made the isolation of the mono-iodo-pyrazabole **6** challenging and we instead focused on making and isolating the diiodo-pyrazabole **7** as it was key in this study.

5.5.1.2 Stoichiometric iodine addition (1 equiv.)

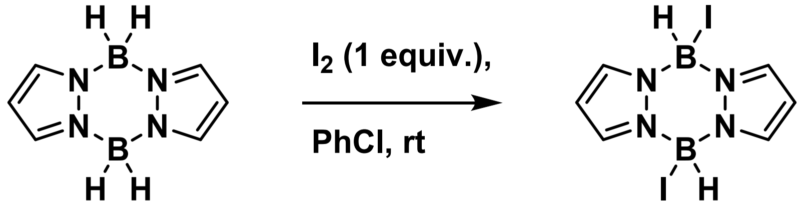

Pyrazabole (10 mg, 0.06 mmol, 1.00 equiv.) was dissolved in PhCl (1.4 mL) in a J. Young's NMR tube. Iodine (16 mg, 0.06 mmol, 1.00 equiv.) was added. The reaction was left to react at room temperature and the conversion was monitored by ^{11}B NMR spectroscopy.

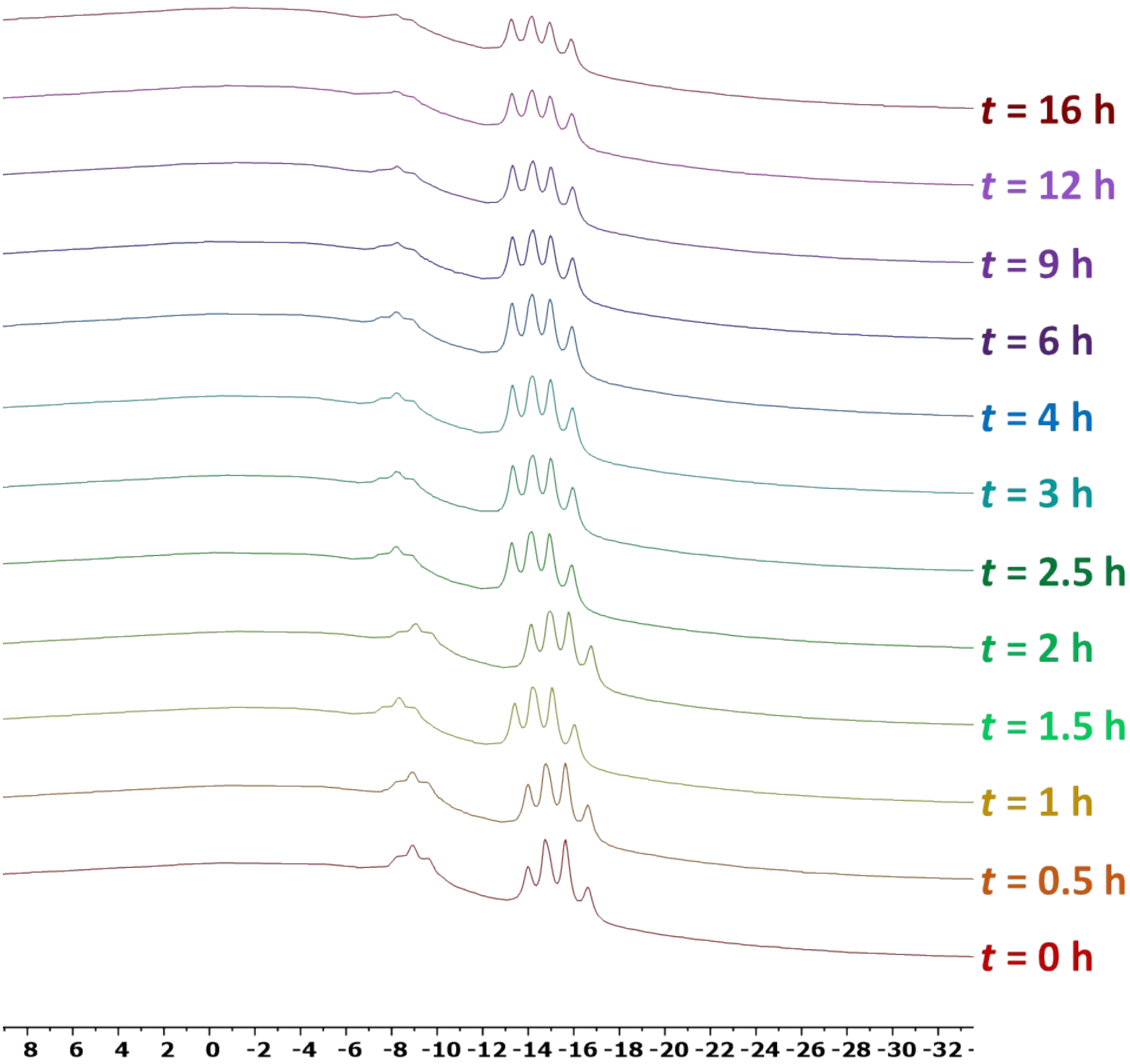

Figure S45: ^{11}B NMR spectra (160 MHz, PhCl, 300 K) of the conversion monitoring from the reaction between pyrazabole (1.00 equiv.) and iodine (1.00 equiv.).

^{11}B NMR monitoring revealed immediate formation of a mixture of products containing the mono-iodo-pyrazabole **6** (δ −8.3, t, $^{1}J_{BH}$ = 107 Hz; −14.5, d, $^{1}J_{BH}$ = 145 Hz) along with the two isomers of the diiodo-pyrazabole **7** (δ −14.0, d, $^{1}J_{BH}$ = 145 Hz; −15.7, d, $^{1}J_{BH}$ = 151 Hz) and remaining unreacted pyrazabole (δ −8.9, t, $^{1}J_{BH}$ = 101 Hz).

Overtime, the mono-iodo-pyrazabole **6** and the pyrazabole are consumed, asserted by the decrease of their respective signals. In parallel, the respective doublets from the *A*- and *B*-***7*** isomers are increasing, showing the overtime conversion to the final product **7**.

The reaction mixture changed rapidly between the time of addition and 2.5 hours. The conversion then slowed down overtime, and had reached a static point after 16 hours.

5.5.2 Stability study

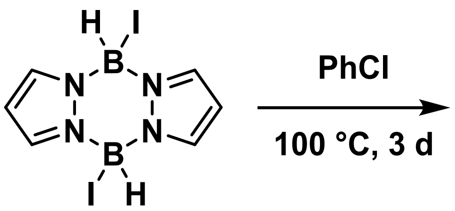

Diiodo-pyrazabole **7** (10 mg, 0.03 mmol, 1.00 equiv.) was dissolved in PhCl (1 mL) in a J. Young's NMR tube and heated at 100 °C for 3 days.

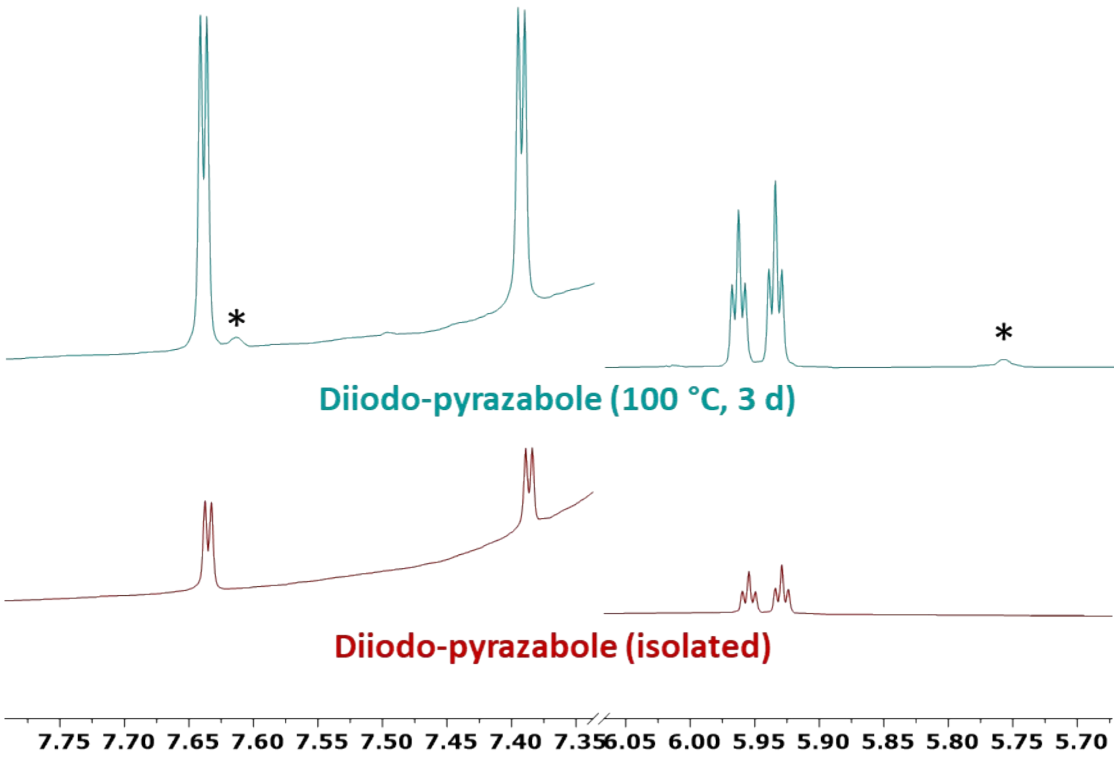

Figure S46: ^{1}H NMR spectra (500 MHz, PhCl, 300 K) of an isolated sample of diiodo-pyrazabole **7** (bottom red) and of the diiodo-pyrazabole **7** after heating at 100 °C over 3 days (top blue). Solvent region peak has been cut for clarity.

After 3 days of heating, only minor signals of decomposition (< 5%) were observed by ^{1}H NMR (labelled * on Figure S46).

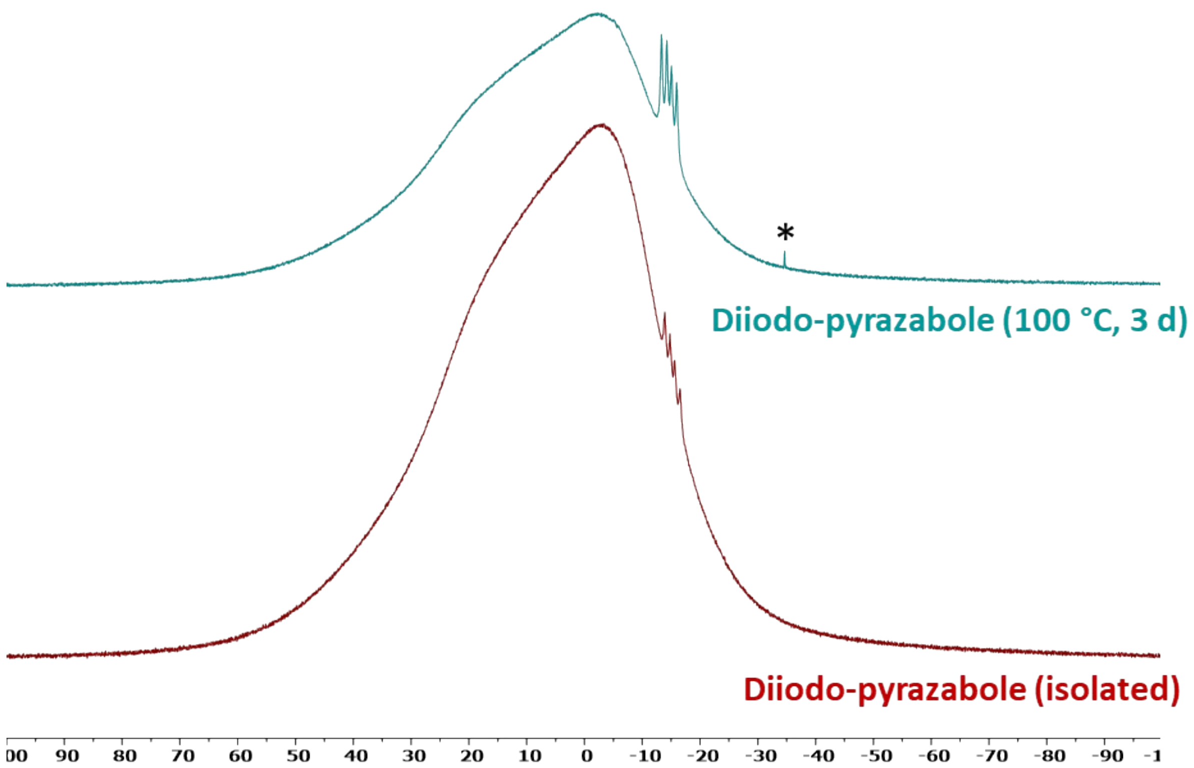

Figure S47: ^{11}B NMR spectra (160 MHz, PhCl, 300 K) of an isolated sample of diiodo-pyrazabole **7** (bottom red) and of the diiodo-pyrazabole **7** after heating at 100 °C over 3 days (top blue).

After 3 days of heating, only minor signals of decomposition (δ –34.6) were observed by ^{11}B NMR (labelled * on Figure S47).

5.5.3 Reaction between Diiodo-pyrazabole 7 and pyrazabole

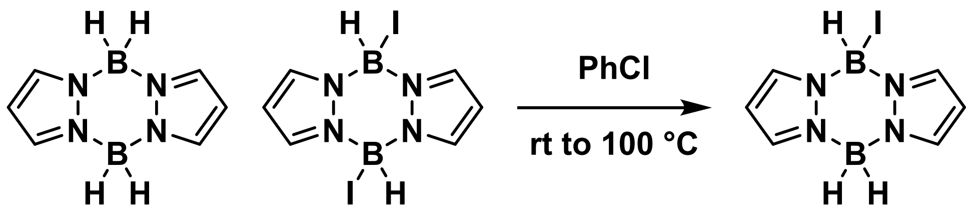

Pyrazabole (16 mg, 0.10 mmol, 1.00 equiv.) and diiodo-pyrazabole **7** (42 mg, 0.10 mmol, 1.00 equiv.) were dissolved in PhCl (0.5 mL) in a J. Young's NMR tube. The reaction was left at room temperature for 0.5 hour before being heated at 100 °C for 1 hour. Conversion was monitored by ^{11}B NMR.

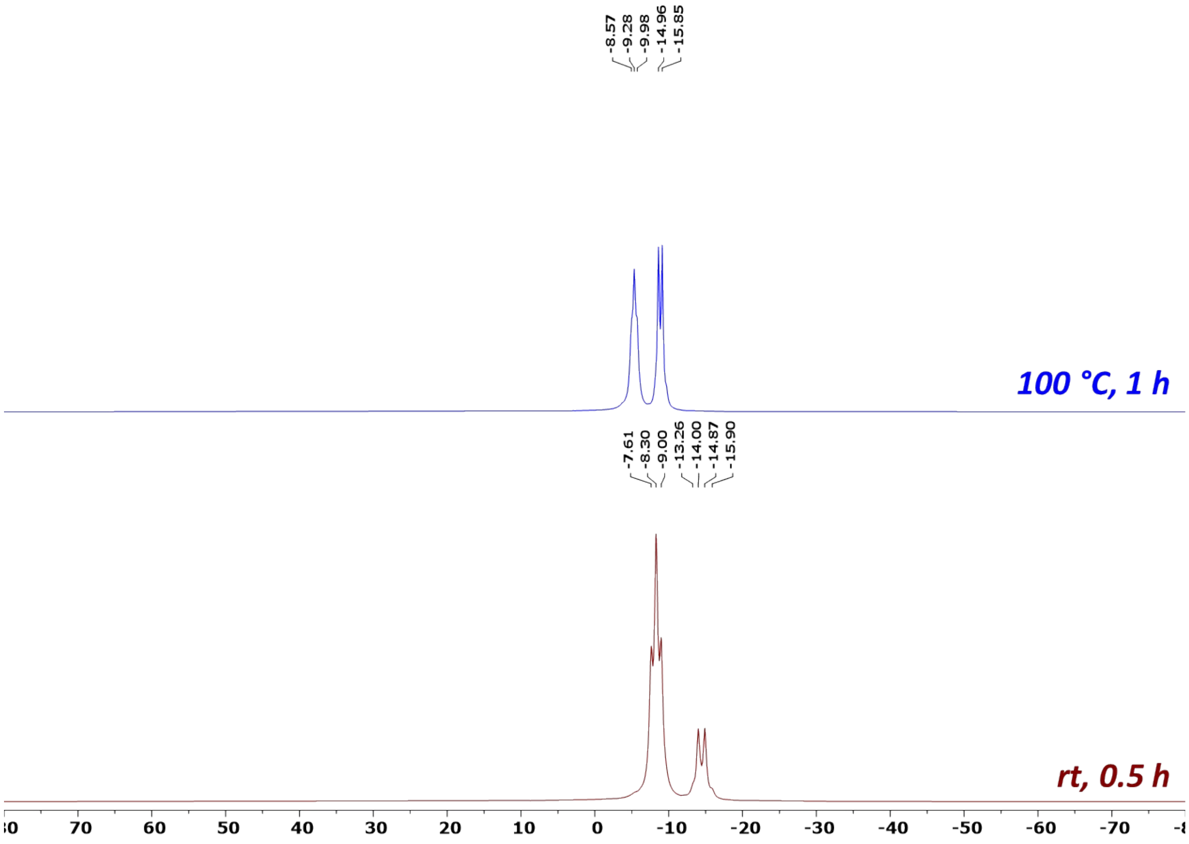

Figure S48: ^{11}B NMR spectrum (160 MHz, PhCl, 300 K) of the reaction between **7** and pyrazabole after 0.5 hour at room temperature (bottom red). ^{11}B NMR spectrum (160 MHz, PhCl, 300 K) of the reaction between **7** and pyrazabole after 1 hour at 100 °C (top blue).

After 0.5 hour at room temperature the ^{11}B NMR analysis revealed almost full conversion of the diiodo-pyrazabole **7** to the mono-iodo-pyrazabole **6**, evidenced by the presence of

a single doublet at δ_{11B} −14.4 and a triplet at −9.98. The triplet was in fact overlapped with the signal from the starting material pyrazabole, evidenced by the clear difference in intensity of the signals with the doublet. The remaining pyrazabole can be explained by the poor solubility of the diiodo-pyrazabole **7** staying out of solution. Therefore the solution was heated at 100 °C for 1 hour and full conversion of the pyrazabole and diiodo-pyrazabole **7** to the monoiodo-pyrazabole **6** was observed.

5.5.4 *Reaction between Diiodo-pyrazabole 7 and Et_3N (1 or 2 equiv.)*

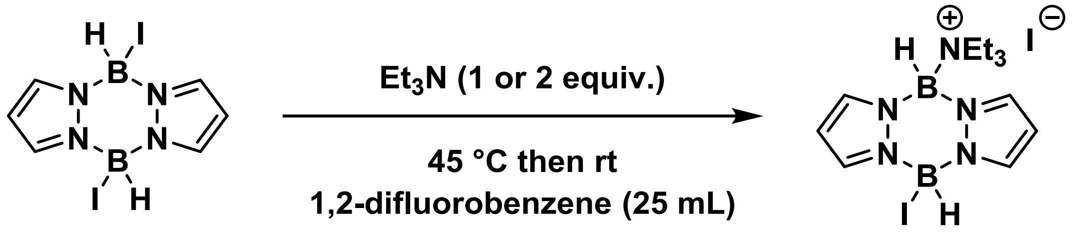

Diiodo-pyrazabole **7** (0.10 g, 0.24 mmol, 1.00 equiv.) was dissolved in 1,2-difluorobenzene (25 mL) and heated at 45 °C until all the solid dissolved in a J. Young's ampoule. The solution was cooled down to room temperature and Et_3N (34/68 µL, 0.24/0.48 mmol, 1/2 equiv.). The reaction was left stirring at room temperature for 5 minutes before NMR analysis.

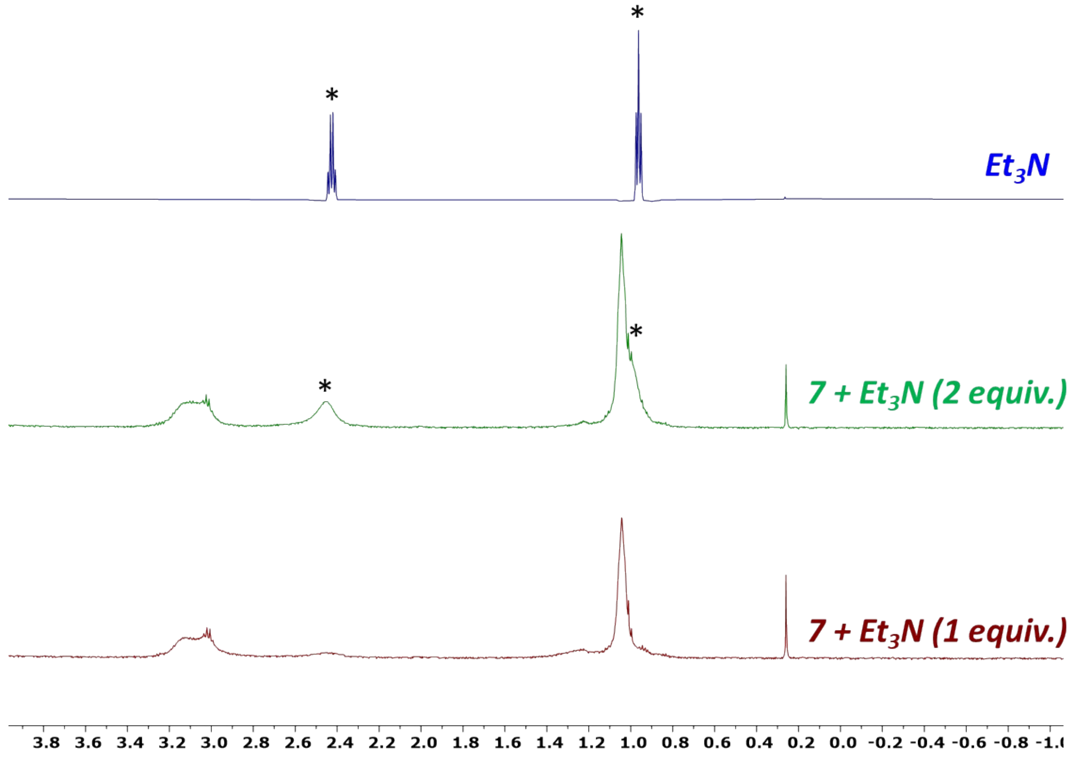

Figure S49: ^1H NMR spectrum (500 MHz, $C_6H_4F_2$, 300 K) of the reaction between diiodo-pyrazabole **7** (1equiv.) and Et_3N (1 equiv.) after 5 minutes, focussed on the alkyl region (bottom red). ^1H NMR spectrum (500 MHz, $C_6H_4F_2$, 300 K) of the reaction between diiodo-pyrazabole **7** (1 equiv.) and Et_3N (2 equiv.) after 5 minutes, focussed on the alkyl region (middle green). ^1H NMR spectrum (600 MHz, $C_6H_4F_2$, 300 K) of Et_3N (top blue).

^1H NMR analysis of the alkyl region revealed that stoichiometric or excess addition of Et_3N led to the formation of the same product. Identical deshielded signals from Et_3N (δ_{1H} = 3.06 and 1.04) were observed in both experiments (Figure S49, red and green),

highlighting coordination from the nitrogen atoms (aromatic region could not be analysed due to the presence of non-deuterated aromatic solvent). When 2 equivalents of Et_3N were added, new signals from Et_3N were observed (δ_{1H} = 2.45 and 0.99), which were in fact signals from free Et_3N after comparison with a Et_3N solution in 1,2-difluorobenzene (labelled * in Figure S49, blue and green spectra).

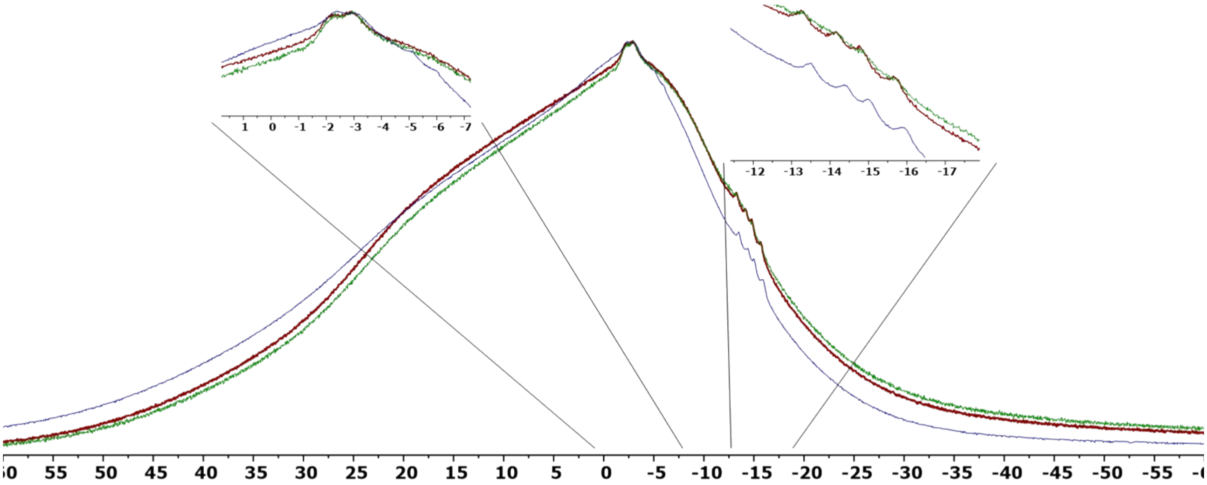

Figure S50: ^{11}B NMR spectrum (160 MHz, $C_6H_4F_2$, 300 K) of the reaction between diiodopyrazabole **7** (1 equiv.) and Et_3N (1 equiv.) after 5 minutes (red). ^{11}B NMR spectrum (160 MHz, $C_6H_4F_2$, 300 K) of the reaction between diiodo-pyrazabole **7** (1 equiv.) and Et_3N (2 equiv.) after 5 minutes (green). ^{11}B NMR spectrum (160 MHz, $C_6H_4F_2$, 300 K) of the isolated mono-cation **8** (blue).

^{11}B NMR analysis confirmed formation the same product in both stoichiometric and excess Et_3N addition (Figure S50, red and green), which was identified as the mono-cation **8** and later isolated (Figure S50, blue; See SI, Part 3.4 for synthesis of **8**).

5.5.5 Order of addition study

5.5.5.1 Base before substrate

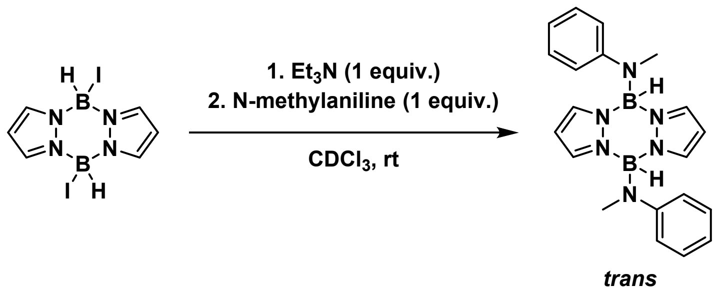

Diiodo-pyrazabole **7** (5 mg, 0.01 mmol, 1.00 equiv.) was dissolved in $CDCl_3$ in a J. Young's NMR tube. Et_3N (1.7 µL, 0.01 mmol, 1.00 equiv.) was added and the solution was left at room temperature for 20 minutes. N-methylaniline (1.3 µL, 0.01 mmol, 1.00 equiv.) was added and the solution was left at room temperature for 20 minutes before NMR analysis.

5.5.5.2 Substrate before base

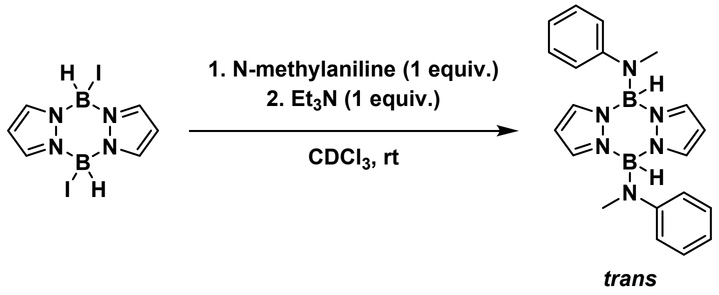

Diiodo-pyrazabole **7** (5 mg, 0.01 mmol, 1.00 equiv.) was dissolved in $CDCl_3$ in a J. Young's NMR tube. N-methylaniline (1.3 µL, 0.01 mmol, 1.00 equiv.) was added and the solution was left at room temperature for 20 minutes. Et_3N (1.7 µL, 0.01 mmol, 1.00 equiv.) was added and the solution was left at room temperature for 20 minutes before NMR analysis.

*5.5.5.3 NMR spectra of order of addition study: room temperature in situ observation of B-**5***

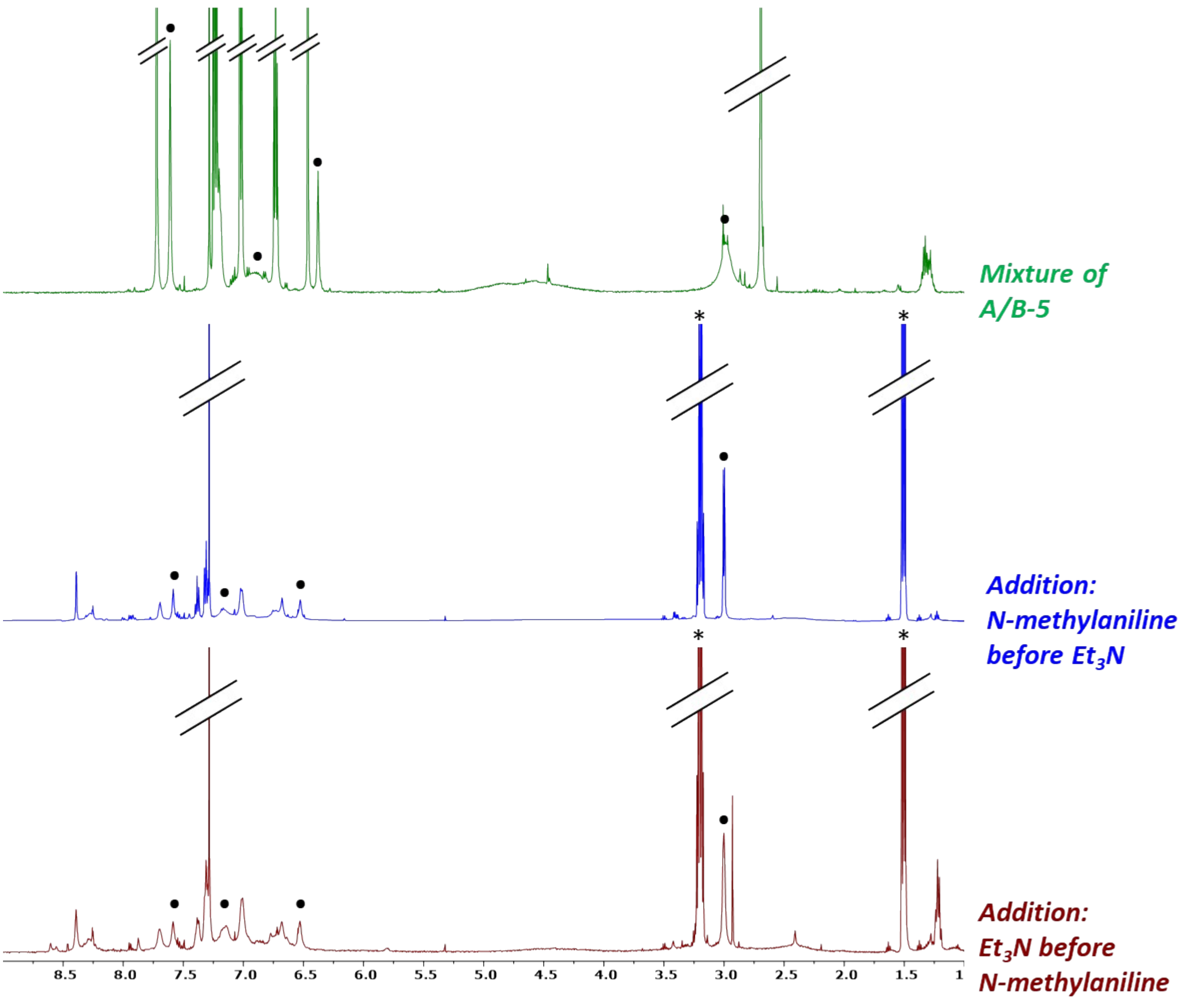

Figure S51: ^{1}H NMR spectrum (500 MHz, $CDCl_3$, 300 K) of the reaction between diiodo-pyrazabole **7**, Et_3N (added first) and N-methylaniline (bottom red). ^{1}H NMR spectrum (500 MHz, $CDCl_3$, 300 K) of the reaction between diiodo-pyrazabole **7**, N-methylaniline (added first) and Et_3N (middle blue). ^{1}H NMR spectrum (500 MHz, $CDCl_3$, 300 K) of a sample of *A*- and *B*-**5** (top green, *the spectrum has been scaled up, focussing on signals from the B-**5** which was the minor isomer*).

^{1}H NMR analysis showed that the order of addition led to the same outcome, with almost identical spectra (Figure S51, red and blue spectra). In addition to the signals from the protonated Et_3N (labelled *, Figure S51), signals from the *B*-**5** were observed *in situ* for both order of addition experiments when compared with an isolated sample of *A*- and *B*-**5** (labelled •, Figure S51). Reactions with the diiodo-pyrazabole **7** system not being clean, led to a complex aromatic region in the *in situ* systems, due to this not all the signals could clearly be seen by ^{1}H NMR spectroscopy.

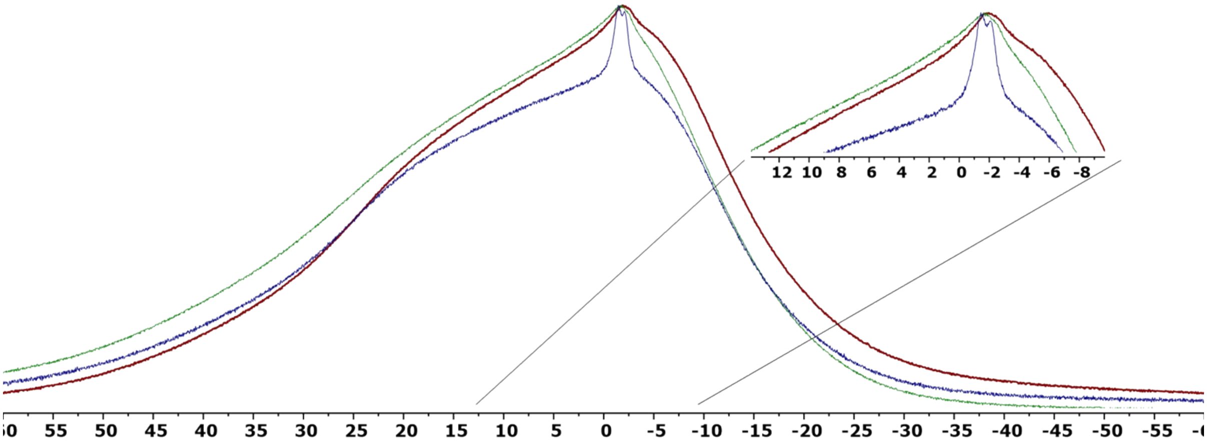

Figure S52: ^{11}B NMR spectrum (160 MHz, $CDCl_3$, 300 K) of the reaction between diiodo-pyrazabole **7**, Et_3N (added first) and N-methylaniline (red). ^{11}B NMR spectrum (160 MHz, $CDCl_3$, 300 K) of the reaction between diiodo-pyrazabole **7**, N-methylaniline (added first) and Et_3N (green). ^{11}B NMR spectrum (160 MHz, $CDCl_3$, 300 K) of a sample of *A*- and *B*-**5** (blue).

For both order of addition reactions, ^{11}B NMR analysis showed only one small signal at $\delta_{11B} \approx -1.7$ (Figure S52, red and green), confirming that the order of addition had no impact on the reaction. These observed signals were found at the same chemical shift to the one from *A*- and *B*-**5** (Figure S52, blue), highlighting the immediate formation of the intermediate *B*-**5** at room temperature during the reaction.

5.5.6 BDB attempt at 70 ˚C: in situ observation of B-5

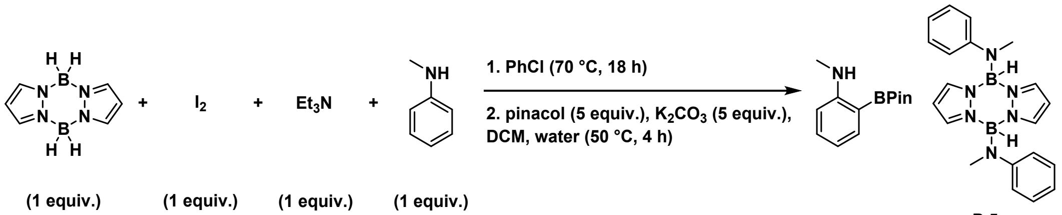

Pyrazabole (47 mg, 0.30 mmol, 1.00 equiv.) was dissolved in PhCl (2 mL) in a J. Young's ampoule. Iodine (76 mg, 0.30 mmol, 1.00 equiv.) was added at room temperature and the solution was stirred for 2 hours. Et_3N (42 µL, 0.30 mmol, 1.00 equiv.) and N-methylaniline (33 µL, 0.30 mmol, 1.00 equiv.) were successively added to the solution. The reaction mixture was heated at 70 °C for 18 hours. The solution was cooled down to room temperature. Pinacol (177 mg, 1.50 mmol, 5.00 equiv.), K_2CO_3 (207 mg, 1.50 mmol, 5.00 equiv.), DCM (4 mL) and water (4 mL) were sequentially added. The resulting biphasic mixture was heated at 50 °C for 4 hours under vigorous stirring. The product was extracted with pentane (4 x 10 mL). The combined organic phases were washed with water (4 x 10 mL), dried over $MgSO_4$, and filtered. Volatiles were removed *in vacuo* affording a mixture of products described below.

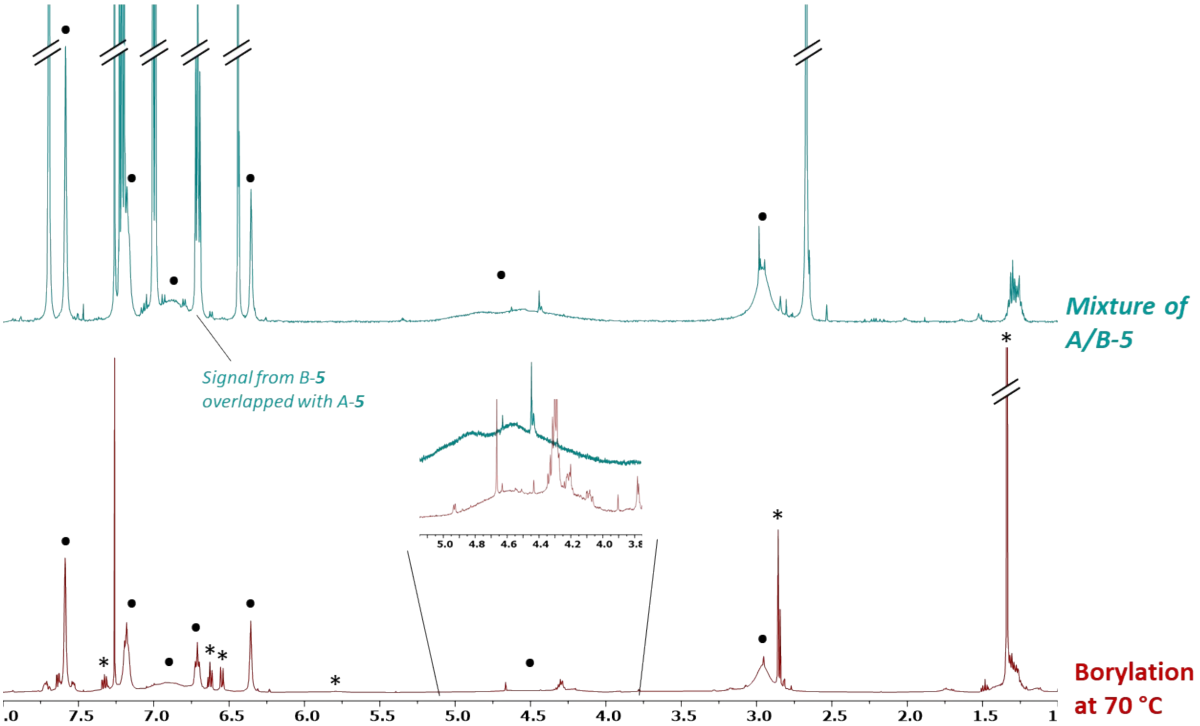

Figure S53: ^1H NMR spectrum (500 MHz, $CDCl_3$, 300 K) of the mixture of products obtained from the BDB reaction at 70 ˚C (bottom red). ^1H NMR spectrum (500 MHz, $CDCl_3$, 300 K) of a sample of *A*- and *B*-**5** (top blue, *the spectrum has been scaled up, focussing on signals from the B-***5** *which was the minor isomer*).

The borylation of N-methylaniline using diiodo-pyrazabole **7** and Et_3N carried out at lower temperature (70 °C) led to a mixture containing traces of the borylated N-methylaniline **3a** (labelled *, Figure S53, bottom red) and the intermediate *B*-**5** (labelled •, Figure S53, bottom red), confirmed by comparing with a sample from an isolated mixture of *A*- and *B*-**5** (labelled •, Figure S53, top blue).

As **5** was not observed when the BDB reaction was carried out under the optimised conditions (100 °C heating instead of 70 °C), this confirmed that the intermediate *B*-**5** is formed *in situ* and converted into the borylated species during the reaction.

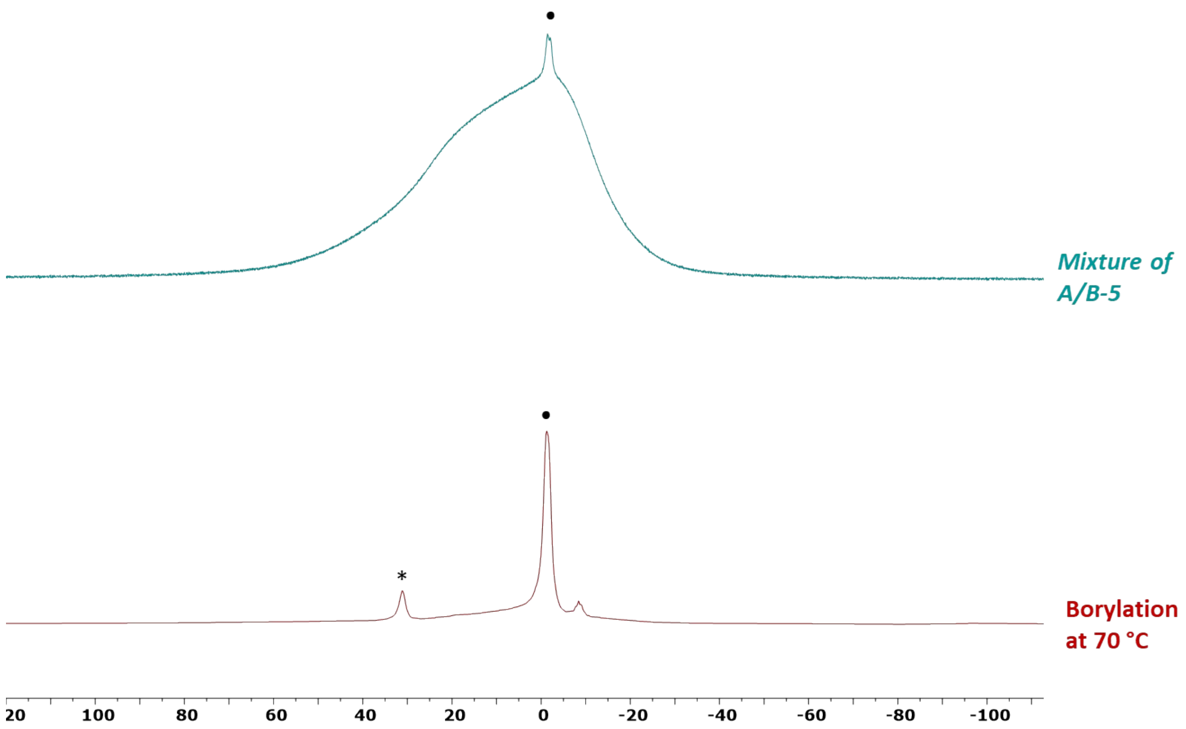

Figure S54: ^{11}B NMR spectrum (160 MHz, $CDCl_3$, 300 K) of the mixture of products obtained from the BDB reaction at 70 °C (bottom red). ^{11}B NMR spectrum (160 MHz, $CDCl_3$, 300 K) of a sample of *A*- and *B*-**5** (top blue).

^{11}B NMR was in agreement with ^{1}H NMR based conclusions, showing that borylation at lower temperature was effectively giving the intermediate *B*-**5** (labelled •, Figure S54, bottom red and top blue) as the major species, along with trace of the borylated N-methylaniline **3a** (labelled *, Figure S54, bottom red).

5.5.7 *In situ observation of borylated intermediate [2]I*

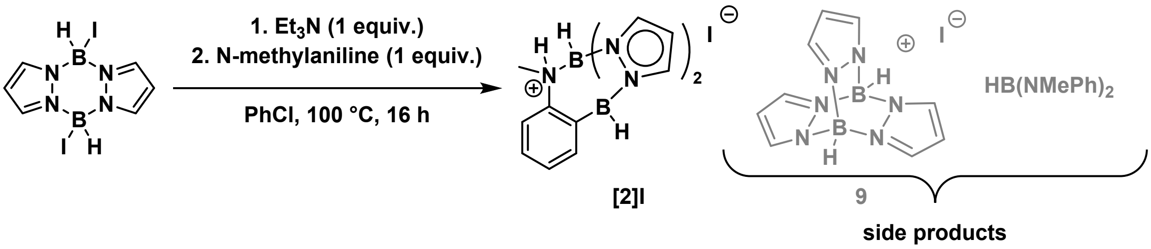

Diiodo-pyrazabole **7** (0.123 g, 0.30 mmol, 1.00 equiv.) was dissolved in PhCl in a J. Young's ampoule. Et_3N (42 µL, 0.30 mmol, 1.00 equiv.) and N-methylaniline (33 µL, 0.30 mmol, 1.00 equiv.) were successively added to the solution. The reaction mixture was heated at 100 °C for 16 hours. Volatiles were removed *in vacuo* and the crude mixture was analysed by NMR.

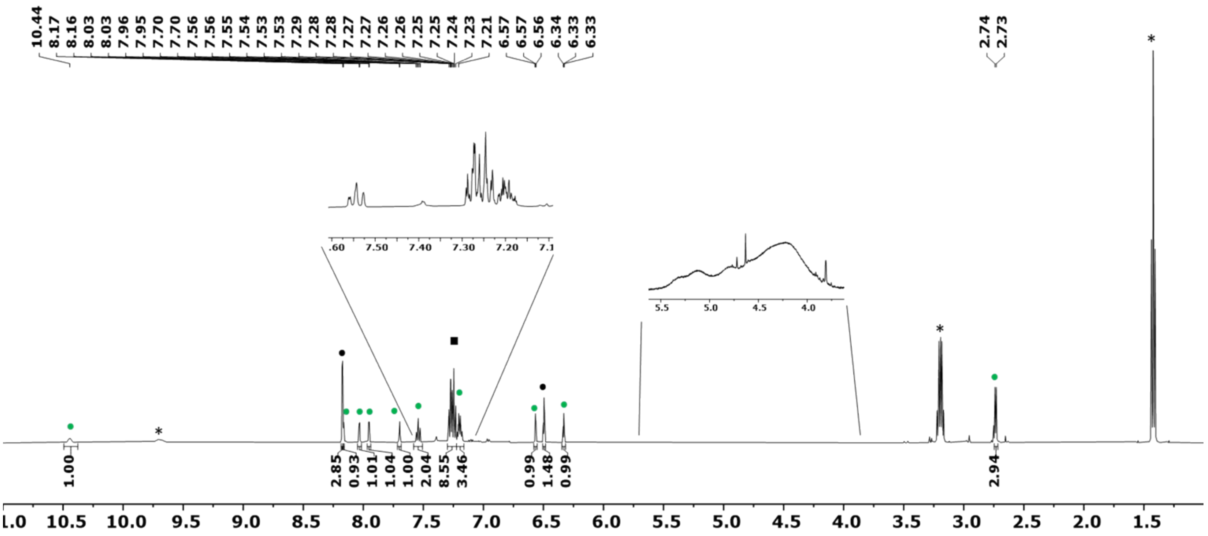

Figure S55: ^1H NMR spectrum (500 MHz, $CDCl_3$, 300 K) of the crude borylation reaction mixture after 100 °C for 16 hours.

^1H NMR analysis showed the protonated base Et_3NHI (labelled *, Figure S55), a pyrazabole type unit containing no N-methylaniline moiety (labelled •, Figure S55), which was later identified as the side product **9** (See SI, Parts 5.5.8 and 5.5.9) and a new set of signals which were consistent with **[2]I** (labelled •, Figure S55). Relative integration showed a ratio of 2:1 for **[2]I** : **9**.

In the aromatic region, extra signals were observed from another species (labelled ■, Figure S55). These were assigned to the side product $HB(NMePh)_2$ after analysis of the ^{11}B NMR (see below, Figure S56).

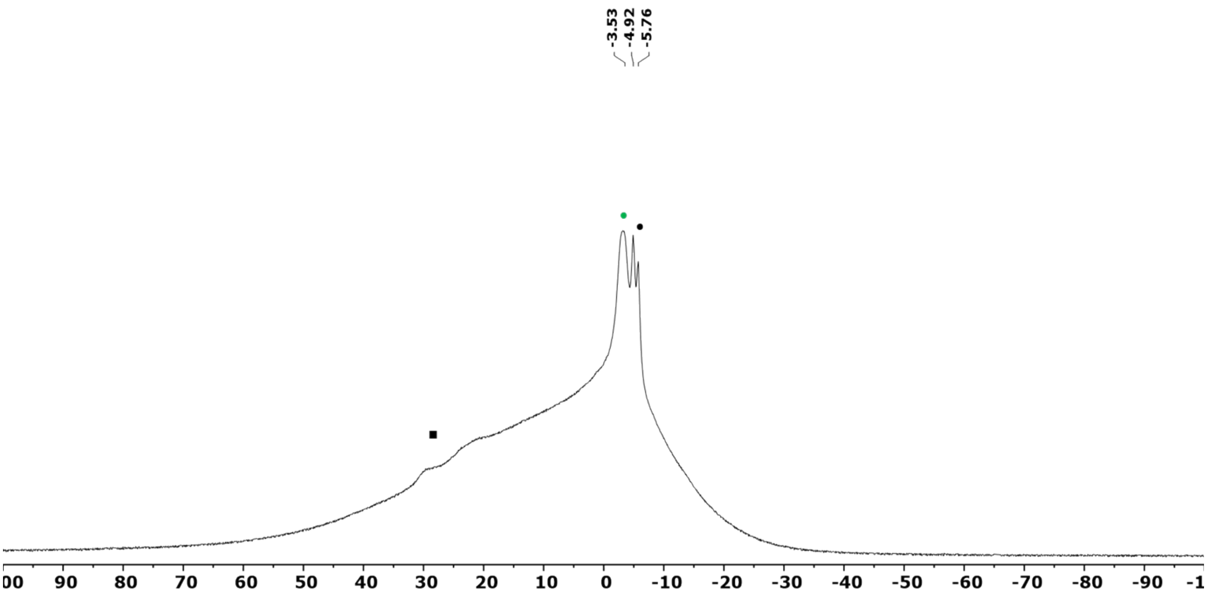

Figure S56: ^{11}B NMR spectrum (160 MHz, $CDCl_3$, 300 K) of the crude BDB reaction mixture after 100 °C for 16 hours.

^{11}B NMR analysis showed a pyrazabole unit signal (δ_{11B} −5.3, d, $^{1}J_{BH}$ = 133 Hz: labelled •, Figure S56), which was later identified as the by-product **9** (See SI, Parts 5.5.8 and 5.5.9). In addition, a broad signal around δ_{11B} 29.0 ($^{1}J_{BH}$ ≈ 126 Hz, the low intensity of the signal made the exact measurement difficult) was assigned to decomposition by-product $HB(NMePh)_2$ (labelled ■, Figure S56) by comparison with literature (δ_{11B} 29.6, $^{1}J_{BH}$ = 122 Hz, in toluene-d8).[S13] Finally the signal at δ_{11B} −3.5 (s br) was assigned to the intermediate **[2]I** (labelled •, Figure S56).. This signal was found very close to the analogue **[2]NTf$_2$** (δ_{11B} −3.1, s br).

*5.5.7.1 NMR data assigned to intermediate **[2]I***

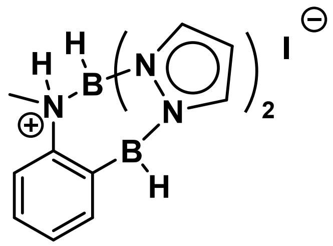

^{1}H NMR* (500 MHz, $CDCl_3$, 300 K) δ 10.44 (d br, $^3J_{HH}$ = 4.5 Hz, N*H*, 1H), 8.17 (d, $^3J_{HH}$ = 2.3 Hz, NC*H*, 1H), 8.03 (d, $^3J_{HH}$ = 2.5 Hz, NC*H*, 1H), 7.95 (d, $^3J_{HH}$ = 2.3 Hz, NC*H*, 1H), 7.70 (d, $^3J_{HH}$ = 2.3 Hz, NC*H*, 1H), 7.54 (m, $C^{Ar}H$, 2H), 7.30-7.20 (m, $C^{Ar}H$, 2H),** 6.57 (t, $^3J_{HH}$ = 2.4 Hz, CC*H*, 1H), 6.33 (t, $^3J_{HH}$ = 2.4 Hz, CC*H*, 1H), 2.73 (d, $^3J_{HH}$ = 5.6 Hz, NCH_3, 3H).

*Due to the presence of multiple B-H containing species, integration /assignment in the B-H region was not possible.

**This signal was overlapped with another species which was assigned to a decomposition by-product $HB(NMePh)_2$ (labelled ■, Figure S55).

^{11}B NMR (160 MHz, $CDCl_3$, 300 K) δ –3.5 (s br, *B*H, 2B).

5.5.8 Generation of compound 9

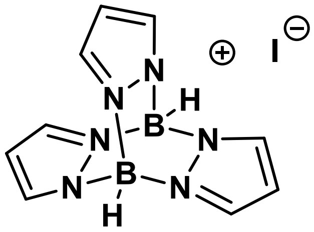

Preparation 1:

Diiodo-pyrazabole **7** (11 mg, 0.03 mmol, 1.00 equiv.) and di-N-methylaniline-pyrazabole **5** (10 mg, 0.03 mmol, 1.00 equiv.) were dissolved in PhCl (1 mL) and heated for 16 hours at 100 °C in a sealed J. Young's NMR tube. Suitable crystals for X-ray analysis formed upon heating.

Preparation2:

diiodo-pyrazabole **7** (11 mg, 0.03 mmol, 1.00 equiv.) and di-N-methylaniline-pyrazabole **5** (10 mg, 0.03 mmol, 1.00 equiv.) were dissolved in PhCl (1 mL) and heated over 72 hours at 60 °C in a sealed J. Young's NMR tube. The formed colourless crystals were isolated by filtration of the supernatant solution and washed with PhCl (2 x 0.5 mL), affording pure colourless crystals of **9**, for complete NMR and mass spectrometry characterization (yield not recorded).

^{1}H NMR (500 MHz, $CDCl_3$, 300K) δ 8.24 (d, $^{3}J_{HH}$ = 2.5 Hz, NC*H*, 6H), 6.51 (t, $^{3}J_{HH}$ = 2.5 Hz, CC*H*, 3H), 5.65-4.53 (m br, B*H*, 2H).

^{1}H {^{11}B} NMR (500 MHz, $CDCl_3$, 300K) δ 8.24 (d, $^{3}J_{HH}$ = 2.5 Hz, NC*H*, 6H), 6.51 (t, $^{3}J_{HH}$ = 2.5 Hz, CC*H*, 3H), 5.08 (s br, B*H*, 2H).

^{11}B NMR (160 MHz, $CDCl_3$, 300 K) δ –5.26 (d, $^{1}J_{BH}$ = 137 Hz, *B*H).

^{11}B {^{1}H} NMR (160 MHz, $CDCl_3$, 300 K) δ –5.28 (s, *B*H).

^{13}C {^{1}H} NMR (126 MHz, $CDCl_3$, 300K) δ 138.5 (s, N*C*H, 6C), 108.3 (s, C*C*H, 3C).

Mass Spectrum: HRMS (EI+) m/z calculated for $C_9H_{11}B_2N_6$: 225.12258; Found: 225.12298.

5.5.8.1 NMR spectra of ***9***

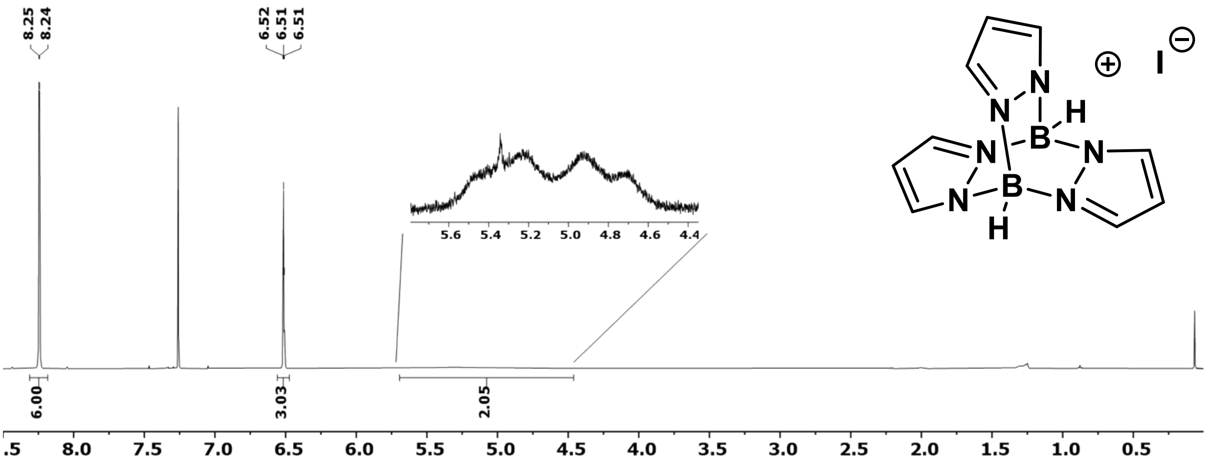

Figure S57: ^{1}H NMR spectrum (500 MHz, $CDCl_3$, 300K) of **9**.

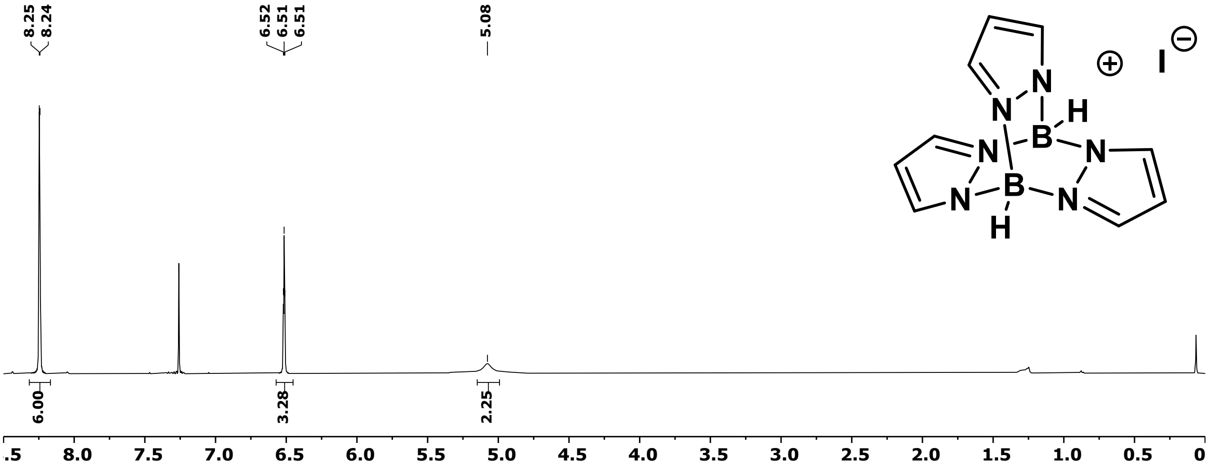

Figure S58: ^{1}H {^{11}B} NMR spectrum (500 MHz, $CDCl_3$, 300K) of **9**.

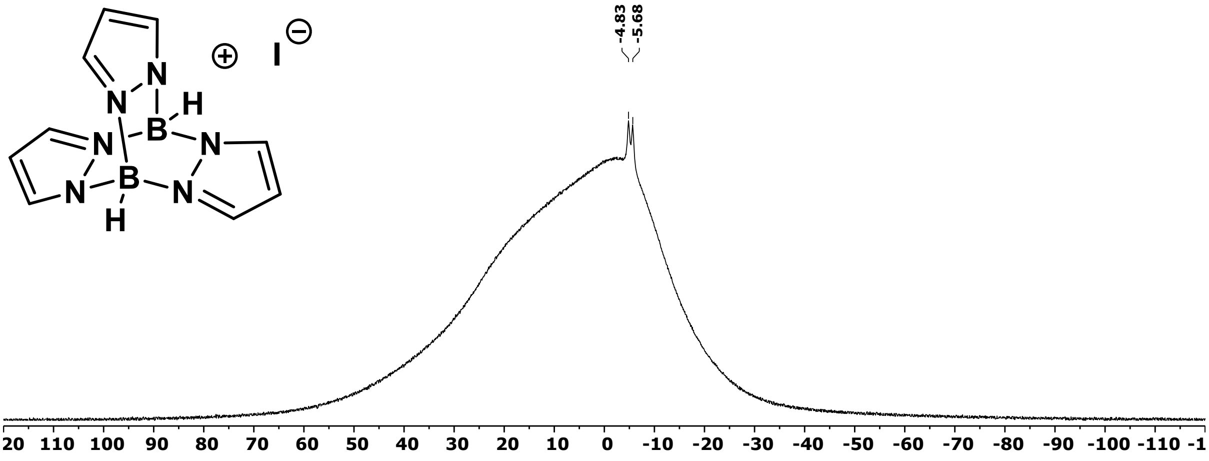

Figure S59: ^{11}B NMR spectrum (160 MHz, $CDCl_3$, 300 K) of **9**.

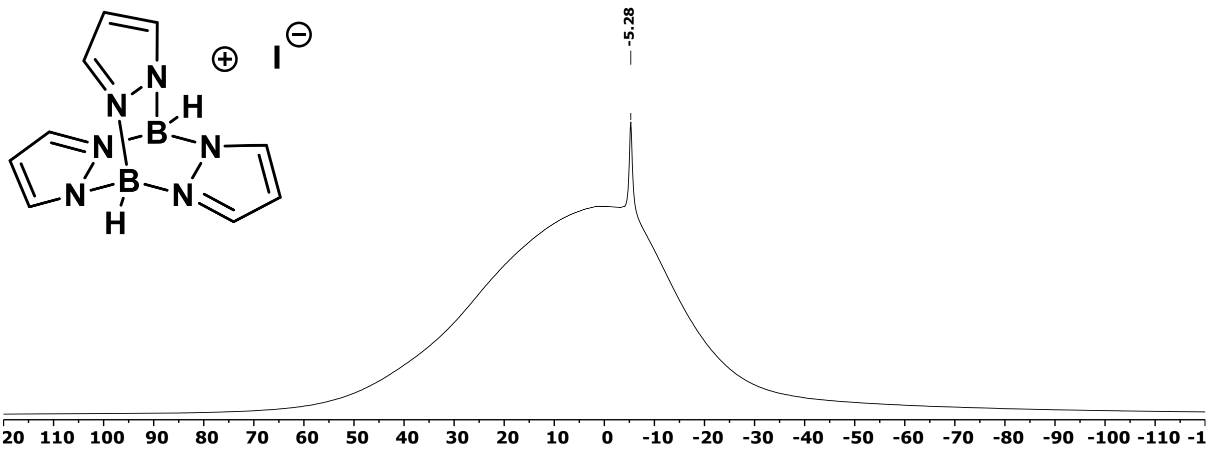

Figure S60: $^{11}B\ \{^{1}H\}$ NMR spectrum (160 MHz, $CDCl_3$, 300 K) of **9**.

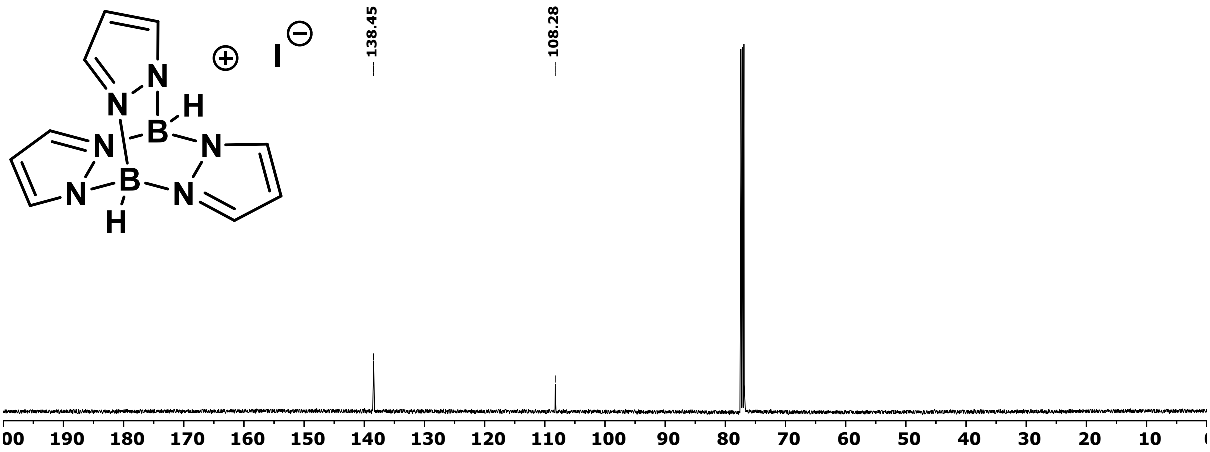

Figure S61: $^{13}C\ \{^{1}H\}$ NMR spectrum (126 MHz, $CDCl_3$, 300K) of **9**.

5.5.9 In situ formation of compound 9

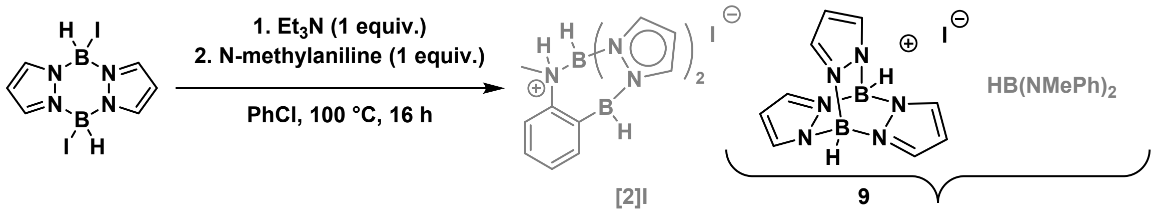

Diiodo-pyrazabole **7** (20 mg, 0.05 mmol, 1.00 equiv.) was dissolved in PhCl (2 mL). Et_3N (7 µL, 0.05 mmol, 1.00 equiv.) and N-methylaniline (6 µL, 0.05 mmol, 1.00 equiv.) were successively added. The solution was heated at 100 °C for 20 hours. The solution was cooled down to 0 °C and a white solid precipitated out of solution. Filtration allowed isolation of a white residue which was a mixture of protonated base [Et_3NH]I and compound **9**.

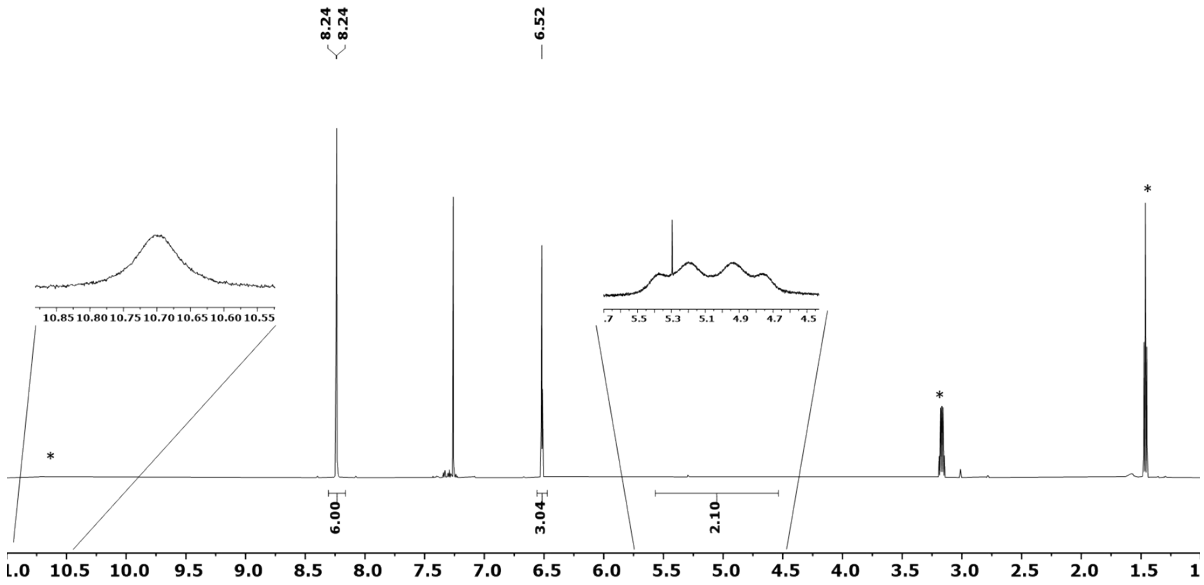

Figure S62: ^{1}H NMR spectrum (600 MHz, $CDCl_3$, 300 K) of the isolated white residue from a BDB reaction, containing a mixture of [Et_3NH]I (labelled *) and compound **9**.

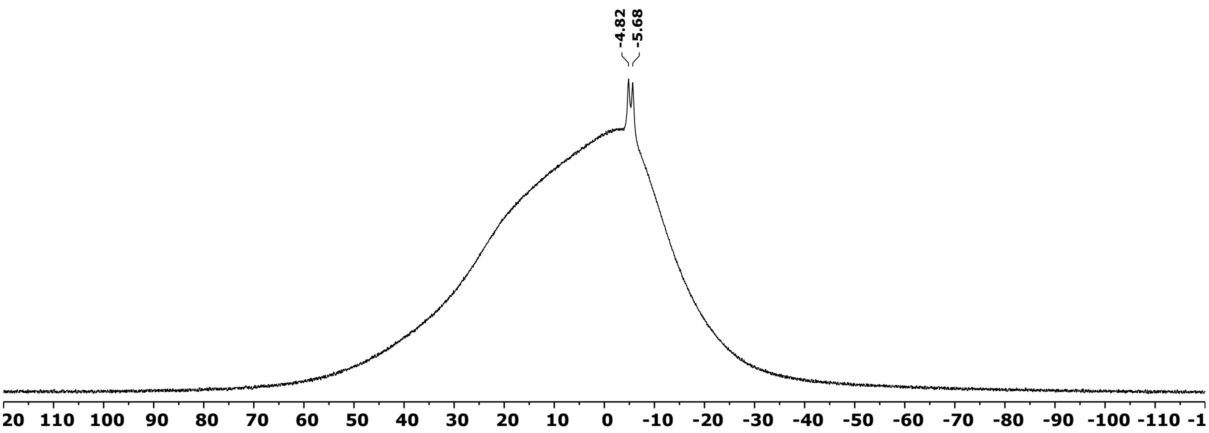

Figure S63: ^{11}B NMR spectrum (160 MHz, $CDCl_3$, 300 K) of the isolated white residue from a BDB reaction, containing a mixture of $[Et_3NH]I$ and compound **9**.

^{1}H and ^{11}B NMR spectra showed identical signals to the isolated compound **9** (See SI, Part 5.5.8), confirming the formation of **9** as a side product during the BDB reaction of N-methylaniline with diiodo-pyrazabole **7**.

5.5.10 Reaction between diiodo-pyrazabole 7 and [Et$_3$NH]NTf$_2$

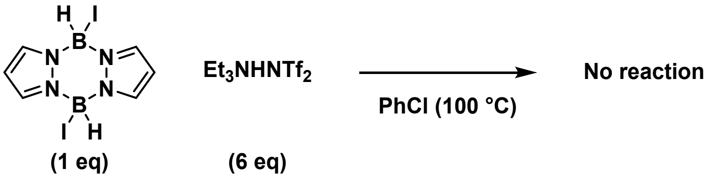

Diiodo-pyrazabole **7** (10 mg, 0.02 mmol, 1.00 equiv.) and Et_3NHNTf_2 (46 mg, 0.12 mmol, 6.00 equiv.) were dissolved in PhCl (0.5 mL) in a J. Young's NMR tube and heated at 100 °C for 24 hours.

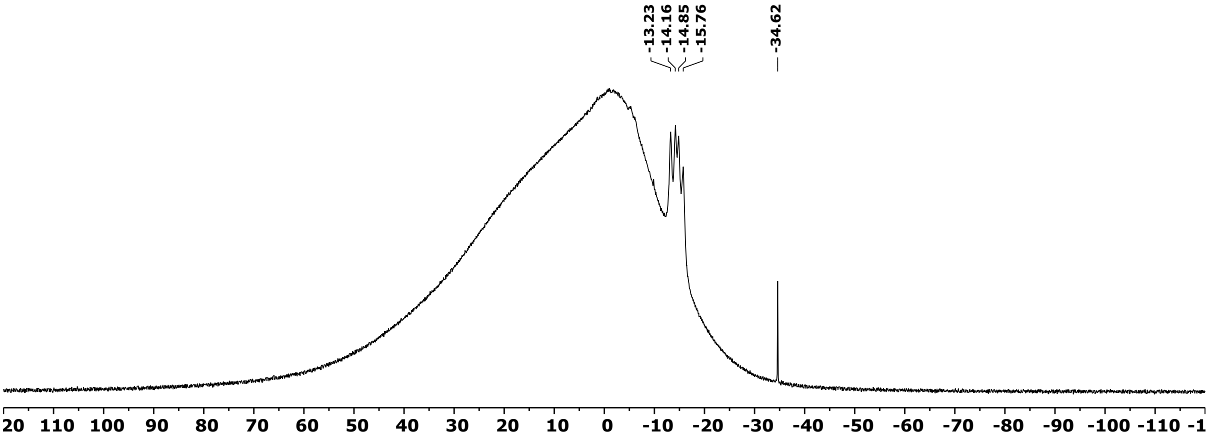

Figure S64: ^{11}B NMR spectrum (160 MHz, PhCl, 300 K) of the *in situ* reaction between diiodo-pyrazabole **7** (1 equiv.) and $[Et_3NH]NTf_2$ (6 equiv.) after heating at 100 °C for 24 hours.

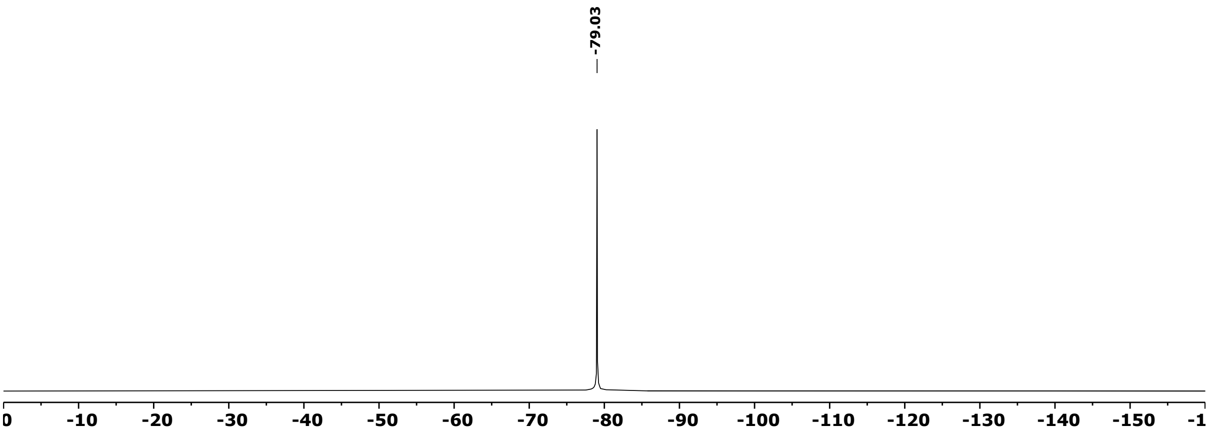

Figure S65: ^{19}F NMR spectrum (471 MHz, PhCl, 300 K) of the *in situ* reaction between diiodo-pyrazabole **7** (1 equiv.) and $[Et_3NH]NTf_2$ (6 equiv.) after heating at 100 °C for 24 hours.

After heating at 100 °C for 24 hours, no exchange was observed by ^{11}B or ^{19}F NMR spectroscopy between the iodide and the triflimide moieties. Only starting materials were seen along with a small amount of decomposition in ^{11}B NMR (δ_{11B} −34.6, s).

5.6 Hygroscopicity check of $[Et_3NH]NTf_2$

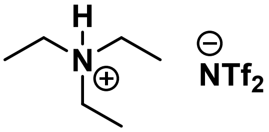

$[Et_3NH]NTf_2$ (20 mg) was taken out of the glovebox and exposed to air for 15 min before being stored in a vial for 20 hours, without nitrogen purging or parafilm sealing. The material was then NMR in dry $CDCl_3$ to check the stability and hygroscopicity of $[Et_3NH]NTf_2$ when exposed to moisture and air.

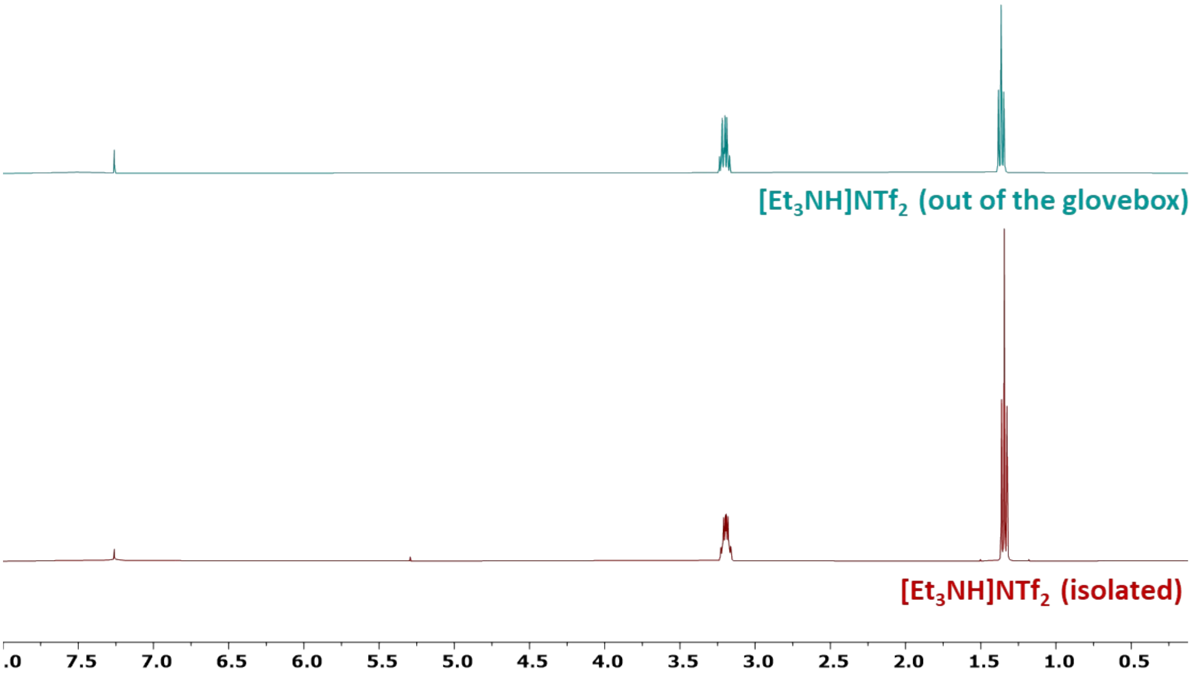

Figure S66: ^{1}H NMR spectra (400 MHz, $CDCl_3$, 300 K) of an isolated sample of $[Et_3NH]NTf_2$ stored under inert conditions (bottom red) and a sample of $[Et_3NH]NTf_2$ stored without air or moisture avoidance (top blue).

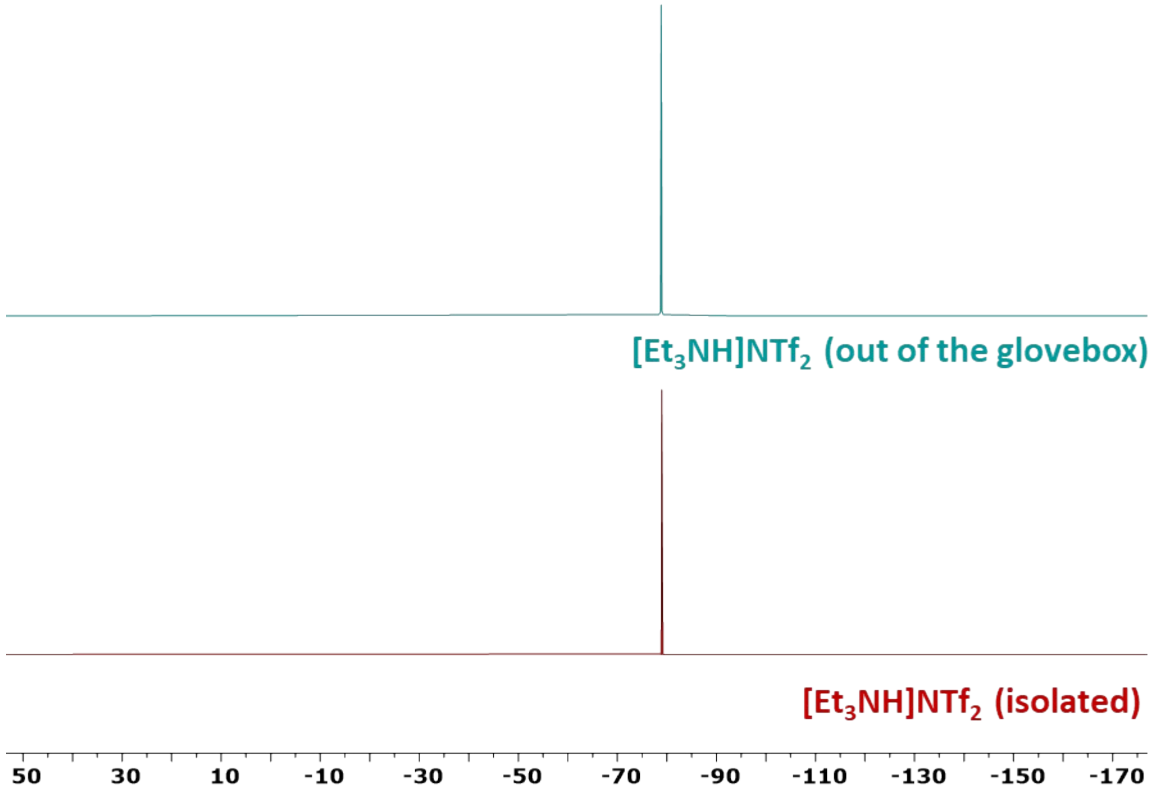

Figure S67: ^{19}F NMR spectrum (376 MHz, $CDCl_3$, 300 K) of a sample of Et_3NHNTf_2 stored under inert conditions (bottom red). ^{19}F NMR spectrum (471 MHz, $CDCl_3$, 300 K) of a sample of Et_3NHNTf_2 stored without air or moisture avoidance (top blue).

No visible changes were observed by ^1H and ^{19}F NMR spectroscopy between the two samples of $[Et_3NH]NTf_2$; asserting for the air and moisture complete stability and low hygroscopicity of $[Et_3NH]NTf_2$.

6 BDB comparison: 7 *vs* 1 *vs* combined 7/catalytic 1

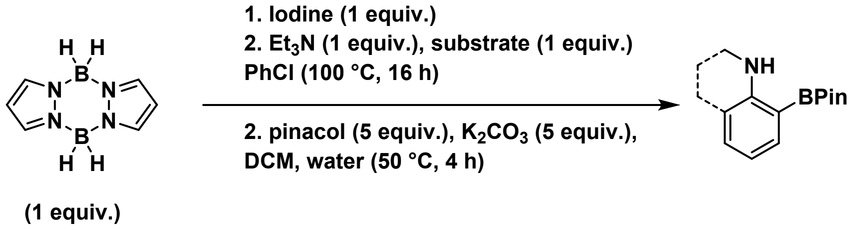

Conditions A: Pyrazabole (47 mg, 0.30 mmol, 1.00 equiv.) was dissolved in solvent (1.5-2 mL) in a J. Young's ampoule. Iodine (76 mg, 0.30 mmol, 1.00 equiv.) was added at room temperature and the solution was stirred for 2 hours. Et_3N (42 µL, 0.30 mmol, 1.00 equiv.) and substrate (0.30 mmol, 1.00 equiv.) were successively added to the solution. The reaction mixture was heated at 100 °C for 16 hours. The solution was cooled down to room temperature. Pinacol (177 mg, 1.50 mmol, 5.00 equiv.), K_2CO_3 (207 mg, 1.50 mmol, 5.00 equiv.), DCM (2.8 mL) and water (2.8 mL) were sequentially added. The resulting biphasic mixture was heated at 50 °C for 4 hours with vigorous stirring. The product was extracted with pentane (4 x 10 mL). The combined organic phases were washed with water (4 x 10 mL), dried over $MgSO_4$, and filtered. Volatiles were removed *in vacuo*.

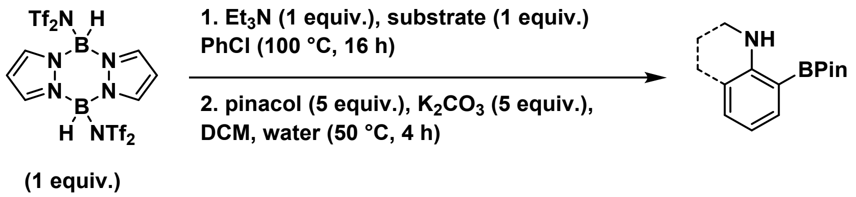

Conditions B: Di-NTf_2-pyrazabole **1** (180/0.215 mg, 0.25/0.30 mmol, 1.00 equiv.), Et_3N (35/42 µL, 0.25/0.30 mmol, 1.00 equiv.) and substrate (0.25/0.30 mmol, 1.00 equiv.) were combined in PhCl (2 mL) in a J. Young's ampoule and heated at 100 °C for 24 hours. The solution was cooled down to room temperature. Pinacol (147/177 mg, 1.25/1.5 mmol, 5.00 equiv.), K_2CO_3 (173/207 mg, 1.25/1.50 mmol, 5.00 equiv.), DCM (2.3/2.8 mL) and water (2.3/2.8 mL) were sequentially added. The resulting biphasic mixture was heated at 50 °C for 16 hours with vigorous stirring. The product was extracted with pentane (4 x 10 mL). The combined organic phases were washed with water (4 x 10 mL), dried over $MgSO_4$, and filtered. Volatiles were removed *in vacuo*.

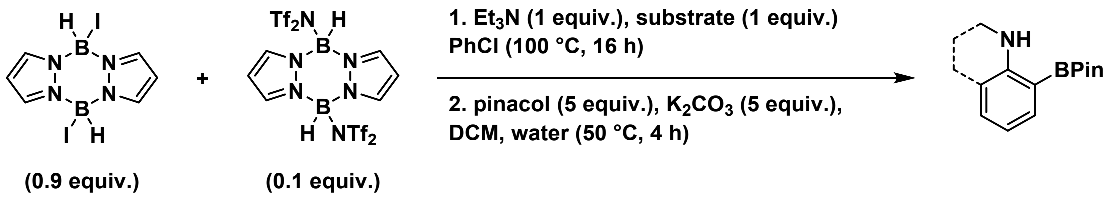

Conditions C: Diiodo-pyrazabole **7** (111 mg, 0.27 mmol, 0.90 equiv.) and di-NTf_2-pyrazabole **1** (22 mg, 0.03 mmol, 0.10 equiv.) were dissolved in PhCl (2 mL) at room temperature in a J. Young's ampoule. Et_3N (42 µL, 0.30 mmol, 1.00 equiv.) and substrate (0.30 mmol, 1.00 equiv.) were successively added to the solution. The reaction mixture was heated at 100 °C for 16 hours. The solution was cooled down to room temperature. Pinacol (177 mg, 1.50 mmol, 5.00 equiv.), K_2CO_3 (207 mg, 1.50 mmol, 5.00 equiv.), DCM (2.8 mL) and water (2.8 mL) were sequentially added. The resulting biphasic mixture was heated at 50 °C for 4 hours with vigorous stirring. The product was extracted with pentane (4 x 10 mL). The combined organic phases were washed with water (4 x 10 mL), dried over $MgSO_4$, and filtered. Volatiles were removed *in vacuo*.

Entry	Substrate	Conditions	Yield
1	N-methylaniline	A	47
2	N-methylaniline	B	65
3	N-methylaniline	C	63
4	Tetrahydroquinoline	A	49[a]
5	Tetrahydroquinoline	B	71[b]
6	Tetrahydroquinoline	C	76[b]

Table S3: Yield comparison of the Conditions A, B and C tried on 2 substrates. [a]Product was contaminated with significant amount of tetrahydroquinoline. [b]Product was contaminated with trace of tetrahydroquinoline.

7 Control reactions

7.1 BDB of N-methylaniline attempt from pyrazabole with catalytic $[Et_3NH]NTf_2$

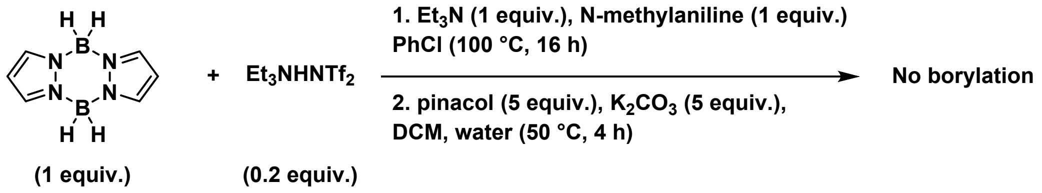

Pyrazabole (47 mg, 0.30 mmol, 1.00 equiv.) and $[Et_3NH]NTf_2$ (23 mg, 0.06 mmol, 0.20 equiv.) were dissolved in PhCl (2 mL) in a J. Young's ampoule. Et_3N (42 µL, 0.30 mmol, 1.00 equiv.) and N-methylaniline (33 µL, 0.30 mmol, 1.00 equiv.) were successively added to the solution. The reaction mixture was heated at 100 °C for 16 hours. The solution was cooled down to room temperature. Pinacol (177 mg, 1.50 mmol, 5.00 equiv.), K_2CO_3 (207 mg, 1.50 mmol, 5.00 equiv.), DCM (2.8 mL) and water (2.8 mL) were sequentially added. The resulting biphasic mixture was heated at 50 °C for 4 hours with vigorous stirring. The product was extracted with pentane (4 x 10 mL). The combined organic phases were washed with water (4 x 10 mL), dried over $MgSO_4$, and filtered. Volatiles were removed *in vacuo*. No borylated product **3a** was observed.

7.2 Hydrogen formation assessment

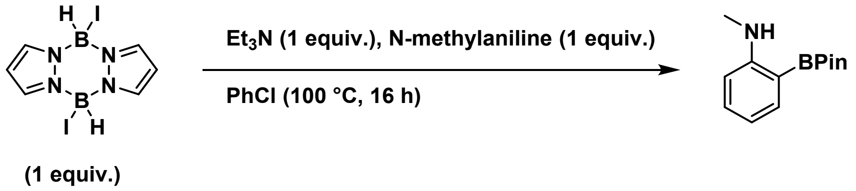

Diiodo-pyrazabole **7** (10 mg, 0.02 mmol, 1.00 equiv.) was dissolved in PhCl (1 mL) in a J. Young's NMR tube. Et_3N (4 µL, 0.02 mmol, 1.00 equiv.) and N-methylaniline (3 µL, 0.02 mmol, 1.00 equiv.) were added successively. The solution was heated at 100 °C for 16 hours.

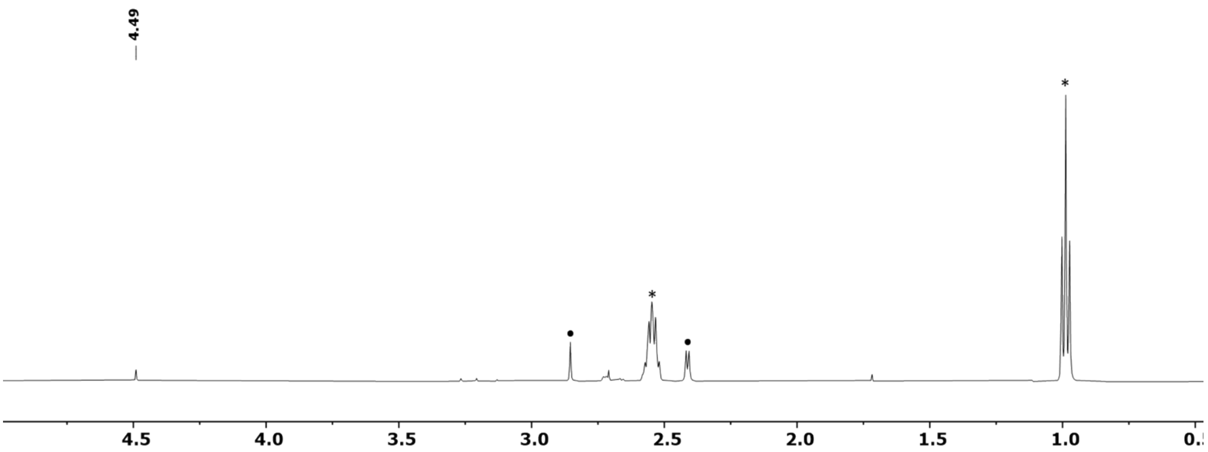

Figure S68: ^{1}H NMR spectrum (500 MHz, PhCl, 300 K) of the *in situ* BDB reaction of N-methylaniline using diiodo-pyrazabole **7** and Et_3N.

After heating at 100 °C for 16 hours, only traces of H_2 were observed by ^{1}H NMR spectroscopy (δ_{1H} 4.49, Figure S68). The small relative intensity of H_2 compared with peaks from [Et_3NH]I (labelled *, Figure S68) and from N-methylaniline or **[2]I** (both labelled •, Figure S68) confirmed that the borylation was likely to not be occurring through H_2 elimination. This was in accordance with the rest of the mechanistic studies. Similar reactions that do proceed with H_2 evolution (under the same scale / no. of mol. of H_2 produced give a much more intense H_2 signal in the same solvent).

8 BDB with alternative systems

8.1 Diiodo-pyrazabole + Cat. $LiNTf_2$

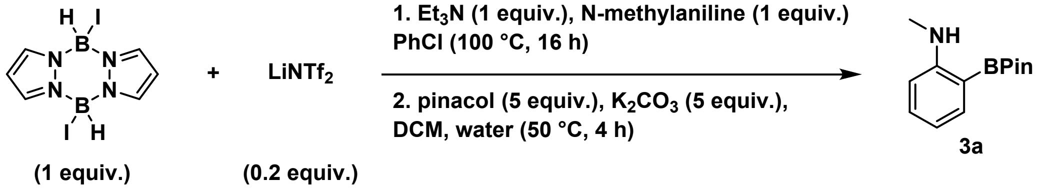

Diiodo-pyrazabole **7** (123 mg, 0.30 mmol, 1.00 equiv.) and $LiNTf_2$ (17 mg, 0.06 mmol, 0.20 equiv.) were dissolved in PhCl (2 mL) at room temperature in a J. Young's ampoule. Et_3N (42 µL, 0.30 mmol, 1.00 equiv.) and N-methylaniline (33 µL, 0.30 mmol, 1.00 equiv.) were successively added to the solution. The reaction mixture was heated at 100 °C for 16 hours. The solution was cooled down to room temperature. Pinacol (177 mg, 1.50 mmol, 5.00 equiv.), K_2CO_3 (207 mg, 1.50 mmol, 5.00 equiv.), DCM (2.8 mL) and water (2.8 mL) were sequentially added. The resulting biphasic mixture was heated at 50 °C for 4 hours with vigorous stirring. The product was extracted with pentane (4 x 10 mL). The combined organic phases were washed with water (4 x 10 mL), dried over $MgSO_4$, and filtered. Concentration *in vacuo* afforded pure compound **3a** as a colourless oil in 52% yield (36 mg, 0.16 mmol).

8.2 Dibromo-pyrazabole + Cat. $[Et_3NH]NTf_2$

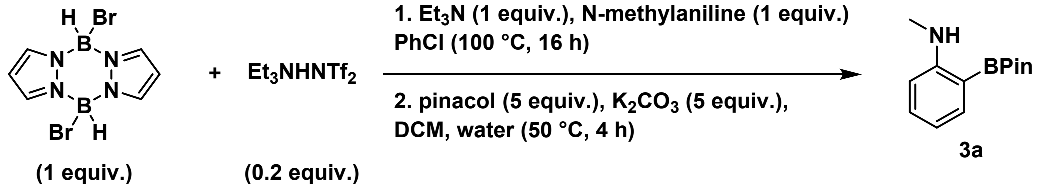

Dibromo-pyrazabole (95 mg, 0.30 mmol, 1.00 equiv.) and $[Et_3NH]NTf_2$ (23 mg, 0.06 mmol, 0.20 equiv.) were dissolved in PhCl (2 mL) at room temperature in a J. Young's ampoule. Et_3N (42 µL, 0.30 mmol, 1.00 equiv.) and N-methylaniline (33 µL, 0.30 mmol, 1.00 equiv.) were successively added to the solution. The reaction mixture was heated at 100 °C for 16 hours. The solution was cooled down to room temperature. Pinacol (177 mg, 1.50 mmol, 5.00 equiv.), K_2CO_3 (207 mg, 1.50 mmol, 5.00 equiv.), DCM (2.8 mL) and water (2.8 mL) were sequentially added. The resulting biphasic mixture was heated at 50 °C for 4 hours with vigorous stirring. The product was extracted with pentane (4 x 10 mL). The combined organic phases were washed with water (4 x 10 mL), dried over $MgSO_4$, and filtered. Volatiles were removed *in vacuo*. A mixture containing a small amount of borylated product **3a** (< 15%) and the di-N-methylaniline-pyrazabole **5** was obtained.

9 BDB with 7/cat $[Et_3NH]NTf_2$

9.1 General procedure 2

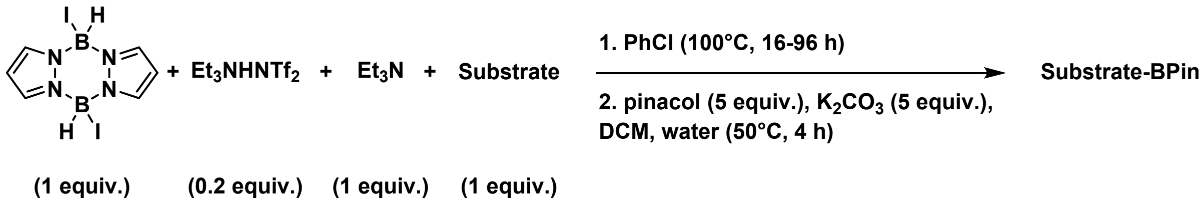

Diiodo-pyrazabole **7** (123 mg, 0.30 mmol, 1.00 equiv.), $[Et_3NH]NTf_2$ (23 mg, 0.06 mmol, 0.20 equiv.) were dissolved in PhCl (2 mL) in a J. Young's ampoule. Et_3N (42 µL, 0.30 mmol, 1.00 equiv.) and the substrate (0.30 mmol, 1.00 equiv.) were successively added to the solution. The reaction mixture was heated at 100 °C for 16-96 hours. The solution was cooled down to room temperature. Pinacol (177 mg, 1.50 mmol, 5.00 equiv.), K_2CO_3 (207 mg, 1.50 mmol, 5.00 equiv.), DCM (2.8 mL) and water (2.8 mL) were sequentially added. The resulting biphasic mixture was heated at 50 °C for 4 hours with vigorous stirring. The product was extracted with pentane (4 x 10 mL). The combined organic phases were washed with water (4 x 10 mL), dried over $MgSO_4$, and filtered. Concentration *in vacuo* afforded the desired product. *Further purification was enabled by reverse phase chromatography (C18, MeOH 100%), where required.* In some cases (yields were determined by addition of internal standard (Mesitylene).

The labelling of the different protons and carbons in the ^1H and ^{13}C-NMR assignment of the substituted N-alkyl-aniline was done following the convention bellow (Figure S69).

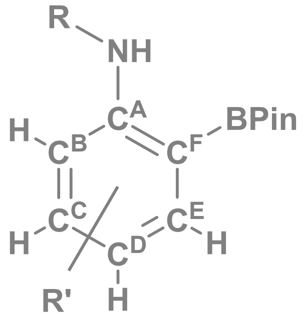

Figure S69: Scheme of the convention taken for the labelling of the protons and carbons of the substituted N-alkyl-anilines used for the substrate scope.

9.2 Substrate scope

9.2.1 *N-methyl-2-(BPin)aniline 3a*

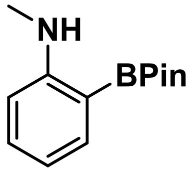

Compound **3a** was prepared following **General Procedure 2** with N-methylaniline (33 μL, 0.30 mmol, 1.00 equiv.). Reaction was heated at 100 °C for 16 hours. The product **3a** was isolated as a colourless oil (42 mg, 0.18 mmol, 60%) with no further purification necessary. Analytical data were in accordance with literature values.[S14]

^{1}H NMR (500 MHz, $CDCl_3$, 300 K) δ 7.64 (dd, $^3J_{HH}$ = 7.3 Hz, $^4J_{HH}$ = 1.8 Hz, C^EH, 1H), 7.33 (ddd, $^3J_{HH}$ = 8.6 Hz, $^3J_{HH}$ = 7.2 Hz, $^4J_{HH}$ = 1.8 Hz, C^CH, 1H), 6.63 (td, $^3J_{HH}$ = 7.2 Hz, $^4J_{HH}$ = 1.0 Hz, C^DH, 1H), 6.55 (d, $^3J_{HH}$ = 8.2 Hz, C^BH, 1H), 5.76 (m br, N*H*, 1H), 2.86 (d, $^3J_{HH}$ = 5.0 Hz, NCH_3, 3H), 1.34 (s, $OC(CH_3)_2$, 12H).

^{11}B NMR (160 MHz, $CDCl_3$, 300 K) δ 31.0 (s, *B*Pin, 1B).

^{13}C {^{1}H} NMR (126 MHz, $CDCl_3$, 300 K) δ 155.6 (s, C^A, 1C), 137.2 (s, C^EH, 1C), 133.3 (s, C^CH, 1C), 115.5 (s, C^DH, 1C), 110.9 (s, C^FB, 1C), 108.9 (s, C^BH, 1C), 83.6 (s, $OC(CH_3)_2$, 2C), 30.3 (s, NCH_3, 1C), 25.0 (s, $OC(CH_3)_2$, 4C).

9.2.2 *8-(BPin)-1,2,3,4-tetrahydroquinoline 3b*

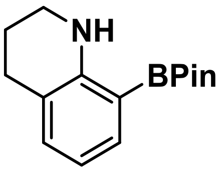

Compound **3b** was prepared following **General Procedure 2** with tetrahydroquinoline (38 μL, 0.30 mmol, 1.00 equiv.). Reaction was heated at 100 °C for 16 hours. The product **3b** was obtained as a colourless oil (60 mg, 0.23 mmol, 78%), with remaining trace of tetrahydroquinoline. Analytical data were in accordance with literature values.[S15]

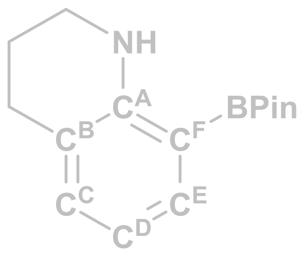

Figure S70: Scheme of the 8-(BPin)-1,2,3,4-tetrahydroquinoline **3b**.

^{1}H NMR (600 MHz, $CDCl_3$, 300 K) δ 7.45 (d, $^{3}J_{HH}$ = 7.4 Hz, $C^{E}H$, 1H), 7.00 (d, $^{3}J_{HH}$ = 7.2 Hz, $C^{C}H$, 1H), 6.51 (t, $^{3}J_{HH}$ = 7.3 Hz, $C^{D}H$, 1H), 5.85 (s br, N*H*, 1H), 3.39-3.34 (m, NCH_2, 2H), 2.76 (t, $^{3}J_{HH}$ = 6.6 Hz, $C^{B}CH_2$, 2H), 1.95-1.88 (m, NCH_2CH_2, 2H), 1.34 (s, $OC(CH_3)_2$, 12H).

^{11}B NMR (128 MHz, $CDCl_3$, 300 K) δ 31.1 (s, *B*Pin, 1B).

^{13}C {^{1}H} NMR (151 MHz, $CDCl_3$, 300 K) δ 151.5 (s, C^{A}, 1C), 135.1 (s, C^{E}, 1C), 133.0 (s, C^{B}, 1C), 120.3 (s, C^{C}, 1C), 115.0 (s, C^{D}, 1C), 83.5 (s, $OC(CH_3)_2$, 2C), 41.8 (s, NCH_2, 1C), 27.8 (s, $C^{B}CH_2$, 1C), 25.0 (s, $OC(CH_3)_2$, 4C), 21.73 (s, NCH_2CH_2).*

*C–B was not observed by ^{13}C NMR.

9.2.3 N-isopropyl-2-(BPin)aniline 3c

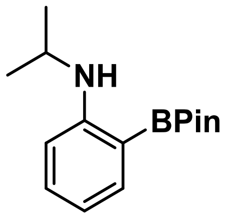

Compound **3c** was prepared following **General Procedure 2** with N-isopropylaniline (44 µL, 0.30 mmol, 1.00 equiv.). Reaction was heated at 100 °C for 16 hours. The product **3c** was isolated as a colourless oil (41 mg, 0.18 mmol, 52%) with no further purification necessary.

^{1}H NMR (500 MHz, $CDCl_3$, 300 K) δ 7.61 (dd, $^{3}J_{HH}$ = 7.4 Hz, $^{4}J_{HH}$ = 1.8 Hz, $C^{E}H$, 1H), 7.29-7.25 (m, $C^{C}H$, 1H), 6.60-6.54 (m, $C^{B}H$ and $C^{D}H$, 2H), 5.74 (d, $^{3}J_{HH}$ = 5.6 Hz, N*H*, 1H), 3.67-3.57 (m, $NCH(CH_3)_3$, 1H), 1.33 (s, $OC(CH_3)_2$, 12H) , 1.23 (d, $^{3}J_{HH}$ = 6.3 Hz, $NCH(CH_3)_3$, 6H).

^{11}B NMR (160 MHz, $CDCl_3$, 300 K) δ 30.9 (s, *B*Pin, 1B).

^{13}C {^{1}H} NMR (126 MHz, $CDCl_3$, 300 K) δ 154.1 (s, C^{A}N, 1C), 137.4 (s, C^{E}H, 1C), 133.1 (s, C^{C}H, 1C), 115.2 (s, C^{D}H, 1C), 111.1 (s br, C^{F}BPin, 1C), 110.2 (s, C^{B}H, 1C), 83.5 (s, $OC(CH_3)_2$, 2C), 43.9 (s, $NCH(CH_3)_2$, 1C), 25.1 (s, $OC(CH_3)_2$, 4C), 23.1 (s, $NCH(CH_3)_2$, 2C).

Mass Spectrum: HRMS (ESI^{+}) m/z calculated for $C_{15}H_{24}B_1N_1O_2Na_1$: 284.1792; Found: 284.1794.

9.2.4 9-(BPin)-2,3,4,5-Tetrahydro-1-benzo[b]azepine 3d

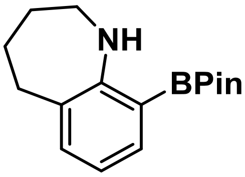

Compound **3d** was prepared following **General Procedure 2** with 2,4,3,5-Tetrahydro-1-benzo[b]azepine (44 mg, 0.30 mmol, 1.00 equiv.). Reaction was heated at 100 °C for 20 hours. After organic work up, extended overnight drying under vacuum allowed purification of the product. The product **3d** was obtained as a white solid (44 mg, 0.16 mmol, 54%).

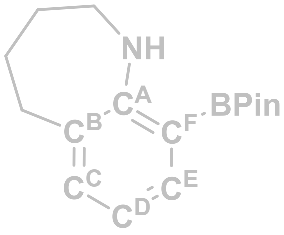

Figure S71: Scheme of the 9-(BPin)-2,3,4,5-Tetrahydro-1-benzo[b]azepine **3d**.

^{1}H NMR (500 MHz, $CDCl_3$, 300 K) δ 7.55 (dd, $^3J_{HH}$ = 7.4 Hz, $^4J_{HH}$ = 1.7 Hz, C^EH, 1H), 7.18 (m, C^CH, 1H), 6.77 (t, $^3J_{HH}$ = 7.3 Hz, C^DH, 1H), 5.92 (s, N*H*, 1H), 3.07 (m, NCH_2, 2H), 2.76 (m, C^BCH_2, 2H), 1.79 (m, NCH_2CH_2, 2H), 1.66 (m, $C^BCH_2CH_2$, 2H), 1.33 (s, $OC(CH_3)_2$, 12H).

^{11}B NMR (160 MHz, $CDCl_3$, 300 K) δ 31.0 (s, *B*Pin, 1B).

^{13}C {^{1}H} NMR (126 MHz, $CDCl_3$, 300 K) δ 158.0 (s, C^A, 1C), 134.7 (s, C^E, 1C), 134.6 (s, C^C, 1C), 133.3 (s, C^B, 1C), 119.5 (s, C^D, 1C), 116.3 (s br, C^F, 1C),* 83.7 (s, $OC(CH_3)_2$, 2C), 48.3 (s, NCH_2, 1C), 36.3 (s, C^BCH_2, 1C), 31.7 (s, NCH_2CH_2, 1C), 26.9 (s, $C^BCH_2CH_2$, 1C), 25.0 (s, $OC(CH_3)_2$, 4C).

*C–B was observed as a small broad singlet in the ^{13}C NMR and by ^{1}H-^{13}C HMBC NMR experiment.

Mass Spectrum: HRMS (EI$^+$) m/z calculated for $C_{16}H_{24}B_1N_1O_2$: 273.18946; Found: 273.18913.

9.2.5 7-(BPin)indoline 3e

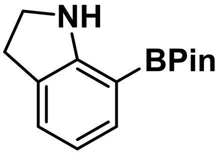

Compound **3e** was prepared following **General Procedure 2** with indoline (34 µL, 0.30 mmol, 1.00 equiv.). Reaction was heated at 100 °C for 16 hours. Extended drying under vacuum (10^{-1} - 10^{-2} mbar) afforded **3e** as a white crystalline solid (42 mg, 0.17 mmol, 58%). Traces of pyrazabole derivative impurity (< 5%) were observed by NMR spectroscopy and can be removed by reverse phase chromatographic column if higher purity is desired, however, partial protodeborylation will occur during chromatography and lower the yield during the process.

Analytical data were in accordance with literature values.[S1]

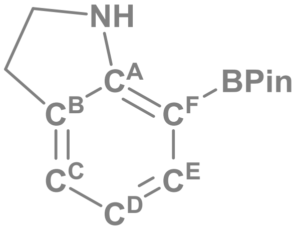

Figure S72: Scheme of the 7-(BPin)indoline **3e**.

^{1}H NMR (600 MHz, $CDCl_3$, 300 K) δ 7.38 (d, $^{3}J_{HH}$ = 7.5 Hz, $C^{E}H$, 1H), 7.15 (d, $^{3}J_{HH}$ = 7.1 Hz, $C^{C}H$, 1H), 6.61 (t, $^{3}J_{HH}$ = 7.3 Hz, $C^{D}H$, 1H), 5.02 (s br, N*H*, 1H), 3.60 (t, $^{3}J_{HH}$ = 8.4 Hz, NCH_2, 2H), 3.00 (t, $^{3}J_{HH}$ = 8.4 Hz, NCH_2CH_2, 2H), 1.33 (s, $OC(CH_3)_2$, 12H).

^{11}B NMR (128 MHz, $CDCl_3$, 300 K) δ 30.9 (s, *B*Pin, 1B).

^{13}C {^{1}H} NMR (151 MHz, $CDCl_3$, 300 K) δ 158.7 (s, C^A, 1C), 133.7 (s, C^E, 1C), 128.5 (s, C^B, 1C), 127.6 (s, C^C, 1C), 117.0 (s, C^D, 1C), 106.2 (s, C^F, 1C),* 83.5 (s, $OC(CH_3)_2$, 2C), 47.1 (s, NCH_2, 1C), 29.3 (s, NCH_2CH_2, 1C), 25.1 (s, $OC(CH_3)_2$, 4C).

*C–B was observed by ^{1}H-^{13}C HMBC NMR experiment.

9.2.6 3-N-dimethyl-2-(BPin)aniline 3f

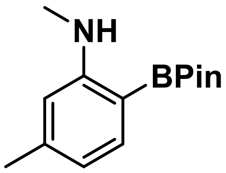

Compound **3f** was prepared following **General Procedure 2** with 3-N-dimethylaniline (38 μL, 0.30 mmol, 1.00 equiv.). Reaction was heated at 100 °C for 20 hours. The product **3f** was purified by reverse phase chromatography (C18, MeOH 100%) and isolated as a colourless oil (31 mg, 0.13 mmol, 42%).

^{1}H NMR (400 MHz, $CDCl_3$, 300 K) δ 7.53 (d, $^{3}J_{HH}$ = 7.4 Hz, $C^{E}H$, 1H), 6.47 (dq, $^{3}J_{HH}$ = 7.5 Hz, $^{4}J_{HH}$ = 0.8 Hz, $C^{D}H$, 1H), 6.37 (s br, $C^{B}H$, 1H), 5.72 (s br, N*H*, 1H), 2.85 (s, NCH_3, 3H), 2.31 (s, $C^{C}CH_3$, 3H), 1.32 (s, $OC(CH_3)_2$, 12H).

^{11}B NMR (128 MHz, $CDCl_3$, 300 K) δ 31.0 (s, *B*Pin, 1B).

^{13}C {^{1}H} NMR (101 MHz, $CDCl_3$, 300 K) δ 155.7 (s, C^{A}N, 1C), 143.7 (s, $C^{C}CH_3$, 1C), 137.3 (s, C^{E}H, 1C), 116.7 (s, C^{D}H, 1C), 109.7 (s, C^{B}H, 1C), 107.9 (s br, C^{F}BPin, 1C),* 83.5 (s, $OC(CH_3)_2$, 2C), 30.3 (s, NCH_3, 1C), 25.0 (s, $OC(CH_3)_2$, 4C), 22.3 (s, $C^{C}CH_3$, 1C).

*C–BPin assigned by ^{1}H-^{13}C HMBC NMR experiment.

Mass Spectrum: HRMS (EI^{+}) m/z calculated for $C_{14}H_{22}B_{1}N_{1}O_{2}$: 247.17381; Found: 247.17347.

9.2.7 4-N-dimethyl-2-(BPin)aniline 3g

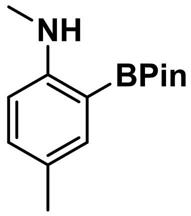

Compound **3g** was prepared following **General Procedure 2** with 4-N-dimethylaniline (38 μL, 0.30 mmol, 1.00 equiv.). Reaction was heated at 100 °C for 20 hours. The product **3g** was purified by reverse phase chromatography (C18, MeOH 100%) and isolated as a white crystalline solid (44 mg, 0.18 mmol, 59%).

^{1}**H NMR** (500 MHz, $CDCl_3$, 300 K) δ 7.45 (d, $^{4}J_{HH}$ = 2.3 Hz, C^{E}*H*, 1H), 7.15 (ddd, $^{3}J_{HH}$ = 8.3 Hz, $^{4}J_{HH}$ = 2.4 Hz, $^{4}J_{HH}$ = 0.8 Hz, C^{C}*H*, 1H), 6.49 (d, $^{3}J_{HH}$ = 8.3 Hz, C^{B}*H*, 1H), 5.60 (s, N*H*, 1H), 2.84 (s, NC*H*$_3$, 3H), 2.23 (s, C^{D}C*H*$_3$, 3H), 1.34 (s, OC(C*H*$_3$)$_2$, 12H).

^{11}**B NMR** (160 MHz, $CDCl_3$, 300 K) δ 31.1 (s, *B*Pin, 1B).

^{13}**C {^{1}H} NMR** (126 MHz, $CDCl_3$, 300 K) δ 153.6 (s, *C^{A}*N, 1C), 137.3 (s, *C^{E}*H, 1C), 133.4 (s, *C^{C}*H, 1C), 124.4 (s, *C^{D}*CH$_3$, 1C), 111.0 (s br, *C^{F}*BPin, 1C), 109.2 (s, *C^{B}*H, 1C), 83.6 (s, O*C*(CH$_3$)$_2$, 2C), 30.5 (s, N*C*H$_3$, 1C), 25.0 (s, OC(*C*H$_3$)$_2$, 4C), 20.3 (s, C^{D}*C*H$_3$, 1C).

Mass Spectrum: HRMS (EI^{+}) m/z calculated for $C_{14}H_{22}B_1N_1O_2$: 247.17381; Found: 247.17381.

9.2.8 4-fluoro-N-methyl-2-(BPin)aniline 3h

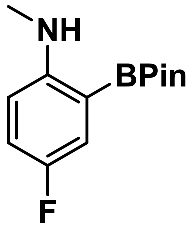

Compound **3h** was prepared following **General Procedure 2** with 4-fluoro-N-methylaniline (36 µL, 0.30 mmol, 1.00 equiv.). Reaction was heated at 100 °C for 96 hours. After organic work up, extended drying under vacuum for 1 hour allowed purification of the product. The product **3h** was obtained as a colourless oil (37 mg, 0.15 mmol, 50%).

^{1}**H NMR** (500 MHz, $CDCl_3$, 300 K) δ 7.32 (dd, $^{3}J_{HF}$ = 9.1 Hz, $^{4}J_{HH}$ = 3.2 Hz, C^{E}*H*, 1H), 7.02 (ddd, $^{3}J_{HF}$ = 9.0 Hz, $^{3}J_{HH}$ = 8.2 Hz, $^{4}J_{HH}$ = 3.2 Hz, C^{C}*H*, 1H), 6.46 (dd, $^{3}J_{HH}$ = 9.0 Hz, $^{4}J_{HF}$ = 4.1 Hz, C^{B}*H*, 1H), 5.58 (s br, N*H*, 1H), 2.83 (d, $^{3}J_{HH}$ = 2.8 Hz, NC*H*$_3$, 3H), 1.33 (s, OC(C*H*$_3$)$_2$, 12H).

^{11}**B NMR** (160 MHz, $CDCl_3$, 300 K) δ 30.5 (s, *B*Pin, 1B).

^{13}**C {^{1}H} NMR** (126 MHz, $CDCl_3$, 300 K) δ 154.6 (d, $^{1}J_{CF}$ = 233.4 Hz, *C^{D}*F, 1C), 152.1 (s, *C^{A}*N, 1C), 122.4 (d, $^{2}J_{CF}$ = 20.1 Hz, *C^{E}*H, 1C), 119.8 (d, $^{2}J_{CF}$ = 22.4 Hz, *C^{C}*H, 1C), 111.6 (s br, *C^{F}*BPin, 1C), 109.9 (d, $^{3}J_{H}$ = 6.7 Hz, *C^{B}*H, 1C), 84.0 (s, O*C*(CH$_3$)$_2$, 2C), 30.8 (s, N*C*H$_3$, 1C), 25.0 (s, OC(*C*H$_3$)$_2$, 4C).

^{19}**F NMR** (471 MHz, $CDCl_3$, 300 K) δ –131.5 (td, $^{3}J_{FH}$ = 9.1 Hz, $^{4}J_{FH}$ = 4.5 Hz), C^{D}*F*, 1F).

Mass Spectrum: HRMS (EI^+) m/z calculated for $C_{13}H_{19}B_1N_1O_2F_1$: 251.14874; Found: 251.14887.

9.2.9 3-bromo-N-methyl-2-(BPin)aniline 3i

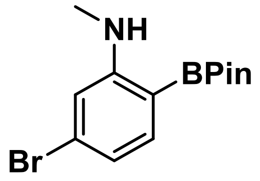

Compound **3i** was prepared following **General Procedure 2** with 3-bromo-N-methylaniline (39 µL, 0.30 mmol, 1.00 equiv.). Reaction was heated at 100 °C for 72 hours. The product **3i** was purified by reverse phase chromatography (C18, MeOH 100%) and isolated as a colourless oil (22 mg, 0.07 mmol, 24%).*

*Multiple attempts were tried to improve the yield, however these were unsuccessful. Longer reaction time (96 hours, 100 °C) or higher temperature (72 hours, 120 °C) did not result in any yield improvement.

^{1}H NMR (600 MHz, $CDCl_3$, 300 K) δ 7.45 (d, $^3J_{HH}$ = 7.8 Hz, C^EH, 1H), 6.73 (dd, $^3J_{HH}$ = 7.9 Hz, $^4J_{HH}$ = 1.8 Hz, C^DH, 1H), 6.65 (d, $^3J_{HH}$ = 1.7 Hz, C^BH, 1H), 5.87-5.79 (m br, N*H*, 1H), 2.83 (d, $^3J_{HH}$ = 5.0 Hz, NCH_3, 3H), 1.32 (s, $OC(CH_3)_2$, 12H).

^{11}B NMR (160 MHz, $CDCl_3$, 300 K) δ 30.7 (s, *B*Pin, 1B).

^{13}C {^{1}H} NMR (126 MHz, $CDCl_3$, 300 K) δ 156.4 (s, C^AN, 1C), 138.4 (s, C^EH, 1C), 128.4 (s, C^CBr, 1C), 118.4 (s, C^DH, 1C), 111.8 (s, C^BH, 1C), 109.7 (s, C^FBPin, 1C),* 83.9 (s, $OC(CH_3)_2$, 2C), 30.2 (s, NCH_3, 1C), 25.0 (s, $OC(CH_3)_2$, 4C).

*C–BPin was observed by ^{1}H-^{13}C NMR HMBC experiment.

Mass Spectrum: HRMS (EI^+) m/z calculated for $C_{13}H_{19}B_1N_1O_2Br_1$: 312.0765; Found: 312.0770.

9.2.10 4-piperidine-N-methyl-2-(BPin)aniline 3j

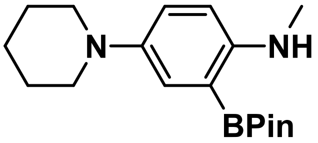

Diiodo-pyrazabole **7** (123 mg, 0.30 mmol, 1.00 equiv.), [Et_3NH]NTf_2 (23 mg, 0.06 mmol, 0.20 equiv.) were dissolved in PhCl (2 mL) in a J. Young's ampoule. Et_3N (42 µL, 0.30 mmol, 1.00 equiv.) and a solution of 4-piperidine-N-methylaniline (57 mg, 0.30 mmol, 1.00 equiv.) in PhCl (1 mL) were successively added to the solution. The reaction mixture was heated at 100 °C for 72 hours. The solution was cooled down to room temperature. Pinacol (177 mg, 1.50 mmol, 5.00 equiv.), K_2CO_3 (207 mg, 1.50 mmol, 5.00 equiv.), DCM (2.8 mL) and water (2.8 mL) were sequentially added. The resulting biphasic mixture was heated at 50 °C for 4 hours with vigorous stirring. The product was extracted with pentane (4 x 10 mL). The combined organic phases were washed with water (4 x 10 mL), dried over $MgSO_4$ and filtered. Drying *in vacuo* afforded the product **3j** in 62% yield (60 mg, 0.19 mmol) with a small amount of remaining 4-piperidine-N-methylaniline (13 mg). The product **3j** could be isolated from the starting material by reverse phase chromatography (C18, MeOH 100%) as a green oil (31 mg, 0.10 mmol, 33%).*

*Product **3j** and the 4-piperidine-N-methylaniline have almost identical Rf values under the solvent conditions explored. During purification, another fraction was collected which contained the product **3j** and 4-piperidine-N-methylaniline leading to important yield loss of isolated product. However, the sensitivity to protodeborylation of **3j** precluded us to investigate different mobile or stationary phases.

^{1}H NMR (600 MHz, $CDCl_3$, 300 K) δ 7.33 (d, $^4J_{HH}$ = 3.0 Hz, C^EH, 1H), 7.06 (dd, $^3J_{HH}$ = 8.8 Hz, $^4J_{HH}$ = 3.0 Hz, C^CH, 1H), 6.52 (d, $^3J_{HH}$ = 8.8 Hz, C^BH, 1H), 5.48 (s, N*H*, 1H), 2.99-2.94 (m, NCH_2, 4H), 2.83 (s, NCH_3, 3H), 1.75-1.68 (m, NCH_2CH_2, 4H), 1.54-1.49 (m, $NCH_2CH_2CH_2$, 2H), 1.32 (s, $OC(CH_3)_2$, 12H).

^{11}B NMR (160 MHz, $CDCl_3$, 300 K) δ 30.9 (s, *B*Pin, 1B).

^{13}C NMR (126 MHz, $CDCl_3$, 300 K) δ 150.8 (s, C^A, 1C), 143.2 (s, C^D, 1C), 127.2 (s, C^E, 1C), 124.6 (s, C^C, 1C), 111.4 (s, C^F, 1C),* 109.9 (s, C^B, 1C), 83.6 (s, $OC(CH_3)_2$, 2C), 53.4 (s, NCH_2, 2C), 30.8 (s, NCH_3, 1C), 26.5 (s, NCH_2CH_2, 2C), 25.0 (s, $OC(CH_3)_2$, 4C), 24.4 (s, $NCH_2CH_2CH_2$, 1C).

*C–B was observed by ^{1}H-^{13}C HMBC NMR experiment.

Mass Spectrum: HRMS (EI^+) m/z calculated for $C_{18}H_{29}B_1N_2O_2$: 316.23166; Found: 316.23252.

9.3 **Scaled-up preparation of N-methyl-2-(BPin)aniline 3a**

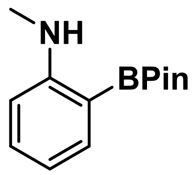

Iodine (0.76 g, 3.00 mmol, 1.00 equiv.) was added to a solution of pyrazabole (0.48 g, 3.00 mmol, 1.00 equiv.) in PhCl (15 mL) at room temperature in a J. Young's ampoule. After the initial bubbling, the ampoule was sealed and stirred for 2 hours at room temperature. Et_3NHNTf_2 (0.23 g, 0.60 mmol, 0.20 equiv.) in PhCl (5 mL), Et_3N (0.30 g, 3.00 mmol, 1.00 equiv.) and N-methylaniline (0.32 g, 3.00 mmol, 1.00 equiv.) were successively added. The flask was sealed and heated at 100 °C for 16 hours. The solution was cooled down to room temperature. Pinacol (1.77 g, 15.00 mmol, 5.00 equiv.), K_2CO_3 (2.07 g, 15.0 mmol, 5.00 equiv.), DCM (25 mL) and water (25 mL) were sequentially added. The resulting biphasic mixture was heated at 50 °C for 4 hours with vigorous stirring. The product was extracted with pentane (4 x 25 mL). The combined organic phases were washed with water (4 x 25 mL), dried over $MgSO_4$, filtered and concentration *in vacuo*. The crude **3a** was purified by reverse phase chromatography (C18, MeOH 100%), followed by extended vacuum drying affording pure **3a** as a colourless oil in 62% yield (0.43 g, 1.85 mmol).

9.4 **Glovebox free preparation of N-methyl-2-(BPin)aniline 3a**

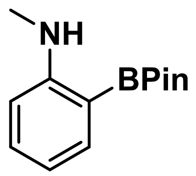

This preparation did not involve the use of a glovebox. All the chemicals (except the solvent PhCl which was dried and stored under inert atmosphere) used during this procedure were stored and handled on the bench.

Iodine (76 mg, 0.30 mmol, 1.00 equiv.) was added to a solution of pyrazabole (47 mg, 0.30 mmol, 1.00 equiv.) in PhCl (2 mL) at room temperature in a J. Young's ampoule. After the initial bubbling, the ampoule was sealed and stirred for 2 hours at room temperature. Et_3NHNTf_2 (23 mg, 0.06 mmol, 0.20 equiv.) in PhCl (1 mL), Et_3N (42 µL g, 0.30 mmol, 1.00 equiv.) and N-methylaniline (33 µL, 0.30 mmol, 1.00 equiv.) were successively added. The flask was sealed and heated at 100 °C for 16 hours. The solution was cooled down to room temperature. Pinacol (177 mg, 1.50 mmol, 5.00 equiv.), K_2CO_3 (207 mg, 1.50 mmol, 5.00 equiv.), DCM (2.8 mL) and water (2.8 mL) were sequentially added. The resulting biphasic

mixture was heated at 50 °C for 4 hours with vigorous stirring. The product was extracted with pentane (4 x 10 mL). The combined organic phases were washed with water (4 x 10 mL), dried over $MgSO_4$ and filtered. Concentration *in vacuo* afforded pure **3a** as a colourless oil in 45% yield (31 mg, 0.13 mmol).

9.5 NMR Spectra

9.5.1 NMR spectra of N-isopropyl-2-(BPin)aniline 3c

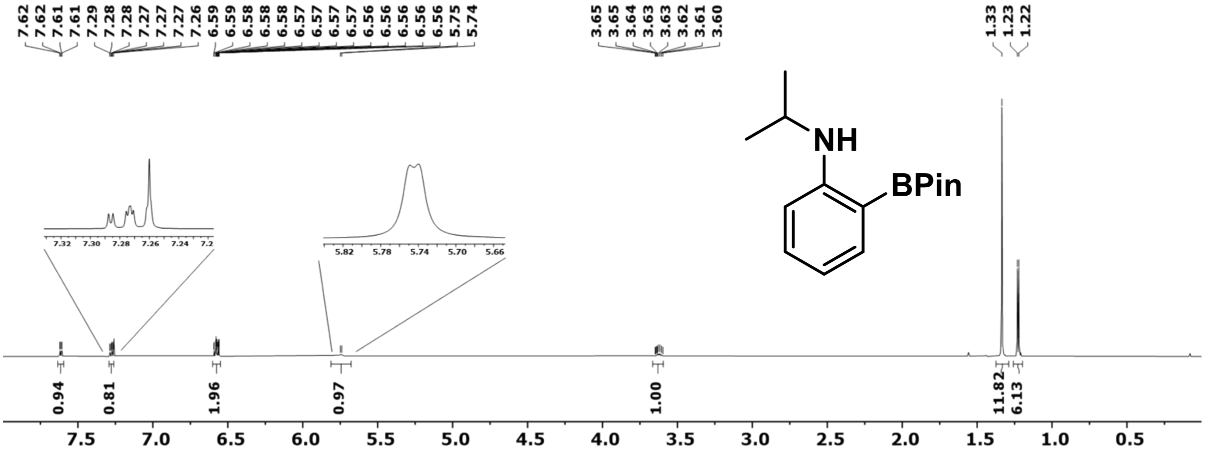

Figure S73: ^{1}H NMR spectrum (500 MHz, $CDCl_3$, 300 K) of the N-isopropyl-2-(BPin)aniline **3c**.

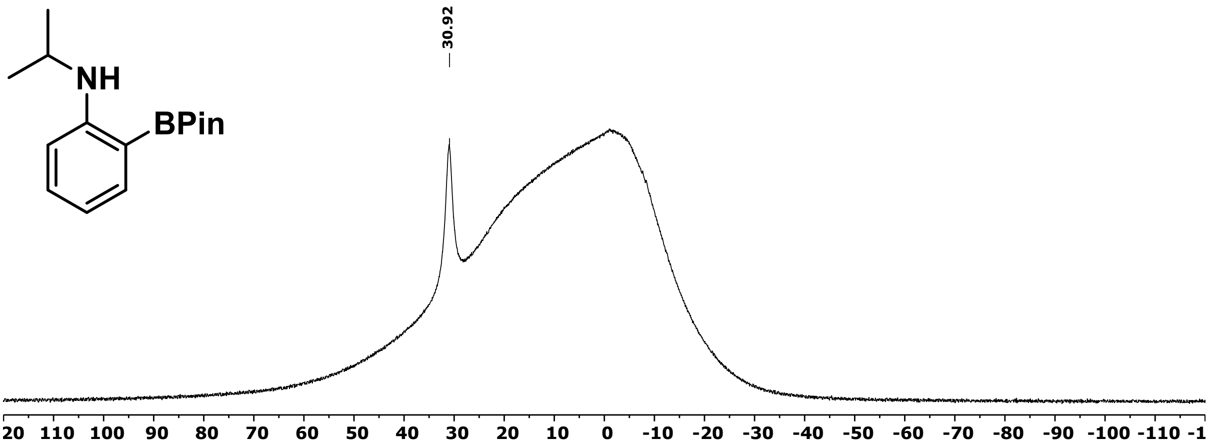

Figure S74: ^{11}B NMR spectrum (160 MHz, $CDCl_3$, 300 K) of the N-isopropyl-2-(BPin)aniline **3c**.

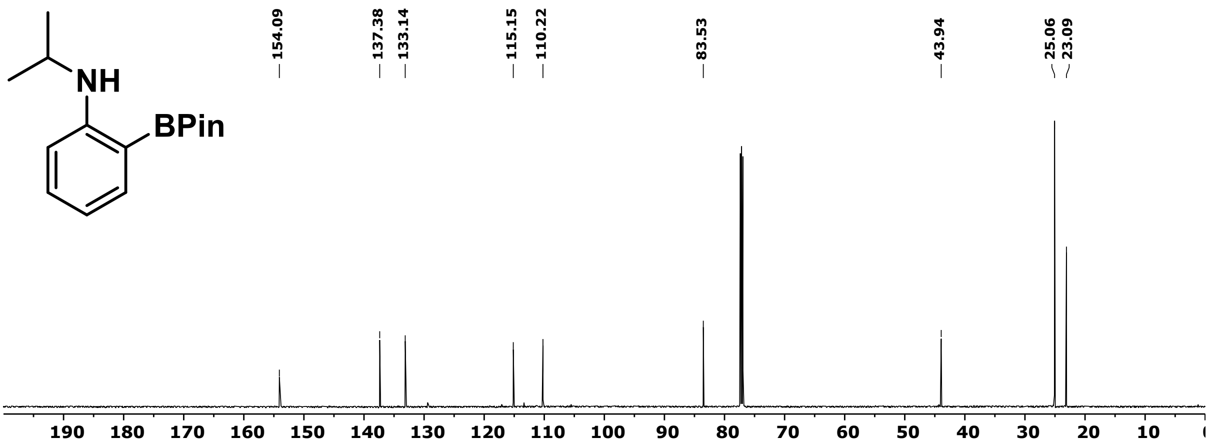

Figure S75: ^{13}C {^{1}H} NMR spectrum (126 MHz, $CDCl_3$, 300 K) of the N-isopropyl-2-(BPin)aniline **3c**.

9.5.2 NMR spectra of 9-(BPin)-2,3,4,5-Tetrahydro-1-benzo[b]azepine 3d

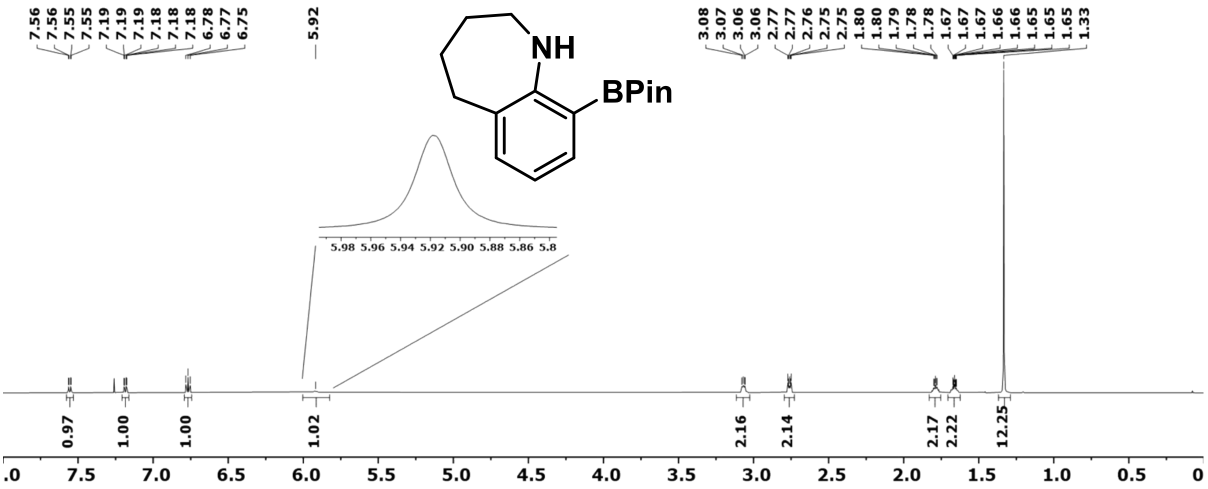

Figure S76: ^{1}H NMR spectrum (500 MHz, $CDCl_3$, 300 K) of the 9-(BPin)-2,3,4,5-Tetrahydro-1-benzo[b]azepine **3d**.

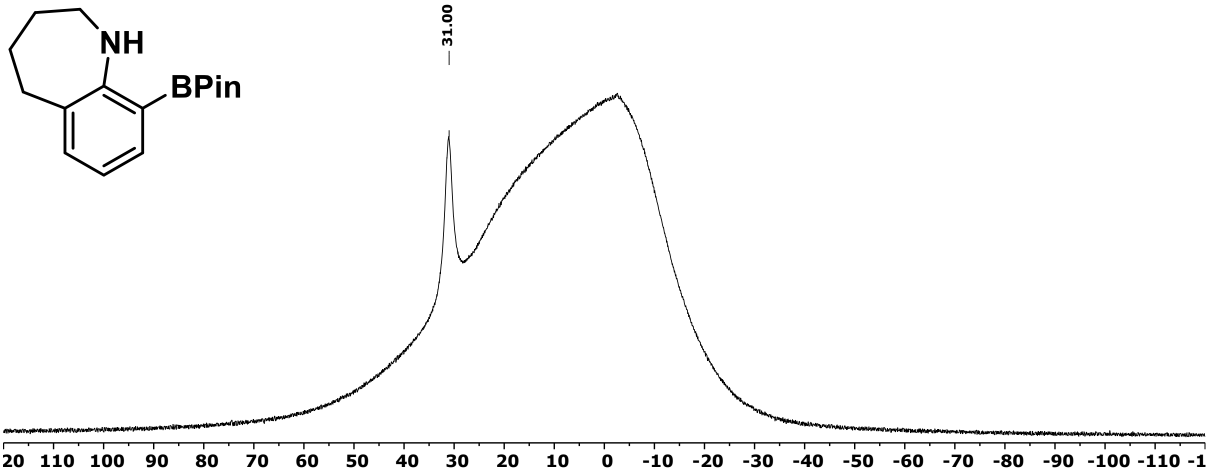

Figure S77: ^{11}B NMR spectrum (160 MHz, $CDCl_3$, 300 K) of the 9-(BPin)-2,3,4,5-Tetrahydro-1-benzo[b]azepine **3d**.

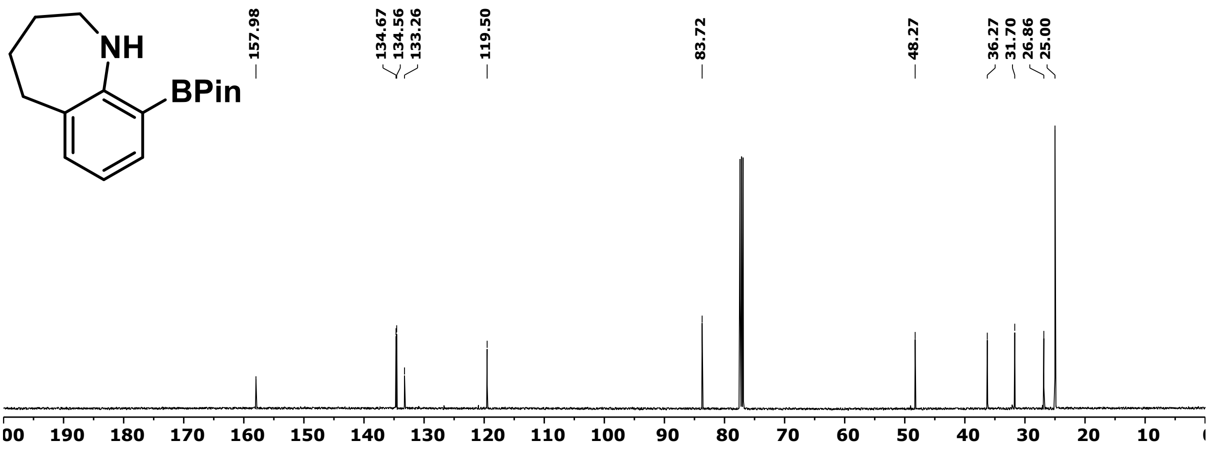

Figure S78: ^{13}C {^{1}H} NMR spectrum (126 MHz, $CDCl_3$, 300 K) of the 9-(BPin)-2,3,4,5-Tetrahydro-1-benzo[b]azepine **3d**.

9.5.3 NMR spectra of 3-N-dimethyl-2-(BPin)aniline 3f

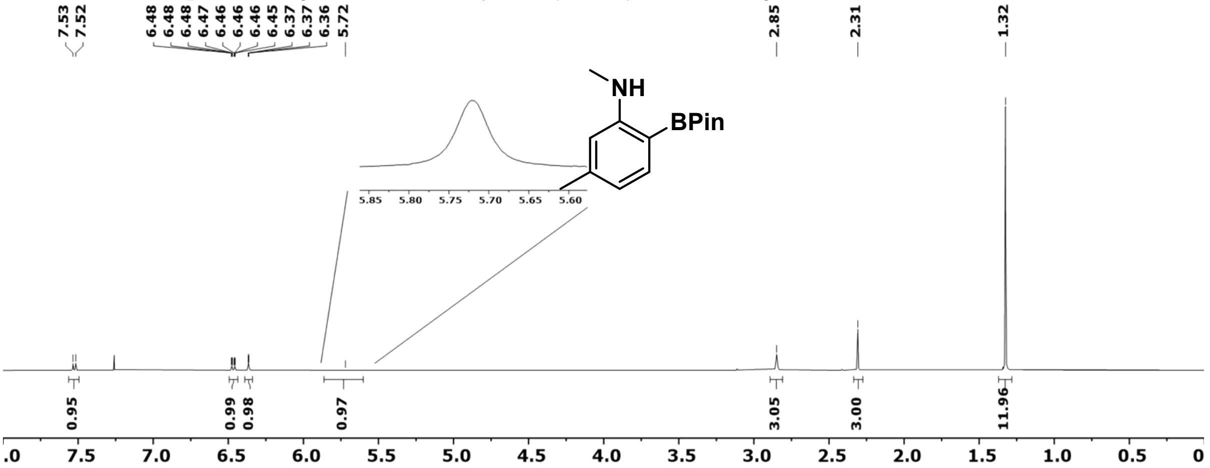

Figure S79: ^{1}H NMR spectrum (400 MHz, $CDCl_3$, 300 K) of the 3-N-dimethyl-2-(BPin)aniline **3f**.

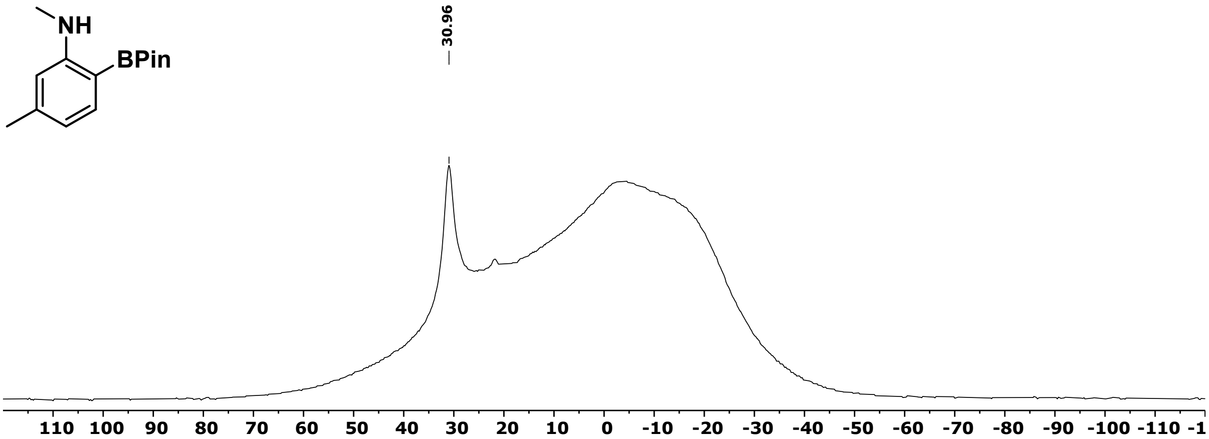

Figure S80: ^{11}B NMR spectrum (128 MHz, $CDCl_3$, 300 K) of the 3-N-dimethyl-2-(BPin)aniline **3f**.

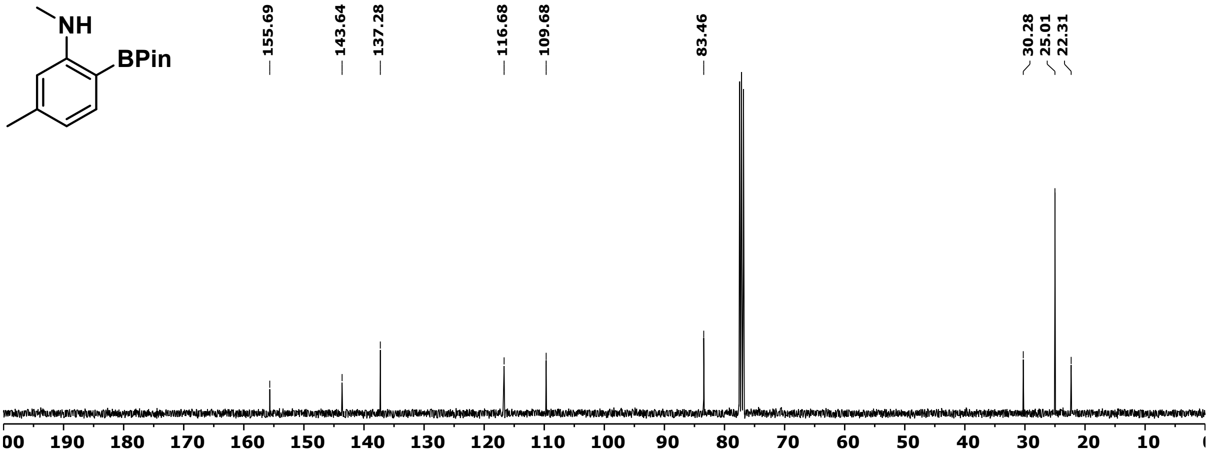

Figure S81: ^{13}C {^{1}H} NMR spectrum (101 MHz, $CDCl_3$, 300 K) of the 3-N-dimethyl-2-(BPin)aniline **3f**.

9.5.4 NMR spectra of 4-N-dimethyl-2-(BPin)aniline 3g

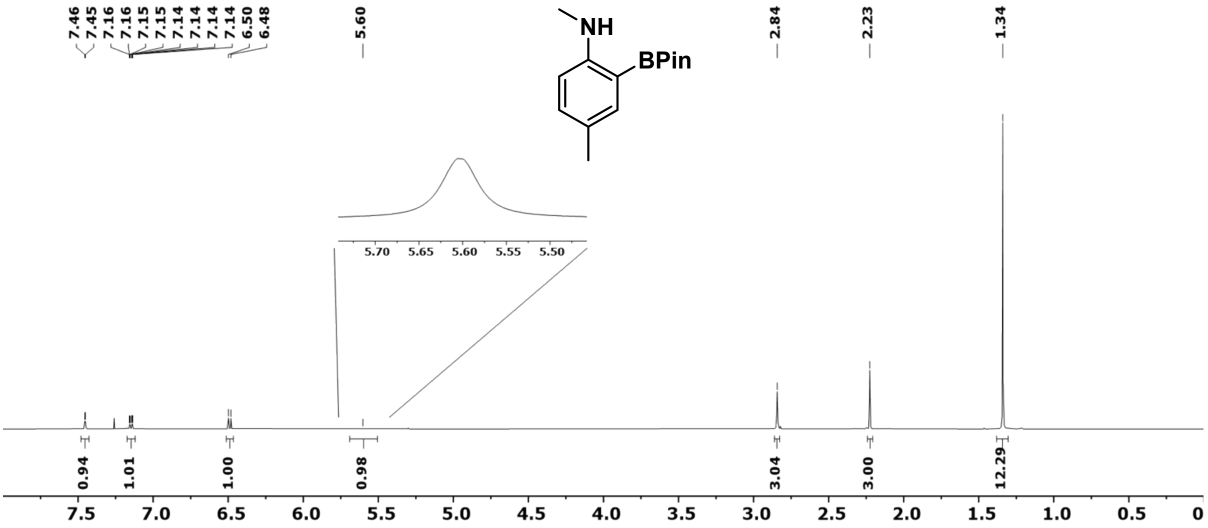

Figure S82: ^{1}H NMR spectrum (500 MHz, $CDCl_3$, 300 K) of the 4-N-dimethyl-2-(BPin)aniline **3g**.

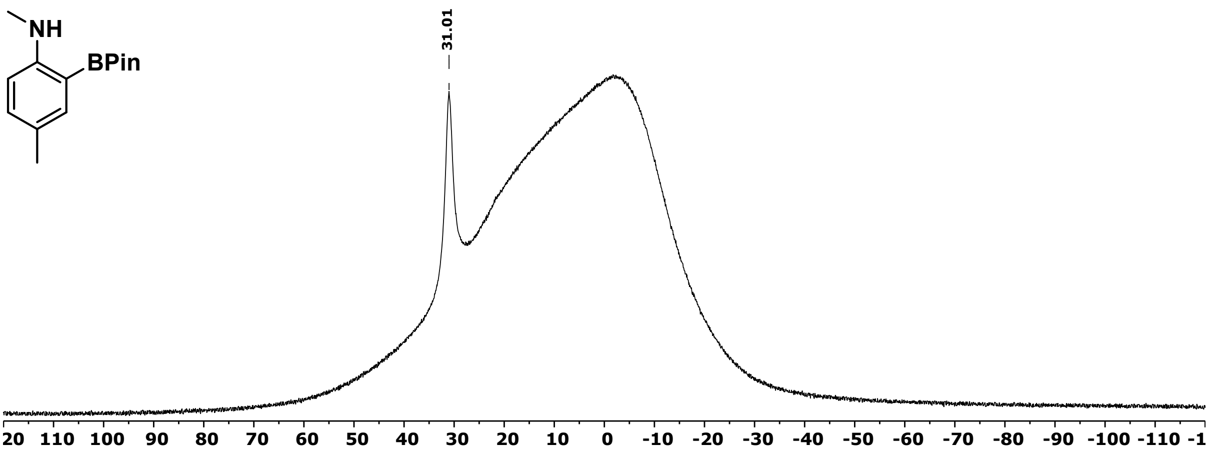

Figure S83: ^{11}B NMR spectrum (160 MHz, $CDCl_3$, 300 K) of the 4-N-dimethyl-2-(BPin)aniline **3g**.

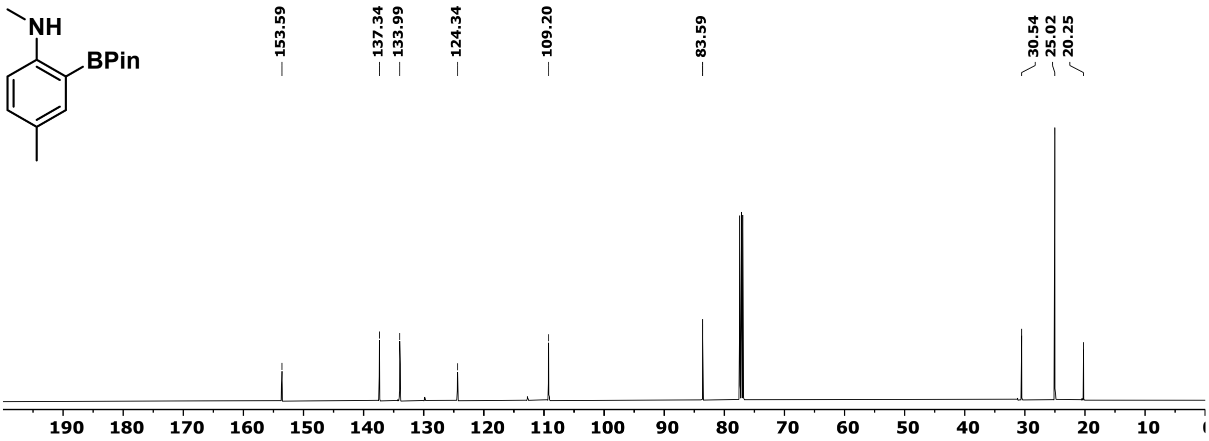

Figure S84: ^{13}C {^{1}H} NMR spectrum (126 MHz, $CDCl_3$, 300 K) of the 4-N-dimethyl-2-(BPin)aniline **3g**.

9.5.5 NMR spectra of 4-fluoro-N-methyl-2-(BPin)aniline 3h

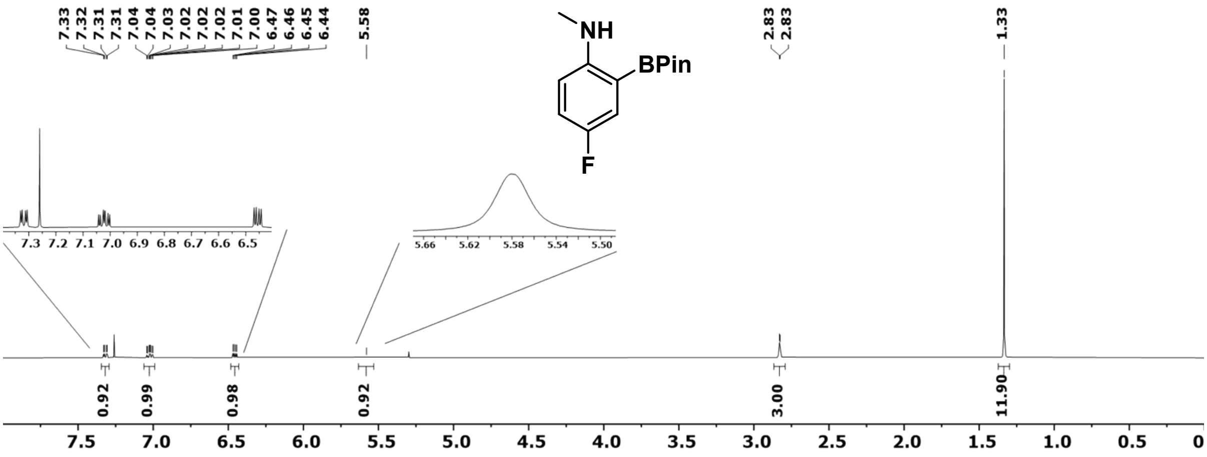

Figure S85: ^{1}H NMR spectrum (500 MHz, $CDCl_3$, 300 K) of the 4-fluoro-N-methyl-2-(BPin)aniline **3h**.

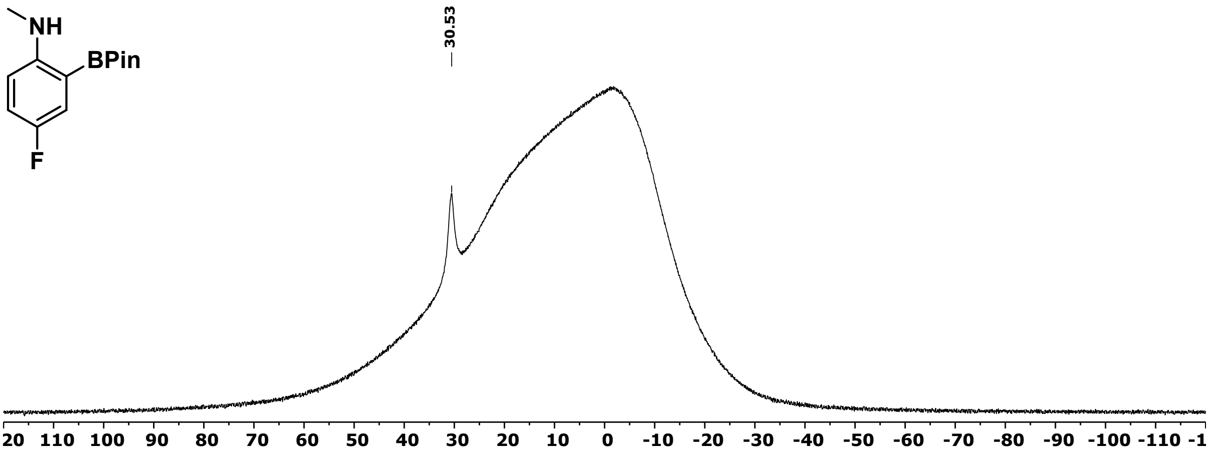

Figure S86: ^{11}B NMR spectrum (160 MHz, $CDCl_3$, 300 K) of the 4-fluoro-N-methyl-2-(BPin)aniline **3h**.

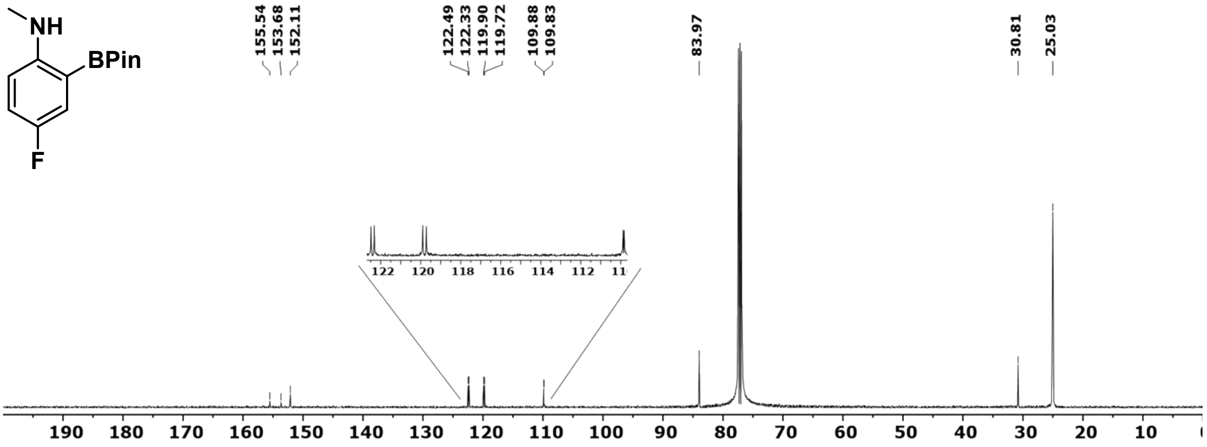

Figure S87: ^{13}C {^{1}H} NMR spectrum (126 MHz, $CDCl_3$, 300 K) of the 4-fluoro-N-methyl-2-(BPin)aniline **3h**.

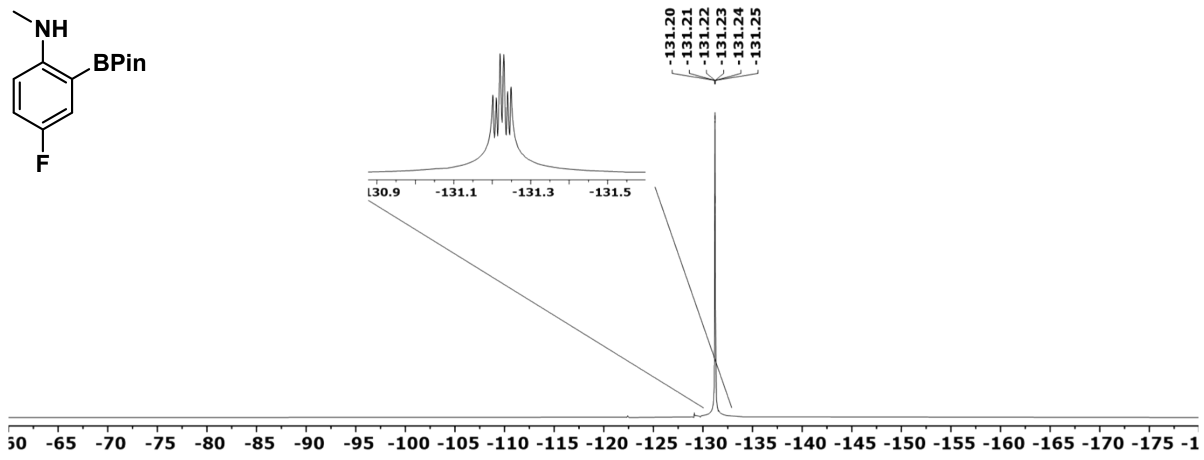

Figure S88: ^{19}F NMR spectrum (471 MHz, $CDCl_3$, 300 K) of the 4-fluoro-N-methyl-2-(BPin)aniline **3h**.

9.5.6 NMR spectra of 3-bromo-N-methyl-2-(BPin)aniline 3i

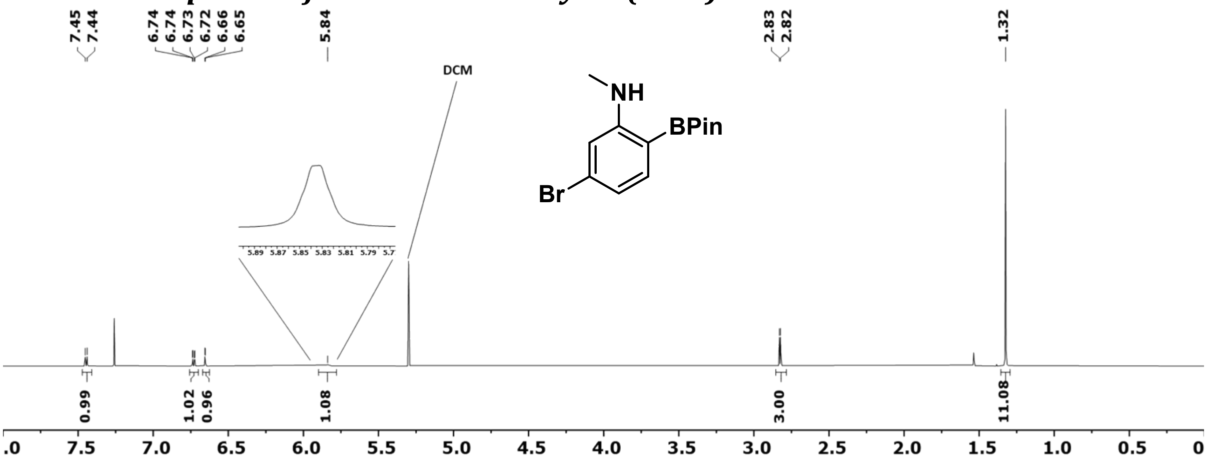

Figure S89: ^{1}H NMR spectrum (600 MHz, $CDCl_3$, 300 K) of the 3-bromo-N-methyl-2-(BPin)aniline **3i**.

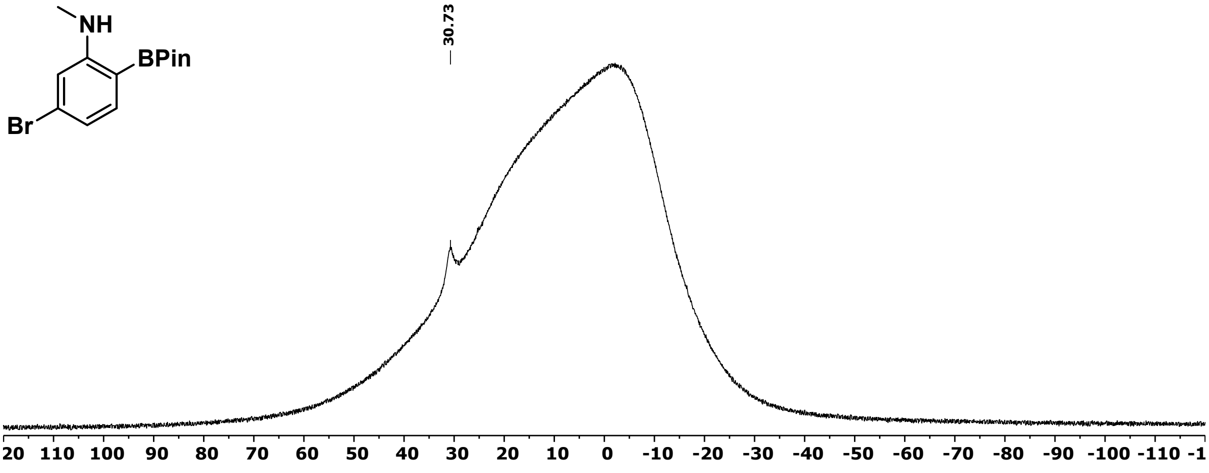

Figure S90: ^{11}B NMR spectrum (160 MHz, $CDCl_3$, 300 K) of the 3-bromo-N-methyl-2-(BPin)aniline **3i**.

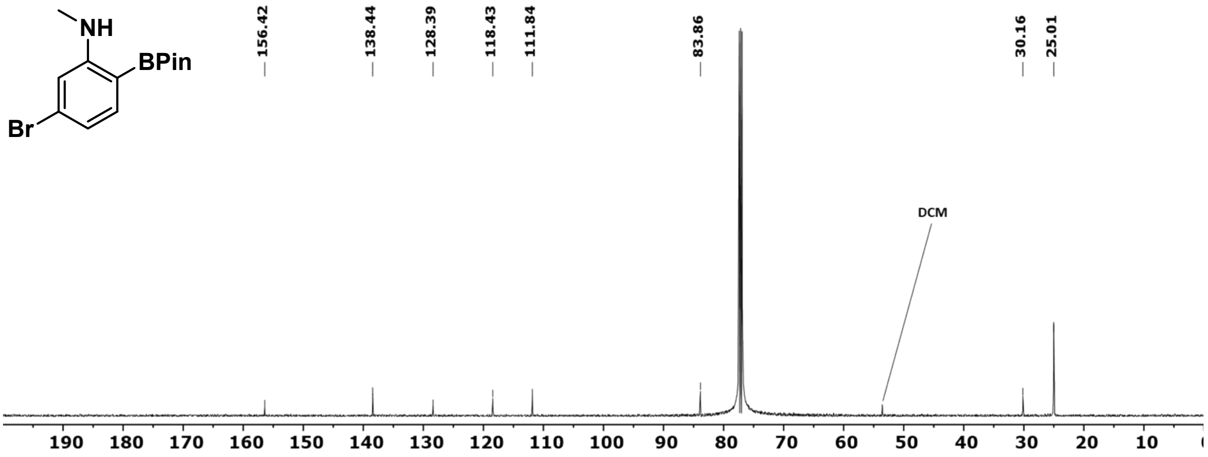

Figure S91: $^{13}C\{^1H\}$ NMR spectrum (126 MHz, $CDCl_3$, 300 K) of the 3-bromo-N-methyl-2-(BPin)aniline **3i**.

9.5.7 NMR spectra of 4-piperidine-N-methyl-2-(BPin)aniline 3j

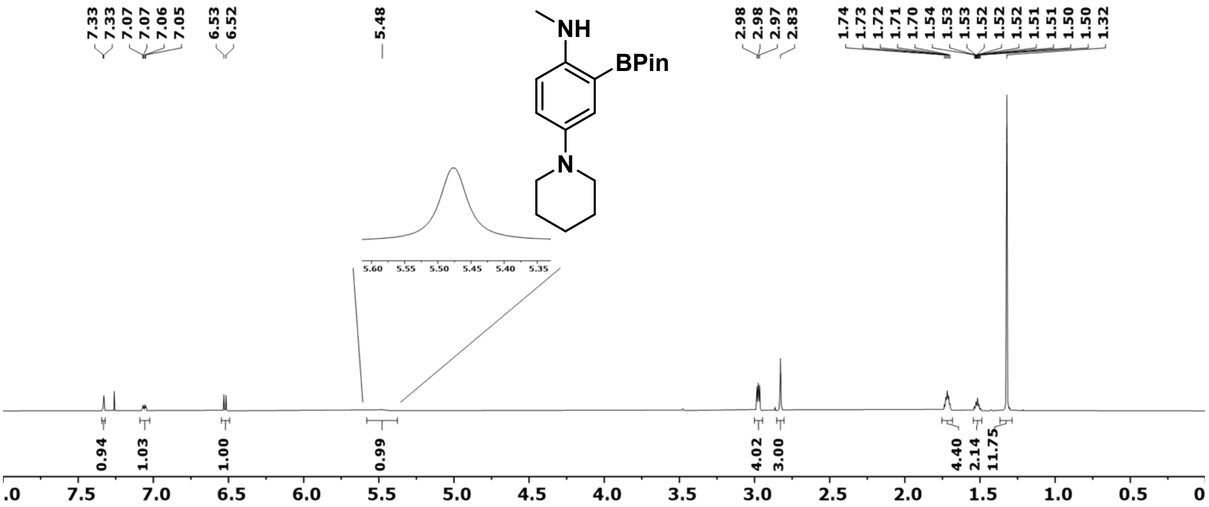

Figure S92: ^1H NMR spectrum (600 MHz, $CDCl_3$, 300 K) of 4-piperidine-N-methyl-2-(BPin)aniline **3j**.

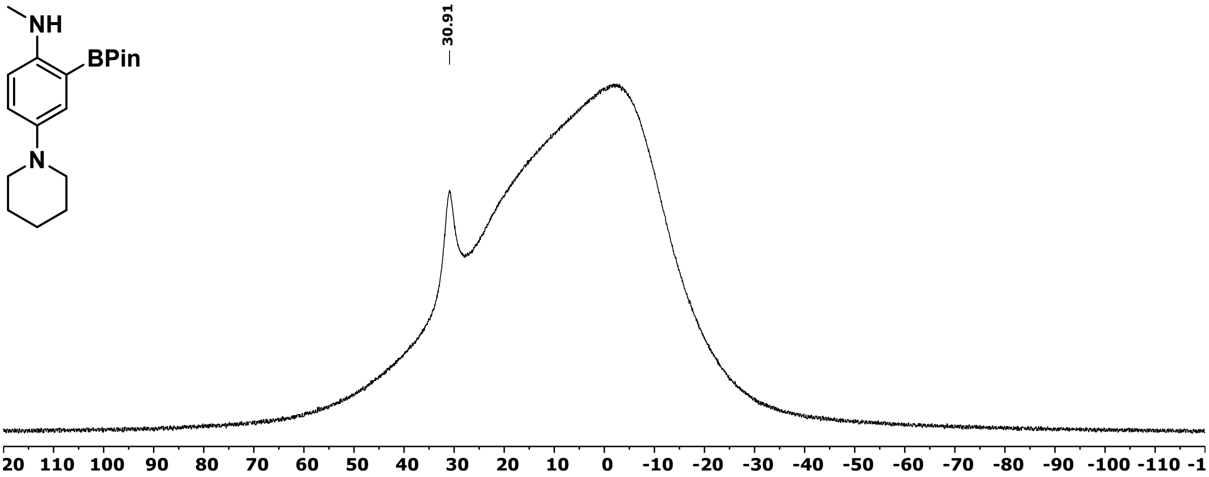

Figure S93: ^{11}B NMR spectrum (160 MHz, $CDCl_3$, 300 K) of 4-piperidine-N-methyl-2-(BPin)aniline **3j**.

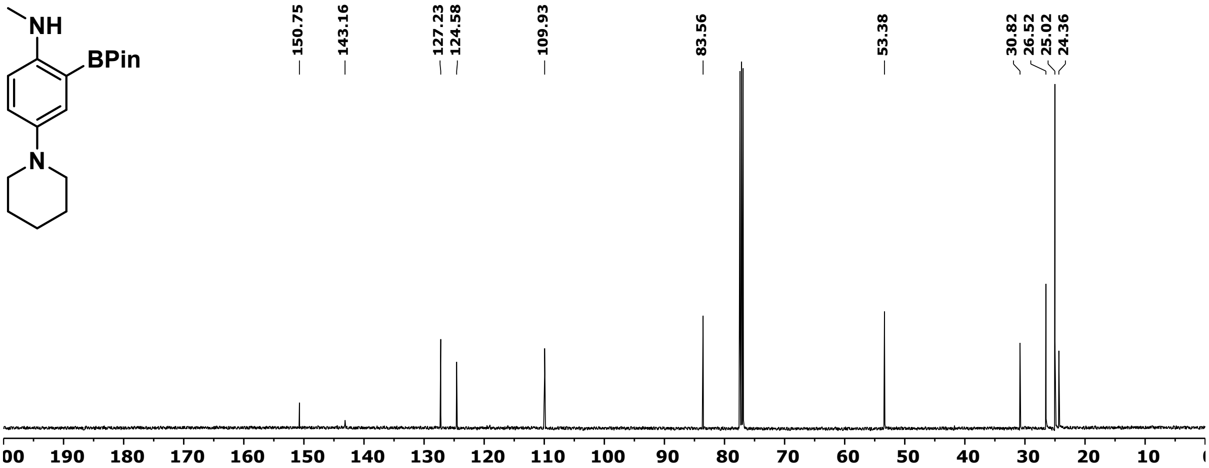

Figure S94: ^{13}C NMR spectrum (126 MHz, $CDCl_3$, 300 K) of 4-piperidine-N-methyl-2-(BPin)aniline **3j**.

10 Crystallographic data

10.1 Crystal structure of di-N-methylaniline-pyrazabole 5

CCDC Deposition Number: 2287755

Experimental: Single colourless plate-shaped crystals of **5** recrystallised from hexane by slow evaporation. A suitable crystal with dimensions 0.07 × 0.04 × 0.01 mm^3 was selected and mounted on a mitegen tip in Paratone oil. On a Diamond Light Source I-19 diffractometer. The crystal was kept at a steady T = 100.00 K during data collection. The structure was solved with the ShelXT 2018/2 (Sheldrick, 2018)[S18] solution program using dual methods and by using Olex2 1.5-beta (Dolomanov et al., 2009)[S16] as the graphical interface. The model was refined with ShelXL 2018/3 (Sheldrick, 2015)[S17] using full matrix least squares minimisation on F^2.

**Compound**	**5**
Formula	$C_{20}H_{24}B_2N_6$
$D_{calc.}$/ g cm^{-3}	1.275
μ/mm^{-1}	0.074
Formula Weight	370.07
Colour	colourless
Shape	plate-shaped
Size/mm^3	0.07×0.04×0.01
T/K	100.00
Crystal System	orthorhombic
Flack Parameter	-0.1(10)
Hooft Parameter	0.5(18)
Space Group	$Pna2_1$
a/Å	11.6629(9)
b/Å	8.3625(7)
c/Å	19.7736(18)
α/°	90
β/°	90
γ/°	90
V/Å^3	1928.5(3)
Z	4
Z'	1
Wavelength/Å	0.6889
Radiation type	synchrotron
Θ_{min}/°	1.997
Θ_{max}/°	24.594
Measured Refl's.	16390
Indep't Refl's	3486
Refl's I≥2 σ(I)	2760
R_{int}	0.0680
Parameters	263
Restraints	1
Largest Peak	0.332

Deepest Hole	-0.253
GooF	0.983
wR_2 (all data)	0.1483
wR_2	0.1396
R_1 (all data)	0.0692
R_1	0.0540

10.2 **Crystal structure of diiodo-pyrazabole 7**

CCDC Deposition Number: 2287757

Experimental: Single colourless plate-shaped crystals of **7** crystallised by layering DCM with hexane. A suitable crystal with dimensions 0.16 × 0.07 × 0.03 mm^3 was selected and mounted on a MITIGEN holder in Paratone oil on a Rigaku Oxford Diffraction Excalibur diffractometer equipped with Eos CCD detector. The crystal was kept at a steady T = 120.0(2) K during data collection. The structure was solved with the ShelXT[S18] solution program using dual methods and by using Olex2 1.5-beta[S16] as the graphical interface. The model was refined with ShelXL 2018/3[S17] using full matrix least squares minimisation on F^2.

**Compound**	**7**
Formula	$C_6H_8B_2I_2N_4$
$D_{calc.}$/ g cm^{-3}	2.389
μ/mm^{-1}	5.461
Formula Weight	411.58
Colour	colourless
Shape	plate-shaped
Size/mm^3	0.163x0.072x0.029
T/K	120.00(10)
Crystal System	triclinic
Space Group	P-1
a/Å	7.6116(5)
b/Å	7.7121(7)
c/Å	10.8808(7)
α/°	102.544(7)
β/°	91.351(5)
γ/°	112.430(7)
V/Å^3	572.28(8)
Z	2
Wavelength/Å	0.71073
Radiation type	Mo K$_\alpha$
Θ_{min}/°	6.444
Θ_{max}/°	57.756
Measured Refl's.	4678
Indep't Refl's	4678
R_Θ	0.0270
Parameters	127

Restraints	0
Largest Peak	1.64
Deepest Hole	-0.84
GooF	1.036
wR_2 (all data)	0.1097
wR_2	0.1064
R_1 (all data)	0.0490
R_1	0.0408

10.3 **Crystal structure of mono-cation 8**

CCDC Deposition Number: 2287754

Experimental: Single colourless plate-shaped crystals of **8** recrystallised from difluorobenzene by slow cooling. A suitable crystal with dimensions 0.05 × 0.01 × 0.01 mm^3 was selected and mounted on a MITIGEN holder in Paratone oil on a Fluid Film Devices diffractometer. The crystal was kept at a steady T = 100.00 K during data collection. The structure was solved with the ShelXT 2018/2 (Sheldrick, 2018)[S18] solution program using dual methods and by using Olex2 1.5-beta (Dolomanov et al., 2009)[S16] as the graphical interface. The model was refined with ShelXL 2018/3 (Sheldrick, 2015)[S17] using full matrix least squares minimisation on F^2.

**Compound**	**8**
Formula	$C_{12}H_{23}B_2N_5I_2$
$D_{calc.}$/ g cm^{-3}	1.838
μ/mm^{-1}	10.039
Formula Weight	512.77
Colour	colourless
Shape	plate-shaped
Size/mm^3	0.05×0.01×0.01
T/K	100.00
Crystal System	monoclinic
Space Group	$P2_{1/c}$
a/Å	10.2464(16)
b/Å	14.259(2)
c/Å	13.0316(19)
α/°	90
β/°	103.321(4)
γ/°	90
V/Å^3	1852.7(5)
Z	4
Z'	1
Wavelength/Å	1.0402
Radiation type	synchrotron
Θ_{min}/°	2.990

$\Theta_{max}/^\circ$	31.662
Measured Refl's.	6651
Indep't Refl's	1936
Refl's I≥2 σ(I)	1306
R_{int}	0.0821
Parameters	199
Restraints	164
Largest Peak	1.778
Deepest Hole	-1.357
GooF	1.017
wR_2 (all data)	0.2213
wR_2	0.2051
R_1 (all data)	0.1085
R_1	0.0778

10.4 **Crystal structure of compound 9**

CCDC Deposition Number: 2287756

Experimental: Single colourless blade-shaped crystals of **9** recrystallised from PhCl by slow evaporation. A suitable crystal with dimensions 0.29 × 0.05 × 0.02 mm^3 was selected and mounted on a MITIGEN holder in Paratone oil on a Bruker D8 VENTURE diffractometer. The crystal was kept at a steady T = 100.0 K during data collection. The structure was solved with the ShelXT 2018/2 (Sheldrick, 2018)[S18] solution program using dual methods and by using Olex2 1.5-beta (Dolomanov et al., 2009)[S16] as the graphical interface. The model was refined with ShelXL 2018/3 (Sheldrick, 2015)[S17] using full matrix least squares minimisation on F^2.

**Compound**	**9**
Formula	$C_9H_{11}B_2N_6I$
$D_{calc.}$/ g cm^{-3}	1.726
μ/mm^{-1}	2.356
Formula Weight	351.76
Colour	colourless
Shape	blade-shaped
Size/mm^3	0.29×0.05×0.02
T/K	100.0
Crystal System	monoclinic
Space Group	$C2/c$
a/Å	15.8673(12)
b/Å	11.1350(8)
c/Å	15.4788(11)
$\alpha/^\circ$	90
$\beta/^\circ$	98.150(4)
$\gamma/^\circ$	90
V/Å^3	2707.2(3)
Z	8

Z'	1
Wavelength/Å	0.71073
Radiation type	MoK_{α}
$\Theta_{min}/^{\circ}$	2.242
$\Theta_{max}/^{\circ}$	26.460
Measured Refl's.	24720
Indep't Refl's	2759
Refl's I≥2 σ(I)	2440
R_{int}	0.0528
Parameters	163
Restraints	54
Largest Peak	2.185
Deepest Hole	-2.620
GooF	1.346
wR_2 (all data)	0.1755
wR_2	0.1710
R_1 (all data)	0.0845
R_1	0.0752

11 Computational details

All geometry optimizations were run with Gaussian16 (Revision A.03)[S19] using the MN15 functional.[S20] Geometries were optimized with a LANL2DZ basis set for I and 6-311G(d,p) basis set for the rest of the atoms. All geometry optimizations were full, with no restrictions. All stationary points were characterized as minima by vibrational analysis. Solvent effects of chlorobenzene were introduced using the self-consistent field approach, by means of the integral equation formalism polarizable continuum model (IEFPCM).[S21]

Computed geometries and energies (atomic units) are shown below.

11.1 **Comparison of *cis/trans*-diiodo-pyrazabole 7 isomer**

11.1.1 Trans-diiodo-pyrazabole isomer (trans-7)

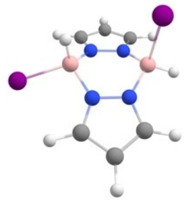

ΔH(298 K) = –524.35665

ΔG(298 K) = –524.41273

B	1.49958	-0.83354	0.19200
N	0.02416	-1.08018	-0.10287
C	-0.76761	-0.48362	-1.00542
C	-2.06014	-0.95939	-0.85090
H	-0.35404	0.23677	-1.69312
C	-1.98446	-1.87525	0.19249
H	-2.93167	-0.69256	-1.42275
H	-2.74690	-2.49284	0.63860
N	2.08952	-2.15195	0.68020
C	3.34089	-2.61579	0.55257
C	3.44500	-3.81679	1.23642

H	4.06748	-2.05778	-0.01633
C	2.18075	-4.03737	1.77140
H	4.30972	-4.45122	1.32305
H	1.80410	-4.85551	2.36348
N	-0.72123	-1.93814	0.63274
N	1.37384	-3.02563	1.42682
B	-0.08484	-2.67991	1.84339
H	-0.11556	-2.01856	2.83098
I	-1.22805	-4.58710	2.24874
H	2.07863	-0.40472	-0.74884
I	1.67442	0.73868	1.88204

11.1.2 Cis-diiodo-pyrazabole isomer (cis-7)

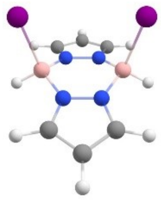

ΔH(298 K) = –524.35768

ΔG(298 K) = –524.41433

B	1.52683	-0.86448	0.07786
I	1.93649	0.93331	1.46080
N	0.01969	-1.06243	-0.09753
C	-0.80251	-0.42665	-0.94351
C	-2.09738	-0.88760	-0.74930
H	-0.40926	0.31074	-1.62532
C	-1.99105	-1.83993	0.25504
H	-2.98687	-0.58330	-1.27266
H	-2.73895	-2.45966	0.72398
H	2.05259	-0.60478	-0.95374

N	2.10161	-2.14277	0.69167
C	3.36402	-2.58896	0.63604
C	3.45887	-3.77135	1.35709
H	4.10833	-2.03387	0.08722
C	2.17624	-4.00261	1.83485
H	4.33250	-4.38244	1.50211
H	1.77991	-4.80494	2.43683
N	-0.71012	-1.93031	0.63840
N	1.37223	-3.01078	1.42780
B	-0.11558	-2.82065	1.73190
H	-0.68025	-3.86240	1.79632
I	-0.38858	-1.83425	3.79712

11.2 **Calculated dicationic dimeric structure ([4]$^{2+}$)**

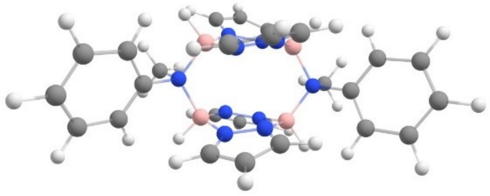

ΔH(298 K) = –1654.648831

ΔG(298 K) = –1654.736890

B	3.35885	-1.57312	5.20958
N	3.25045	-2.51327	3.98460
C	4.29432	-3.09797	3.37495
C	3.83884	-3.91278	2.35163
H	5.29909	-2.91545	3.72000
C	2.46054	-3.82456	2.42557
H	4.42418	-4.51988	1.68382

H	1.70960	-4.37473	1.88224
H	4.24821	-2.03184	5.85262
N	1.99688	-1.64486	5.97642
C	1.74051	-1.28038	7.24796
C	0.57876	-1.88945	7.68954
H	2.40265	-0.62108	7.78142
C	0.15042	-2.64995	6.61588
H	0.12145	-1.80104	8.65935
H	-0.71092	-3.28987	6.51674
N	2.10936	-2.96492	3.40146
N	0.99790	-2.48854	5.58762
B	0.74930	-2.98893	4.15335
H	0.40061	-4.12269	4.18349
N	3.78735	-0.06680	4.80856
H	-0.98269	-0.08411	2.61166
H	3.17224	1.79845	3.64454
B	-0.05848	-0.57356	3.17845
B	2.70609	0.74193	3.91689
N	0.25853	0.17343	4.51557
N	1.13732	-0.34707	2.22172
N	2.39260	0.03992	2.56722
N	-0.39964	-2.13702	3.39839
C	2.30415	-0.08810	0.33925
H	2.57252	-0.03938	-0.70136
C	3.09561	0.21605	1.43216
H	4.09810	0.61134	1.46714
C	1.06890	-0.40313	0.88155
H	0.13805	-0.64260	0.39237
C	1.19443	1.76245	5.70958
H	1.96328	2.46292	5.99215

C	-0.04429	1.51918	6.27650
H	-0.48699	2.00677	7.12727
C	-0.59900	0.51992	5.49536
H	-1.56492	0.05227	5.57795
N	1.37477	0.94253	4.66218
C	5.04031	-0.16189	3.98123
H	5.36902	0.84445	3.72215
H	5.81686	-0.65165	4.57116
H	4.85877	-0.72704	3.07157
C	-0.56415	-2.75952	2.03921
C	4.23461	0.73960	5.99926
C	4.47469	2.11048	5.85086
C	4.57848	0.13748	7.20942
C	4.96012	2.86625	6.91160
H	4.29401	2.61082	4.91111
C	5.06429	0.90069	8.27073
H	4.49900	-0.92857	7.35355
C	5.24479	2.26956	8.13552
H	5.12408	3.92703	6.76776
H	5.31081	0.40405	9.20110
H	5.62266	2.85950	8.96105
C	-1.75347	-2.34652	4.02433
C	-2.12508	-3.62714	4.44963
C	-2.72013	-1.34089	4.03948
C	-3.39830	-3.86819	4.95269
H	-1.43710	-4.45753	4.39130
C	-3.99612	-1.58882	4.54448
H	-2.51987	-0.35568	3.64830
C	-4.34056	-2.84705	5.01669
H	-3.64984	-4.86787	5.28479

H	-4.71975	-0.78301	4.55129
H	-5.33218	-3.03640	5.40803
H	0.35632	-2.68481	1.46731
H	-0.82942	-3.80933	2.16365
H	-1.36901	-2.24574	1.51200

11.3 **Calculation of iodide/NTf_2 exchange**

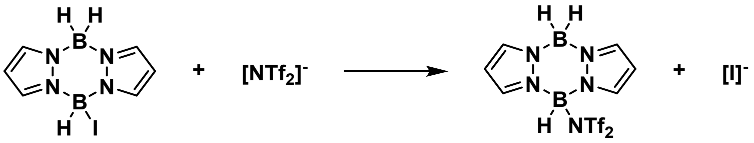

$\Delta G_{Exchange}$(298 K) = 0.011953 Hartree per particle = 7.5 kcal.mol^{-1}

11.3.1 Anion [NTf2]$^{-}$

ΔH(298 K) = –1826.464685

ΔG(298 K) = –1826.522051

S	-0.48923	-0.16504	3.36446
S	-2.65930	-0.92544	1.71540
C	-1.13490	-0.75135	4.99244
C	-3.37817	-2.55426	2.19953
F	-2.43357	-0.50809	5.09835
F	-0.49688	-0.11649	5.97074
F	-0.92635	-2.05565	5.12175
F	-3.32977	-2.70529	3.51724
F	-4.64751	-2.59735	1.80851
F	-2.71641	-3.54740	1.62369
O	-3.49608	0.05636	2.37398

O	-2.63706	-0.96676	0.26897
O	0.91422	-0.50927	3.41509
O	-0.87378	1.22899	3.30292
N	-1.19696	-1.10940	2.30946

11.3.2 Anion Iodide

ΔH(298 K) = –11.495772

ΔG(298 K) = –11.514981

I	0.22393	-0.97257	0.01174

11.3.3 Mono-NTf$_2$-pyrazabole

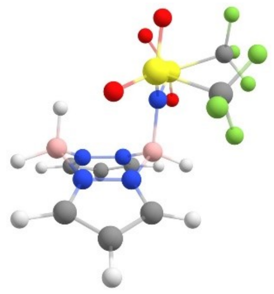

ΔH(298 K) = –2328.543482

ΔG(298 K) = –2328.620294

C	1.70120	2.95817	0.31482
H	1.69404	3.97051	-0.05676
C	2.16569	0.95669	1.11056
H	2.58667	0.05068	1.51341
C	2.74984	2.16926	0.79114
H	3.78646	2.44311	0.88342
C	-1.50307	-0.83179	-1.28888
H	-1.34490	-1.88430	-1.12229

C	-2.14754	-0.16719	-2.31519
H	-2.64054	-0.59940	-3.16848
C	-1.99340	1.18825	-2.01718
H	-2.32126	2.06593	-2.55100
N	0.57171	2.25536	0.35949
N	0.84752	1.02821	0.85457
N	-1.01377	0.07738	-0.42233
N	-1.30822	1.31379	-0.88541
B	-0.21350	-0.09014	0.87997
B	-0.88748	2.58353	-0.07234
H	-1.57844	2.68827	0.90321
H	-0.89397	3.53744	-0.79451
H	0.31438	-1.15475	0.94013
S	-0.54121	0.18157	3.66705
S	-2.89167	0.00354	2.01480
C	-0.95960	-1.43602	4.51284
C	-3.19085	-1.81138	1.60356
F	-2.12803	-1.40681	5.11026
F	-0.00942	-1.64418	5.40492
F	-0.93521	-2.40288	3.60779
F	-3.59832	-2.43222	2.69232
F	-4.12176	-1.87329	0.67265
F	-2.08615	-2.38951	1.15511
O	-3.45712	0.20147	3.31996
O	-3.36275	0.72083	0.86821
O	0.87746	0.06545	3.45740
O	-1.10540	1.24231	4.44717
N	-1.23337	0.08042	2.14468

11.3.4 Monoiodo-pyrazabole

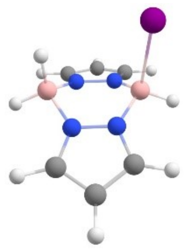

ΔH(298 K) = –513.574744

ΔG(298 K) = –513.625177

B	1.50047	-0.80463	0.22911
I	1.72079	0.82073	1.86919
N	0.01936	-1.04404	-0.05484
C	-0.76824	-0.49669	-0.99604
C	-2.04420	-1.02017	-0.86778
H	-0.36315	0.22059	-1.69179
C	-1.95821	-1.91593	0.19758
H	-2.90860	-0.79754	-1.46896
H	-2.70504	-2.55611	0.63939
H	2.07433	-0.39604	-0.72537
N	2.09251	-2.11947	0.73144
C	3.32696	-2.62118	0.55762
C	3.40295	-3.84733	1.19744
H	4.05947	-2.07305	-0.01306
C	2.13701	-4.04246	1.74883
H	4.24810	-4.51190	1.24463
H	1.73931	-4.86471	2.32195
N	-0.71252	-1.91280	0.67373
N	1.36665	-2.99266	1.46086
B	-0.09036	-2.64486	1.90642
H	-0.05288	-1.89147	2.84226
H	-0.69526	-3.65819	2.11338

11.4 **Calculation of the di-N-alkyl-anilines-pyrazabole**

11.4.1 Di-N-methylaniline-pyrazabole

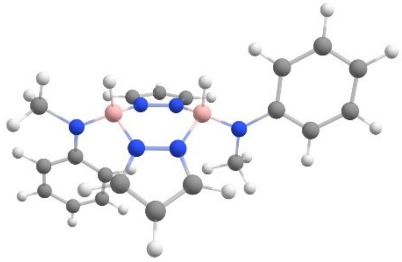

ΔH(298 K) = –1153.362278

ΔG(298 K) = –1153.443025

N	0.87007	-1.11434	-1.00882
N	-0.40141	-1.25746	-0.59429
N	0.7578	1.33238	-0.55412
N	-0.50701	1.1766	-0.12847
N	2.94724	0.31376	-1.35376
N	-2.47493	-0.27839	0.45114
C	-0.62608	-2.55298	-0.34101
H	-1.59794	-2.87523	-0.00124
C	0.53885	-3.2695	-0.59303
H	0.69628	-4.32815	-0.47835
C	1.45981	-2.31517	-1.0118
H	2.4962	-2.40521	-1.29768
C	-0.84218	2.23521	0.61991
H	-1.82754	2.29469	1.05662
C	0.24301	3.10344	0.6801
H	0.30614	4.04008	1.20684
C	1.23511	2.48691	-0.07536
H	2.25114	2.7802	-0.28894
C	3.62684	0.11806	-0.15175
C	5.0342	0.16469	-0.08898

H	5.6131	0.35491	-0.98275
C	5.71271	-0.03065	1.11116
H	6.79669	0.01426	1.11139
C	5.0263	-0.27905	2.2936
H	5.55792	-0.43033	3.22532
C	3.63379	-0.32878	2.24875
H	3.06853	-0.52096	3.15448
C	2.94403	-0.13628	1.06003
H	1.86214	-0.18561	1.07813
C	3.75375	0.56525	-2.53771
H	4.43421	-0.26739	-2.7565
H	4.364	1.47133	-2.43473
H	3.09299	0.6967	-3.39185
C	-3.84203	-0.10188	0.2374
C	-4.33369	0.63411	-0.85956
H	-3.63609	1.09036	-1.5499
C	-5.6971	0.80146	-1.05727
H	-6.03597	1.37572	-1.91314
C	-6.62682	0.26282	-0.16798
H	-7.68978	0.40188	-0.32481
C	-6.15484	-0.44678	0.93024
H	-6.85337	-0.87323	1.6425
C	-4.78943	-0.62838	1.13532
H	-4.4621	-1.19925	1.99542
C	-2.05212	-0.64471	1.79541
H	-2.2821	-1.68686	2.0532
H	-2.52666	-0.00468	2.54882
H	-0.97023	-0.51845	1.88032
B	1.4598	0.27198	-1.48619
H	1.10095	0.46382	-2.62014
B	-1.41223	-0.04413	-0.56811

H	-1.80349	0.13753	-1.68793

11.4.2 Di-2,N-dimethylaniline-pyrazabole

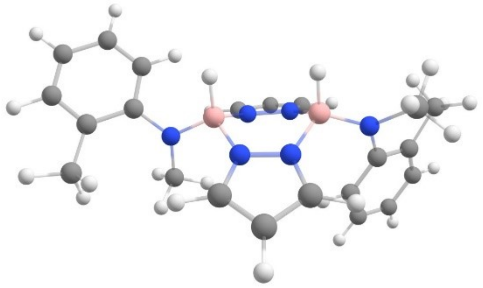

ΔH(298 K) = –1231.838972

ΔG(298 K) = –1231.923419

C	-5.08663	0.34946	0.42164
C	-3.88294	-0.27882	0.03886
C	-3.95301	-1.46985	-0.70022
C	-5.17199	-2.04561	-1.03847
C	-6.35905	-1.44715	-0.62718
C	-6.3003	-0.25785	0.09207
N	-2.62959	0.2716	0.37825
B	-1.4572	0.15491	-0.5271
N	-0.59044	1.47012	-0.46628
N	0.71644	1.45543	-0.7771
C	1.19114	2.7039	-0.69714
C	0.15659	3.55571	-0.32162
C	-0.95352	2.7281	-0.18708
B	1.43568	0.13609	-1.31495
N	2.9187	0.20756	-1.24372
C	3.60038	0.71386	-2.43359
C	3.66984	0.06102	-0.06034
C	4.88834	-0.65509	-0.06409

C	5.61392	-0.76587	1.12377
C	5.15207	-0.23216	2.32258
C	3.92068	0.41537	2.33901
C	3.19457	0.56125	1.16254
C	-2.38502	0.57984	1.78653
N	0.84971	-0.99711	-0.40757
N	-0.44011	-0.96443	-0.02551
C	-0.70002	-2.06367	0.6933
C	0.45553	-2.83401	0.77464
C	1.41148	-2.11801	0.06243
H	-1.68832	-2.21993	1.09963
H	0.58574	-3.77153	1.28724
H	2.45374	-2.32437	-0.12704
H	-1.97357	2.94572	0.08935
H	0.20648	4.61891	-0.16044
H	2.23412	2.8982	-0.89706
C	5.35313	-1.39694	-1.29029
H	6.54843	-1.31956	1.10672
H	5.73236	-0.34231	3.23126
H	3.52529	0.81881	3.26472
H	2.24087	1.07521	1.18572
H	3.81427	-0.06093	-3.17886
H	4.54517	1.18838	-2.15517
H	2.98434	1.47655	-2.92065
H	-3.02802	-1.94819	-1.00578
H	-5.1902	-2.96691	-1.61012
H	-7.31677	-1.8895	-0.87582
H	-7.22103	0.23836	0.3856
C	-5.08844	1.69792	1.09215
H	-3.01899	-0.03625	2.43281
H	-2.56156	1.62939	2.04984

H	-1.34273	0.35622	2.03545
H	1.04832	-0.02366	-2.45035
H	-1.74544	-0.07801	-1.67253
H	-6.05916	2.18093	0.96664
H	-4.88345	1.63852	2.16497
H	-4.32152	2.34276	0.65172
H	6.05828	-2.18165	-1.01032
H	4.49989	-1.8585	-1.7982
H	5.84758	-0.75236	-2.02238

11.4.3 Di-indoline-pyrazabole

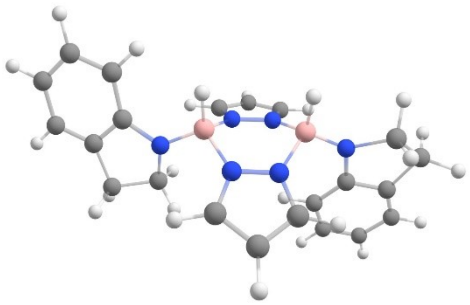

ΔH(298 K) = −1229.503063

ΔG(298 K) = −1229.582946

C	-4.73416	0.51177	0.8719
C	-3.92027	0.02904	-0.16868
C	-4.46537	-0.78343	-1.16348
C	-5.82881	-1.08429	-1.10498
C	-6.63751	-0.60389	-0.07864
C	-6.08164	0.20589	0.91943
N	-2.60538	0.46472	-0.01095
B	-1.44441	0.03876	-0.83388
N	-0.3937	1.20717	-0.91436

N	0.91566	0.97294	-1.11065
C	1.55568	2.1453	-1.18514
C	0.63004	3.17371	-1.03947
C	-0.59142	2.53096	-0.87108
B	1.49367	-0.48461	-1.33984
N	2.93957	-0.56031	-1.01927
C	3.9849	-0.57879	-2.05122
C	3.53447	-0.16541	0.17548
C	4.93611	-0.23462	0.0857
C	5.73109	0.1138	1.16158
C	5.12821	0.54386	2.35091
C	3.74051	0.60609	2.43888
C	2.92469	0.2565	1.35911
C	-2.47227	0.93976	1.37538
N	0.65781	-1.36933	-0.34472
N	-0.64746	-1.13522	-0.12471
C	-1.12023	-2.08348	0.69372
C	-0.08498	-2.95418	1.01918
C	1.02338	-2.45964	0.33977
H	-2.15979	-2.0765	0.98644
H	-0.12947	-3.81507	1.66385
H	2.04671	-2.79848	0.30217
H	-1.58644	2.91989	-0.71981
H	0.81932	4.23334	-1.04383
H	2.62572	2.17003	-1.32519
C	5.2892	-0.7977	-1.2701
H	6.81226	0.05359	1.08602
H	5.73983	0.82708	3.19949
H	3.27717	0.94091	3.36079
H	1.84488	0.32281	1.44028
H	-3.84668	-1.17851	-1.9603

H	-6.25952	-1.71782	-1.87313
H	-7.69035	-0.85905	-0.05008
H	-6.7017	0.58903	1.72383
C	-3.88728	1.3962	1.75337
H	1.25771	-0.81613	-2.47613
H	-1.74278	-0.31322	-1.94651
H	4.02395	0.38925	-2.57486
H	3.78636	-1.35305	-2.79329
H	5.51287	-1.86745	-1.18193
H	6.1461	-0.31478	-1.74171
H	-1.72595	1.7323	1.45676
H	-2.15756	0.11751	2.03687
H	-4.0924	1.29067	2.81943
H	-4.03799	2.4483	1.48231

11.4.4 Di-tetrahydroquinoline-pyrazabole

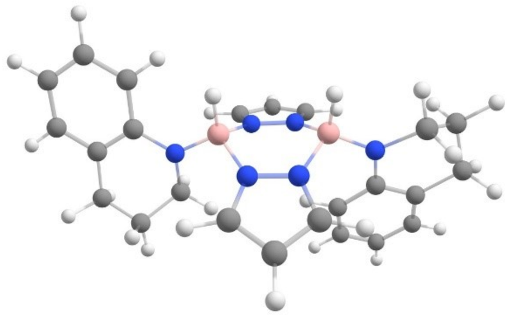

ΔH(298 K) = –1307.987898

ΔG(298 K) = –1308.072001

C	-4.99737	0.53651	0.38708
C	-3.9346	-0.2732	-0.07382
C	-4.23836	-1.58372	-0.49089
C	-5.54148	-2.06129	-0.49356
C	-6.59131	-1.25494	-0.05986
C	-6.29749	0.03028	0.37828
N	-2.62285	0.21654	-0.08445
B	-1.44974	-0.48937	-0.6737
N	-0.49316	0.53615	-1.39835
N	0.82502	0.30672	-1.52821
C	1.36648	1.29663	-2.24671
C	0.36505	2.19428	-2.60175
C	-0.79553	1.67013	-2.04344
B	1.53719	-0.99984	-0.98644
N	2.98968	-0.77879	-0.73559
C	3.96067	-1.45973	-1.58817
C	3.49748	0.15287	0.17695
C	4.88614	0.24204	0.4333
C	5.35889	1.18188	1.34849
C	4.51211	2.04718	2.03056
C	3.14377	1.95337	1.79244
C	2.64554	1.02634	0.88707
C	-2.37595	1.34921	0.80379
N	0.7641	-1.34243	0.33962
N	-0.54748	-1.08851	0.4831
C	-0.93777	-1.51998	1.68965
C	0.15929	-2.06564	2.34824
C	1.21644	-1.92649	1.45498

H	-1.96868	-1.41339	1.99243
H	0.18718	-2.4901	3.33707
H	2.26074	-2.1846	1.54139
H	-1.81438	2.02395	-2.05872
H	0.4672	3.10054	-3.17358
H	2.42744	1.29981	-2.44475
C	5.87005	-0.68662	-0.24275
H	6.43	1.21972	1.52978
H	4.90759	2.76815	2.7361
H	2.4511	2.60607	2.31263
H	1.57469	0.9851	0.72866
H	-3.43473	-2.23611	-0.80666
H	-5.73182	-3.07628	-0.82554
H	-7.61124	-1.62086	-0.05715
H	-7.0978	0.67825	0.72689
C	-4.76631	1.94269	0.89913
H	1.34895	-1.88367	-1.78482
H	-1.71301	-1.36114	-1.4563
C	-3.38054	2.46155	0.53931
H	-1.34951	1.69479	0.65686
H	-2.46087	1.04117	1.85933
H	-3.33876	2.73324	-0.52099
H	-3.13678	3.35342	1.12183
H	-4.87223	1.94523	1.99118
H	-5.547	2.60506	0.5134
C	5.16426	-1.92118	-0.78241
H	6.65944	-0.95525	0.46536
H	6.36127	-0.16616	-1.0746
H	4.81516	-2.55348	0.04236
H	5.83631	-2.51806	-1.40363
H	4.31161	-0.78993	-2.39066

H	3.45694	-2.30027	-2.0655

11.4.5 Angle comparison in compound 5 and its analogues

Di-N-methylaniline-pyrazabole

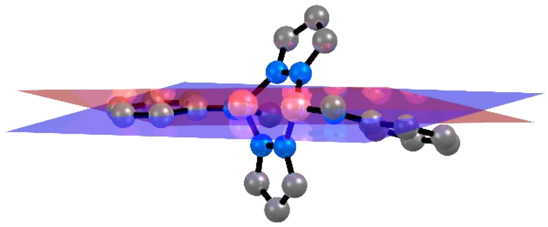

17.45°

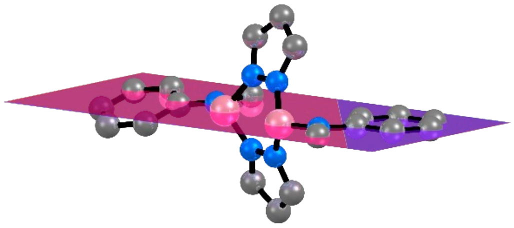

0.10°

Di-2,N-dimethylaniline-pyrazabole

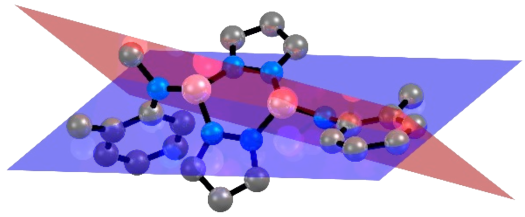

44.15°

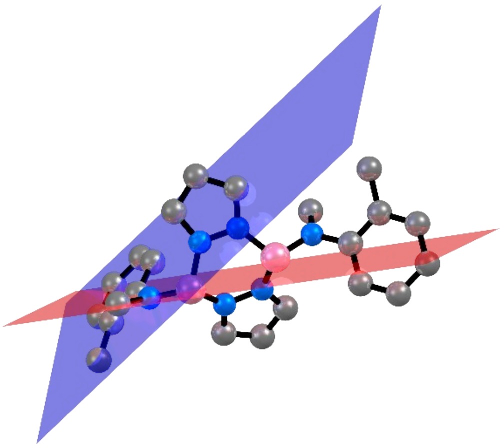

44.22°

Di-indoline-pyrazabole

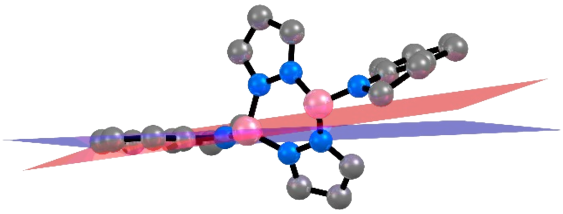

10.15°

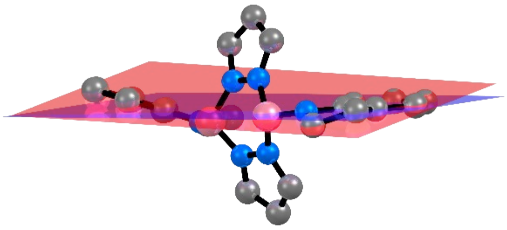

8.73°

Di-tetrahydroquinoline-pyrazabole

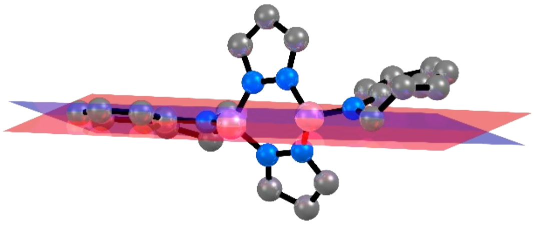

15.35°

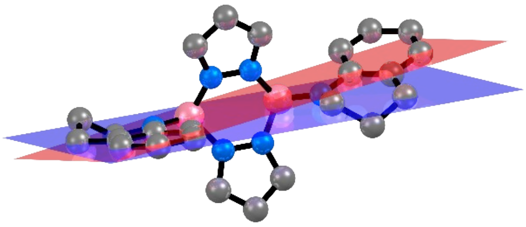

8.22°

Table S4: Angles between the plane made by N and its three bonded atoms (C, C and B) (red) and the phenyl plane of the aniline derivative (blue) for the calculated structures.

The calculated structures revealed larger angles between the plane centered on N and the aniline phenyl plane (see Table S4) for the di-(2,N-dimethylaniline)-pyrazabole (44.15° and 44.22°) compared with the other substrates (where the angles were found to be between 0° and 18°).

This difference in angles is consistent with the difference in reactivity observed between the 2,N-dimethylaniline, which did not show any *ortho*-borylated product, and the other ortho functionalised substrates (N-methylaniline, indoline and tetrahydroquinoline), which gave good yields.

The larger twist angles observed in the case of the di-2,N-dimethylaniline-pyrazabole indicates a weaker conjugation between the Nitrogen lone pair and the phenyl ring. This will reduce the nucleophilicity of the aniline aromatic ring and disfavour its *ortho*-borylation via an S_EAr mechanism.

Note, the values of the calculated angles between the planes shown above for (N-methylaniline)-pyrabole **5** (17.45° and 0.10°) were found to be similar to the measured values from the solid-state structure of **5** (10.04° and 4.20°), with packing effects presumably accounting for the small difference in values.

12 References

S1. Pahl J., Noone E., Uzelac M. , Yuan K., Ingleson M. J.; *Angew. Chem. Int. Ed.* **2022**,61,e202206230.

S2. Niedenzu K., Nöth H.; Pyrazabole: *Chem. Ber.* **1983**, 116, 1132-1153.

S3. Layton W. J., Niedenzu K., Niedenzu P. M., Trofimenko S., *Inorg. Chem.* **1985**, 24, 10, 1454-1457.

S4. Hanecker E., Hodgkins T. G., Niedenzu K., Nöth H.; *Inorg. Chem.* **1985**, 24, 4, 459-462.

S5. Kresiński R. A.; *J. Chem. Soc., Dalton Trans.*, **1999**, 401-405.

S6. Kresiński R. A., Jones C. J., McCleverty J. A.; *Polyhedron* **1990**, 9, 2185-2187.

S7. Bielawski J., Das M. K., Hanecker E., Niedenzu K., Nöth H., *Inorg. Chem.* **1986**, 25, 26, 4623-4628.

S8. Mao C., Wang Z., Ji P., Cheng J-P., *J. Org. Chem.* **2015**, 80, 8384-8389.

S9. Jiao J., Xiao F., Wang C., Zhang Z., *J. Org. Chem.* **2022**, 87, 4965-4970.

S10. Neufeld R., Stalke D., *Chem. Sci.,* **2015**, 6, 3354-3364.

S11. Bachmann S., Neufeld R., Dzemski M., Stalke D, *Chem. Eur. J.,* **2016**, 22, 8462-8465.

S12. Augé S., Schmit P-O., Crutchfield C. A., Islam M. T., Harris D. J., Durand E., Clemancey M., Quoineaud A-A., Lancelin J-M., Prigent Y., Taulelle F., Delsuc M-A, *J. Phys. Chem. B* **2009**, 113, 1914–1918.

S13. Wrackmeyer B., Molla E., Thoma P., Klimkina E. V., Tok O. L., Bauer T., Kempe R.; *Z. Anorg. Allg .Chem.* **2011**, 637, 401-405.

S14. Lam H., Tsoung J., Lautens M., *J. Org. Chem.* **2017**, 82, 6089-6099.

S15. Robbins D. W., Boebel T. A., Hartwig J. F. *J. Am. Chem. Soc.* **2010**, 132, 12, 4068-4069.

S16. Dolomanov O. V., Bourhis L. J., Gildea R. J., Howard J. A. K., Puschmann H.; *J. Appl. Cryst.* **2009**, 42, 339-341.

S17. Sheldrick G.M.; *Acta Cryst.* **2015**, C71, 3-8.

S18. Sheldrick G.M.; SHELXT ; *Acta Cryst.* **2015**, A71, 3-8.

S19. Frisch, M. J.; Trucks, G. W.; Schlegel, H. B.; Scuseria, G. E.; Robb, M. A.; Cheeseman, J. R.; Scalmani, G.; Barone, V.; Petersson, G. A.; Nakatsuji, H.; Li, X.; Caricato, M.; Marenich, A. V.; Bloino, J.; Janesko, B. G.; Gomperts, R.; Mennucci, B.; Hratchian, H. P.; Ortiz, J. V.; Izmaylov, A. F.; Sonnenberg, J. L.; Williams-Young, D.; F. Ding, F. L.; F. Egidi, J. G.; B. Peng, A. P.; Henderson, T.; Ranasinghe, D.; Zakrzewski, V. G.; Gao, J.; Rega, N.; Zheng, G.; Liang, W.; Hada, M.; Ehara, M.; Toyota, K.; Fukuda, R.;

Hasegawa, J.; Ishida, M.; Nakajima, T.; Honda, Y.; Kitao, O.; Nakai, H.; Vreven, T.; Throssell, K.; J. A. Montgomery, J.; Peralta, J. E.; Ogliaro, F.; Bearpark, M. J.; Heyd, J. J.; Brothers, E. N.; Kudin, K. N.; Staroverov, V. N.; Keith, T. A.; Kobayashi, R.; Normand, J.; Raghavachari, K.; Rendell, A. P.; Burant, J. C.; Iyengar, S. S.; Tomasi, J.; Cossi, M.; Millam, J. M.; Klene, M.; Adamo, C.; Cammi, R.; Ochterski, J. W.; Martin, R. L.; Morokuma, K.; Farkas, O.; Foresman, J. B.; Fox, D. J. Gaussian 16, Revision A.03 Gaussian Inc.: Wallingford CT, **2016**.

S20. Yu H., He X., Li S. L., Truhlar D. G.; *Chem. Sci.* **2016**, 7, 5032-5051

S21. Mennucci B.; Cancès E.; Tomasi J.; *J. Phys. Chem. B* **1997**, 101, 10506-10517.
